# Carbon storage in China's forest ecosystems: estimation by different integrative methods

**DOI:** 10.1002/ece3.2114

**Published:** 2016-04-03

**Authors:** Shunlei Peng, Ding Wen, Nianpeng He, Guirui Yu, Anna Ma, Qiufeng Wang

**Affiliations:** ^1^ Key Laboratory of Ecosystem Network Observation and ModelingInstitute of Geographic Sciences and Natural Resources ResearchCASBeijing100101China; ^2^ Key laboratory of Ecological Restoration in the Hilly AreaPingdingshan UniversityPingdingshanHe'nan Province467000China

**Keywords:** Carbon storage, coefficient variation, forest type classification, multimethodology, spatial interpolation, uncertain

## Abstract

Carbon (C) storage for all the components, especially dead mass and soil organic carbon, was rarely reported and remained uncertainty in China's forest ecosystems. This study used field‐measured data published between 2004 and 2014 to estimate C storage by three forest type classifications and three spatial interpolations and assessed the uncertainty in C storage resulting from different integrative methods in China's forest ecosystems. The results showed that C storage in China's forest ecosystems ranged from 30.99 to 34.96 Pg C by the six integrative methods. We detected 5.0% variation (coefficient of variation, CV, %) among the six methods, which was influenced mainly by soil C estimates. Soil C density and storage in the 0–100 cm soil layer were estimated to be 136.11–153.16 Mg C·ha^−1^ and 20.63–23.21 Pg C, respectively. Dead mass C density and storage were estimated to be 3.66–5.41 Mg C·ha^−1^ and 0.68–0.82 Pg C, respectively. Mean C storage in China's forest ecosystems estimated by the six integrative methods was 8.557 Pg C (25.8%) for aboveground biomass, 1.950 Pg C (5.9%) for belowground biomass, 0.697 Pg C (2.1%) for dead mass, and 21.958 Pg C (66.2%) for soil organic C in the 0–100 cm soil layer. The R:S ratio was 0.23, and C storage in the soil was 2.1 times greater than in the vegetation. Carbon storage estimates with respect to forest type classification (38 forest subtypes) were closer to the average value than those calculated using the spatial interpolation methods. Variance among different methods and data sources may partially explain the high uncertainty of C storage detected by different studies. This study demonstrates the importance of using multimethodological approaches to estimate C storage accurately in the large‐scale forest ecosystems.

## Introduction

Forest ecosystems contain over 45% of carbon in terrestrial biosphere and thus play a leading role in the globe carbon cycle (Beer et al. 2010). An accurate estimate of ecosystem C storages in forests is crucial for predicting the national carbon‐climate feedback and guiding the implementation of mitigation policies (Beer et al. 2010; McKinley et al. 2011; Pan et al. 2011; Yang et al. 2014). China's forests make up 5% of the globe total and rank as the fifth largest area in the world (Hu et al. 2015). Furthermore, the Chinese government has signed the “United Nations Framework Convention on Climate Change” and the “Kyoto Protocol,” pledging to increase 40 million hectares of forest area by 2020 from the 2005, aiming to noticeably enhance C sequestration by China's forests (Zeng 2014).

Several studies have demonstrated that China's forests act as carbon sink based on the National Forest inventory data (Fang et al. 2001; Pan et al. 2004; Piao et al. 2005; Xu et al. 2007; Zhao et al. 2013). Some studies indicate that China's forests have tremendous potential to increase C storage through afforestation or improved forest management (Deng et al. 2014; Song et al. 2014; Wang et al. 2014). However, many studies only focused on the biomass C storage in China's forests, C storage capacity in other components of forest ecosystem, such as dead mass (DMC) and soil organic carbon (SOC), has been rarely reported (Dixon et al. 1994; Zhou et al. 2000; Ni 2001; Li et al. 2004; Yu et al. 2010). Carbon storage in soils in China was considerably high, accounting for 12.8% of the global C pool, and played an important role in the global carbon cycle (Fang et al. 1996). However, the estimates of C storage in different components, especially SOC in China's forest ecosystems, are still uncertain. The uncertainty in ecosystem C storage estimation limits our understanding on whole‐ecosystem carbon balance in China's forests and its feedback to climate warming (Luyssaert et al. 2010; Pan et al. 2011; Yang et al. 2014).

The uncertainties in ecosystem C storage estimation in China's forest ecosystem are mainly induced by data sources and methods. Many scientists estimated biomass C based on National Forest inventory (NFI) data by biomass expansion factor (BEF) method (Fang et al. 1998, 2001, 2007; Pan et al. 2004; Xu et al. 2007; Zhang et al. 2013). Piao et al. (2005) and Chi (2011) assessed biomass C based on satellite data by remote sensing method. Other scientists estimated biomass C storage using mean carbon density method based on field‐measured data (Dixon et al. 1994; Fang et al. 1998; Zhou et al. 2000; Ni 2001). These different data sources and methodological issues led to large uncertainties in biomass C density estimates (ranging from 32 to 114 Mg C·ha^−1^) and biomass C storage estimates (ranging from 3.26 to 19 Pg C in 1980s). Even using the same NFI data during the period from 2004 to 2008, different methods cause the estimates of biomass C density to range from 37.94 to 50.71 Mg C·ha^−1^ and biomass C storage to range from 6.24 to 9.25 Pg C (Guo et al. 2010; Li et al. 2011; Zhao et al. 2013). Compared with NFI for different time periods, periodic soil surveys have been conducted less frequently. Consequently, contemporary soil carbon measured data are often unavailable (Yang et al. 2014). The lack of sufficient soil organic C measurements has been the largest obstacle to elucidate the current status of soil C storage (Yang et al. 2007; Yu et al. 2010; Pan et al. 2011). Based on historical soil survey data and the global soil data sets in 1980s, soil organic C storage and C density in the 0–100 cm soil layer in China's forests have been estimated to be 16–23.21 Pg C and 115.90–193.55 Mg C·ha^−1^, respectively (Dixon et al. 1994; Zhou et al. 2000; Li et al. 2004; Xie et al. 2004; Yang et al. 2007; Yu et al. 2007). The large variability in soil carbon observed among previous studies may be partly induced by the lack of contemporary measurements of C stock in forest soils (Pan et al. 2011; Yang et al. 2014). Furthermore, dead mass C in China's forests has been very rarely reported (Zhou et al. 2000). Therefore, estimates of C storage remain uncertain in China's forest ecosystems.

Different methods have been used to estimate C storage in China's forest ecosystems, including the mean carbon density method based on field investigation data (Dixon et al. 1994; Fang et al. 1998; Ni 2001; Guo et al. 2010), the BEF method based on NFI data (Fang et al. 2001; Zhao and Zhou 2006; Xu et al. 2007; Zhang et al. 2013), remote sensing (Piao et al. 2005; Chi 2011), modeling (Dixon et al. 1994; Li et al. 2004; Zhao et al. 2013), and spatial interpolation (Du et al. 2010; He et al. 2013; Zhao et al. 2014). The BEF method based on NFI data was widely used for estimation of biomass C in the forests at national scale. However, NFI only investigated the volume of the stand trees, excluding the biomass of shrubs and herbs, dead mass, or soil organic carbon. More recently, studies on forest biomass carbon estimation combining forest inventory data with remote sensing data have gradually proliferated (Piao et al. 2005; Chi 2011; Huang et al. 2013). However, remote sensing is not appropriate to estimate dead mass and soil organic carbon because field‐measured data are scarce. Taken together, insufficient observations and methodological issues greatly inhibit our estimates of all component carbon storage in China's forest ecosystems. Consequently, field‐investigated data have always been used to estimate C storage, especially for soil C and dead mass C, at the national scale by the mean C density method. However, because field‐investigated data were scarce, the data have been integrated according to the forest type (Ni 2001; Li et al. 2004; Xie et al. 2004; Yang et al. 2014), administrative region (Yang et al. 2014), and spatial interpolation (Du et al. 2010; Zhao et al. 2013). Therefore, these different scale‐up methods may have primarily contributed to the inconsistency of results obtained by different studies (Wang et al. 2001, 2014; McKinley et al. 2011; Ni 2013).

To the best of our knowledge, few studies have conducted integrated analyses of C storage including all the elements C density of aboveground biomass (AGC), belowground biomass (BGC), dead mass (DMC), soil organic carbon (SOC), and the associated uncertainties estimated by different integrative methods using field‐measured data at the national scale.

The rapid development of forest C cycling research provides a new opportunity for accurate assessments of C storage in China's forest ecosystems. Here, we collected 3868 field‐measured data including AGC, BGC, DMC, and SOC in China's forest ecosystems from papers published between 2004 and 2014 and used six integrative methods (three forest type classification methods and three spatial interpolation methods) to calculate the C storage of different components in China's forest ecosystems. This work aimed to address two key issues: (1) providing the first estimate of C storage for different components (AGC, BGC, DMC, and SOC) simultaneously in China's forest ecosystems based on different integrative methods at the national scale and (2) evaluating to what extent these integrative methods cause uncertainty in estimates of C storage in China's forest ecosystems.

## Materials and Methods

### Study area and forest classification

**Figure 1:**
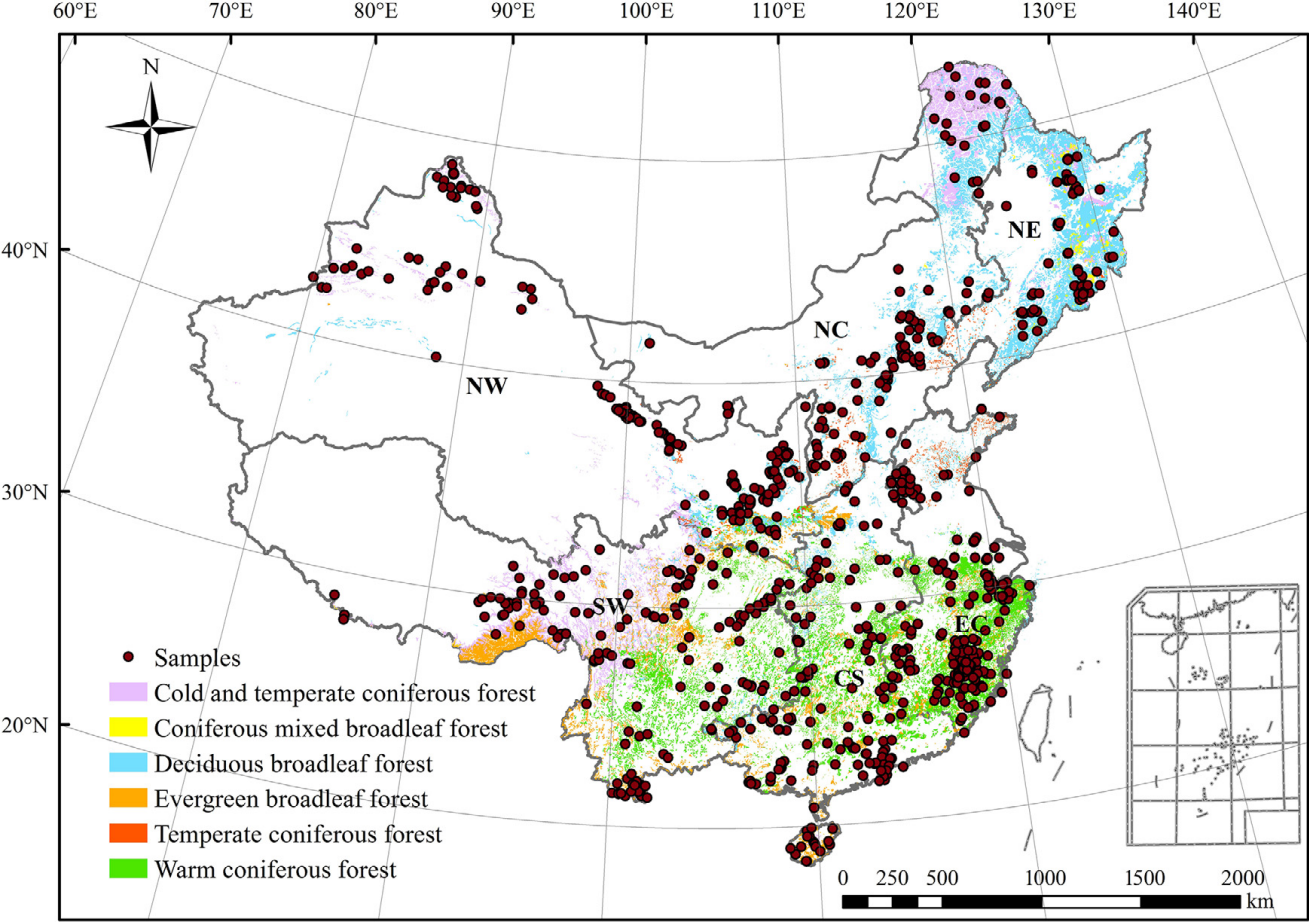
Sampling site locations for carbon density in China's forests from data published between 2004 and 2014. NE: northeast China, NC: northern China, NW: northwest China, SW: southwest China, EC: eastern China, and CS: central southern China.

China covers a broad geographical span and has a large climatic range extending from cold temperate to tropical climate zones from the north to south and arid to humid areas from the northwest to southeast. The broad climatic gradient supports a diversity of forest ecosystems throughout China. Most forests in China are located in the northeast and the southwest regions (Fig. 1). According to the principles and bases of Chinese vegetation regionalization, China's forests are classified into six forest types groups, 16 forest types, and 38 forest subtypes (Hou et al. 1982; Chinese Academy of Sciences 2001; Li et al. 2011) (Appendix S1). Thus, C storage estimates can be scaled up from the 16 forest types or the 38 forest subtypes to the six forest type groups.

### Dataset source

Carbon density data for different components (AGC, BGC, DMC, and SOC) of China's forests were derived from field‐measured data of 485 literatures published from 2004 to 2014 in the Web of Science (http://www.Web of Science knowledge.com) and in the China National Knowledge Infrastructure (http://www.cnki.net/) (Appendix S2). Study sites represented all climatic zones, spanning cold temperate to tropical. The sites ranged from −5.1 to 23.8°C in mean annual temperature (MAT) and 223 to 2515 mm in mean annual precipitation (MAP). Furthermore, we obtained the China's forest areas and the spatial distribution of different forest types from the vegetation map of China (1:1,000,000 scales) (http://www.geodata.cn/). Based on this map, the total area of China's forests was 1.5155 × 10^8^ ha.

### Data collection

To characterize the current state of carbon storage in China's forest ecosystems (excluding Hong Kang, Macao, and Taiwan), we synthesized all the published studies on the available field‐measured data including of biomass, litter fall mass, dead trees mass, SOC, SOM, and C density for different components (AGC, BGC, DMC, SOC) of China's forests from 2004 to 2014. The published literatures were retrieved from the Web of Science (http://www.Web of Science knowledge.com) and the China National Knowledge Infrastructure (http://www.cnki.net/). All the found literatures were further screened by the following specific critical criteria (Luo et al. 2014; Yang et al. 2014).

#### Estimates of trees biomass and C density

Tree‐level biomass (oven‐dried mass) was measured by destructive harvesting and weighing of tree components (e.g., stems, branches, leaves, and roots, kg), and which were subsequently scaled up to the stand level (e.g., stems, branches, leaves, and roots, oven‐dried mass per unit area, Mg·ha^−1^). In particular, the tree‐level root biomass was measured using the full‐excavation method (Luo et al. 2014). Stand‐level tree biomass was determined using plot clear‐cutting (1% of the retrieved papers in this study), average tree (87% of the retrieved papers), and allometry (12% of the retrieved papers) methods. Stand‐level biomass for the AGC and BGC of trees was estimated from the sum of these components based on tree numbers and sample plot dimensions (Luo et al. 2014). However, for allometric equation studies, we only collected reliable stand‐level biomass data that applied unique allometric equations developed specifically for each individual study (Luo et al. 2014). It should be noted that our stand‐level biomass data of trees excluded biomass data estimated using the BEF method and modeling methods. Furthermore, the C contents for stems, branches, leaves, and roots were extracted from data measured by the original studies. When the C content was not directly measured, it was estimated as 45% of dry matter (Levine et al. 1995; Wang et al. 2001). Thus, we used biomass and the coefficient of C content to calculate the C density for each component of the trees at stand level.

#### Estimates of understory vegetation biomass and C density

The aboveground biomass of understory vegetation types (saplings, shrubs, and herbs) was measured using quadrat destructive harvesting methods, while root biomass was measured using full‐excavation methods. Subsequently, stand‐level aboveground biomass or root biomass for each understory types was calculated from the oven‐dried mass of the components and the quadrat area. The C contents of understory vegetations were extracted from the data measured by the original studies. When the C content was not directly measured, it was estimated as 45% of dry matter (Levine et al. 1995; Wang et al. 2001). Thus, we used biomass and the coefficient of C content at stand level to calculate the C density of understory vegetation.

#### Estimates of AGC and BGC

AGC and BGC were calculated using equations (1) and (2).

(1) $$\text{AGC}=\left(B_{S}\times C_{S}+B_{B}\times C_{B}+B_{\mathrm{Br}}\times C_{\mathrm{Br}}+B_{L}\times C_{L}\right)+\left({\text{AGB}}_{\mathrm{Sh}}\times C_{\mathrm{Sh}}+{\mathrm{AGB}}_{H}\times C_{H}\right)$$

where *B*_S_, *B*_B_, *B*_Br_, *B*_L_, AGB_Sh_, and AGB_H_ represent the biomass of the stem, bark, branch, leaf of trees, and AGB of shrubs and herbs, respectively (Mg·ha^−1^). *C*_S_, *C*_B_, *C*_Br_, *C*_L_, *C*_Sh_, and *C*_H_ represent the C content (%) of the stem, bark, branch, leaf of trees, aboveground shrubs and herbs, respectively.

(2) $$\text{BGC}=B_{\mathrm{root}-A}\times C_{\mathrm{root}-A}+B_{\mathrm{root}-\mathrm{Sh}}\times C_{\mathrm{root}-\mathrm{Sh}}+B_{\mathrm{root}-H}\times C_{\mathrm{root}-H}$$

where *B*_root‐A_, *B*_root‐Sh_, and *B*_root‐H_ represent the root biomass of trees, shrubs, and herbs, respectively (Mg C·ha^−1^), and *C*_root‐A_, *C*_root‐Sh_, and *C*_root‐H_ represent the C content (%) of the roots for trees, shrubs, and herbs, respectively.

#### Estimates of dead mass and C density

Dead mass in forests includes litter (dead plant organs, e.g., fine branches, leaves, flowers, seeds, and coarse woody debris) and dead trees (standing dead trees and fallen dead trees) in various stages of decay. The mass of litter was determined using quadrat harvesting methods and was subsequently scaled up to the stand level based on quadrat area. The mass of dead trees was measured using average tree and allometry methods and was subsequently scaled up to the stand level based on dead tree numbers and sample plot dimensions. Total dead mass at the stand level was the dead mass sum of stand‐level litter and dead trees. The C contents of litter and dead trees were extracted from the measured data in the original studies. When the C content was not directly measured, it was estimated as 45% of dry matter (Levine et al. 1995). Thus, we calculated DMC at stand level using equation (3).

(3) $$\text{DMC}=B_{\mathrm{Litter}}\times C_{\mathrm{Litter}}+B_{\mathrm{Dead}\;\mathrm{trees}}\times C_{\mathrm{Dead}\;\mathrm{trees}}$$

where *DMC*,* B*_Litter_, and *B*_Dead trees_ are dead mass C density (Mg·ha^−1^), litter mass (Mg·ha^−1^), and mass of dead trees (Mg·ha^−1^), respectively. *C*_Litter_ and *C*_Dead trees_ represent the C content (%) of the litter and dead trees, respectively.

#### Estimates of SOC density (SOC)

Data on SOC and soil organic matter (SOM) content, soil bulk density, and soil layer depth were extracted from papers published between 2004 and 2014. Our dataset excluded recently disturbed forest plots (e.g., cutting, fire, and fertilizer). Only data from untreated plots was used from manipulation experiments. The SOC value was calculated using the SOM value by a conversion coefficient of 0.58. If soil bulk density data was not directly reported in the published papers, the soil bulk density was calculated using the equation (Paul et al. 2002).

(4) $$\text{BD}=\frac{100}{\left(\%\text{OM}/{\text{BD}}_{\mathrm{OM}}\right)+\left(\left(100-\%\text{OM}\right)/{\text{BD}}_{\min\;\mathrm{soil}}\right)}$$

where %OM is the percent soil organic matter, BD_OM_ is the bulk density of the organic matter (assumed to be 0.244), and BD_min soil_ is the mineral soil bulk density (assumed to be 1.64) (Paul et al. 2002)When the soil sample depth was lower than 100 cm, we used the empirical relationship between soil C content and depth to fit to the 100 cm soil layer. This empirical relationship between soil C content and depth had been established among 74 terrestrial ecosystems in China, using the long‐term monitoring data from the Chinese Ecosystem Research Network (Chai et al. 2015). Here, we randomly selected 445 sites from this dataset to validate the accuracy of the prediction and found that the predicted SOC values were almost perfectly correlated with the measured values in the 0–100 cm soil layer (*y *=* *0.9835*x* + 3.636, *n *=* *445, *R* ^2^ = 0.9774, *P *<* *0.0001, Appendix S7). SOC was calculated using equation (5).

(5) $$\text{SOC}=\sum_{i}^{n}H_{i}\times {\text{BD}}_{i}\times {\text{SOC}}_{i}\times \left(1-C_{i}\right)$$

where SOC, *H*_*i*_, BD_*i*_, SOC_*i*_, and *C*_*i*_ are soil organic carbon density (Mg·ha^−1^), soil depth (cm), bulk density (g·cm^−3^), and percentage of rock fraction >2 mm (%), respectively (Yang et al. 2014).

Overall, the dataset contained 3868 records of C density at the plot scale, including 2452, 2315, 1100, and 1162 detailed records on AGC, BGC, DMC, and SOC, respectively. Furthermore, plot information was also extracted, including latitude, longitude, altitude, MAT, MAP, dominated tree species, forest origin, and stands age. Any missing geographical coordinates were digitized from Google Maps (http://maps.google.com).The spatial distribution of the sampling plots is shown in Figure 1. Detailed information about the collected data and literature sources is provided in the Appendices (Appendices S3–S6).

### Carbon storage estimates using integrative methods

To assess the influence of different integrative methods on C storage estimates in the scaling‐up process in this study, we adapted three forest type classification methods and three spatial interpolation methods (Table 1).

**Table 1:** Description of six integrative methods for C storage estimation in China's forest ecosystems

	No.	Methods	Assumption
Forest type classifications at different scales	M1	Six forest type groups^a^	Forests show different characteristics resulting from climate (temperature and precipitation), topography, soil, and management history (Fang et al. 2012; Reich et al. 2014). Forests therefore can be artificially divided into different types (Chinese Academy of Sciences 2001). On basis of the assumption, C storage in China's forests can be calculated by the forest type classification and corresponding area at different scales
	M2	Sixteen forest types	
	M3	Thirty‐eight forest subtypes	
Spatial interpolation	M4	Kriging interpolation	The geostatistical principle assumes that forest distribution gradually changes with climate, latitude, longitude, and altitude (Fang et al. 2012; Reich et al. 2014); that is, biomass C or soil C storage from one sampling site will be most similar to these of the nearest site. On the basis of this assumption, spatial interpolation methods can be used to estimate forest biomass C or soil C storage in China
	M5	Inverse distance weighted interpolation	
	M6	Empirical Bayesian kriging interpolation	

a Carbon storage estimated by 16 forest types and 38 forest subtypes can be scaled up to C storage of six forest type groups by area‐weighted methods.

#### Carbon storage estimates by forest type classification methods

Forests have horizontal and vertical distribution patterns, which are influenced by climate (temperature and precipitation), topography, soil types, and management history (Fang et al. 2012; Reich et al. 2014). Therefore, forests could be artificially divided into different types at different scales (Hou et al. 1982; Chinese Academy of Sciences 2001). Thus, C storage in China's forest ecosystems could be calculated from the different forest types and corresponding area at different scales (Table 1 and Appendix S1). Consequently, the process of scaling‐up is of significance, presenting a challenge for using site‐scale C storage estimates for large‐scale estimates.

For the statistical and comparative analyses, China's forests were classified at three different scales: six forest type groups, 16 forest types, and 38 forest subtypes based on the regionalization of vegetation in China (Hou et al. 1982; Chinese Academy of Sciences 2001), the vegetation map of China at 1:1,000,000 scale (http://www.geodata.cn/), and the forest type divisions from the technical specifications of the national forest inventory (Li et al. 2011). Then, C storage for the different forest components was analyzed according to six forest type groups (M1), 16 forest types (M2), and 38 forest subtypes (M3), respectively (Appendix S1 and Table 1).

#### Carbon storage estimates by spatial interpolation methods

The geostatistical principle assumes that forest distribution gradually changes with latitude, longitude, and altitude (Fang et al. 2012; Reich et al. 2014); that is, biomass and C storage at one sampling site may have the highest similarity to that at the nearest site. Based on this assumption, spatial interpolation methods have been widely used to estimate forest biomass C and SOC (Malhi et al. 2006; Sales et al. 2007; Rossi et al. 2009; Du et al. 2010; Zhao et al. 2014). In practice, we selected three spatial interpolation methods to estimate C storage in China's forest: Kriging interpolation (M4), inverse distance weighted interpolation (M5), and empirical Bayesian kriging interpolation (M6) (Table 1). Among these methods, M5 only considers the distance, whereas M4 and M6 consider both the spatial orientation and the distance.

For statistical and comparative analyses, China's forests were divided into six geological regions according to China administrative divisions: northeast China (NE), northwest China (NW), northern China (NC), southwest China (SW), eastern China (EC), and central southern China (CS) (Fig. 1).

### Data analysis

The coefficient of variation (CV, %) was used to assess the variance of the six methods for estimating C storage (Oren et al. 2006; Liang et al. 2010; Yu et al. 2011), which was defined as the ratio of the standard deviation to the mean. AGC, BGC, DMC, and SOC in the 0–100 cm soil layer were estimated by the mean C density method based on three forest type classifications and geostatistical methods by the three spatial interpolation methods. Carbon storage estimated from the 16 forest types and 38 forest subtypes was scaled up to C storage of the six vegetation type groups by area‐weighted methods. Spatial interpolation methods were applied using ArcGIS (Version 10.2, ESRI Inc. Redlands, CA). The *t*‐test was used to obtain the 95% confidence level of C storage estimated from six methods (R i386 3.2.1; R Foundation for Statistical Computing, Vienna, Austria).

## Results

### Carbon density and storage estimates by forest classifications methods

**Figure 2:**
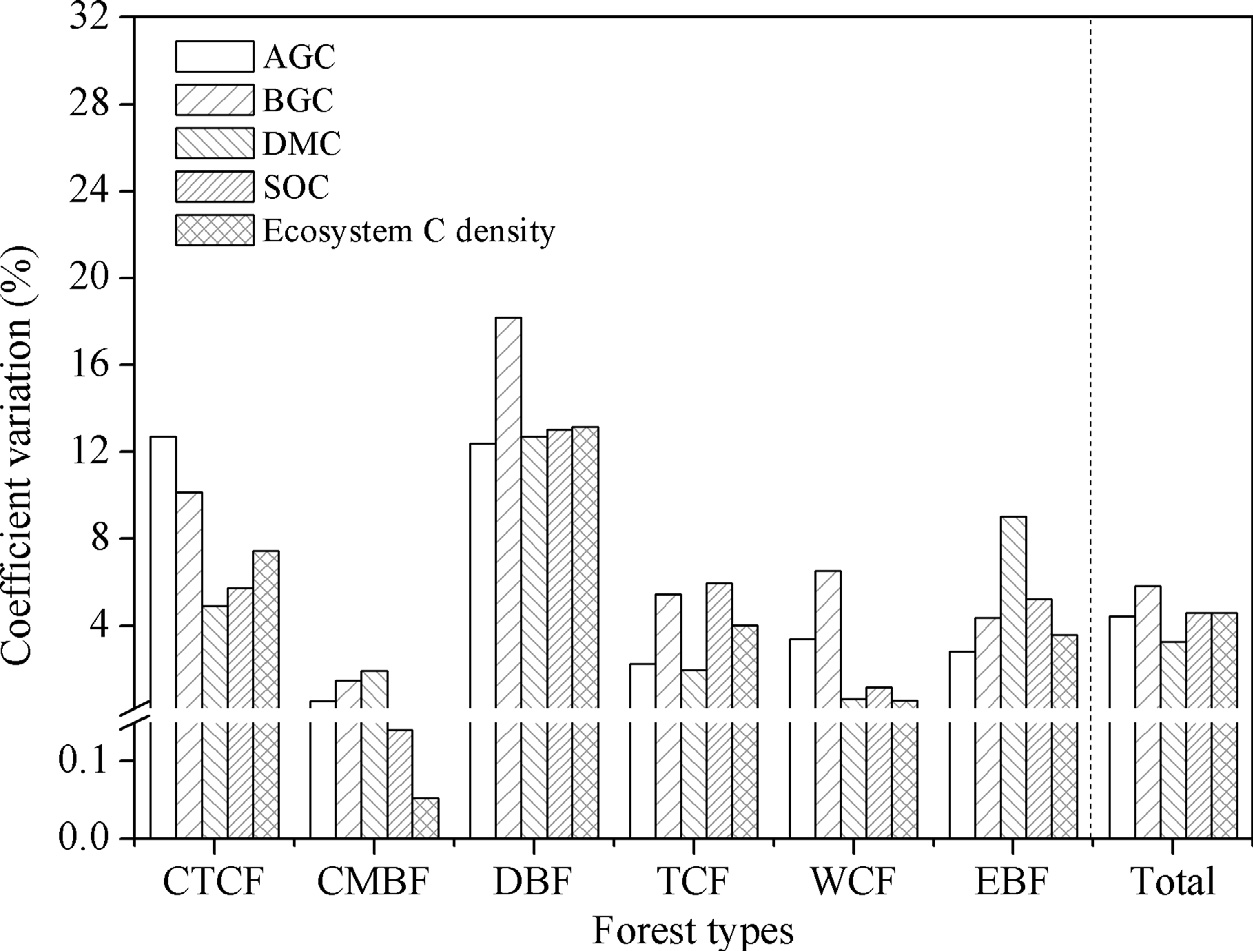
Coefficient of variation (CV %) of carbon density in the different components of China's forest ecosystems. Data were calculated using three vegetation classification methods. AGC, C density in aboveground biomass; BGC, C density in belowground biomass; DMC, C density in dead mass; SOC, soil organic C density in the 0–100 cm soil layer. DMCF, deciduous broadleaf forest; EBF, evergreen broadleaf forest; CMBF, coniferous mixed broadleaf forest; CTCF, cold temperate coniferous forest; TCF, temperate coniferous forest; WCF, warm coniferous forest.

The C density estimated by three forest type classifications varied greatly among different components in the six forest type groups (Table 2, Appendix S8‐S10, and Fig. 2). AGC ranged from 37.04 to 76.91 Mg C·ha^−1^ and was highest in CTCF and lowest in DBF. The highest and lowest CV values for AGC resulting from the three forest type classifications were in CTCF (12.72%) and CMBF (0.54%), respectively. BGC ranged from 9.16 to 16.33 Mg C·ha^−1^, with the highest and lowest CV values for BGC occurring in DBF (18.26%) and CMBF (1.49%), respectively. DMC ranged from 2.63 to 10.05 Mg C·ha^−1^, with the highest and lowest CV values for DMC occurring in DBF (12.70%) and WCF (0.62%), respectively. SOC ranged from 89.33 to 215.83 Mg C·ha^−1^ and was highest in CTCF and lowest in TCF. The highest and lowest CV values for SOC were obtained in DBF (13.03%) and CMBF (0.14%).

**Table 2:** Carbon density and storage in China's forest ecosystems estimated by the three forest type classifications

Six forest type group^a^	Methods^a^	Carbon density (Mg C·ha^−1^)										
			AGC^b^		BGC		DMC		SOC		Ecosystem	
			Mean	SE	Mean	SE	Mean	SE	Mean	SE	Mean	SE
Cold and temperate coniferous forests		M1	59.75	2.01	13.09	0.52	8.76	0.64	194.23	5.13	275.83	5.57
		M2	76.91	3.89	15.67	0.94	7.94	1.08	213.93	11.93	314.45	12.64
		M3	75.20	4.21	15.72	1.06	8.33	1.28	215.83	13.04	315.08	13.81
Coniferous mixed broadleaf forests		M1	70.78	5.17	14.89	1.19	10.05	0.98	189.98	12.06	285.70	13.21
		M2	71.44	6.86	15.28	1.46	9.72	0.93	189.52	12.16	285.96	14.68
		M3	71.44	6.86	15.28	1.46	9.72	0.93	189.52	12.16	285.96	14.68
Deciduous broadleaf forest		M1	38.83	1.38	10.31	0.39	2.63	0.19	116.57	4.67	168.34	4.89
		M2	37.04	1.78	9.87	0.52	2.73	0.23	106.97	5.55	156.61	5.86
		M3	46.56	4.33	13.62	2.62	3.31	0.33	137.63	10.89	201.12	12.52
Temperate coniferous forests		M1	38.59	2.13	10.54	0.86	5.65	0.75	99.19	4.78	153.97	5.36
		M2	38.59	2.13	10.54	0.86	5.65	0.75	99.19	4.78	153.97	5.36
		M3	37.10	3.55	11.57	2.08	5.46	1.26	89.33	6.02	143.46	7.80
Warm coniferous forests		M1	54.05	1.81	10.28	0.32	3.76	0.21	119.93	2.22	188.02	2.89
		M2	54.05	1.81	10.28	0.32	3.76	0.21	119.93	2.22	188.02	2.89
		M3	50.94	3.49	9.16	0.63	3.72	0.39	122.39	6.54	186.21	7.54
Evergreen broadleaf forests		M1	68.27	2.66	15.08	0.65	3.37	0.17	134.69	3.38	221.41	4.35
		M2	67.12	4.82	16.15	1.26	3.63	0.42	149.41	6.91	236.31	8.66
		M3	70.91	5.91	16.33	1.55	4.03	0.52	143.81	8.22	235.08	10.44
National total		M1	52.29	1.89	11.59	0.45	4.48	0.31	136.11	3.92	204.47	4.45
		M2	55.00	2.71	12.13	0.67	4.38	0.44	139.14	6.04	210.65	6.78
		M3	57.15	4.28	13.00	1.51	4.67	0.60	148.75	9.48	223.57	10.84

Six forest type group^c^	Area (10^8 ^ha)	Methods^c^	Carbon storage (Pg C)									
			AGC^b^		BGC		DMC		SOC		Ecosystem	
			Mean	SE	Mean	SE	Mean	SE	Mean	SE	Mean	SE
Cold and temperate coniferous forests	0.3015	M1	1.801	0.061	0.395	0.016	0.264	0.019	5.855	0.155	8.316	3.748
		M2	2.318	0.117	0.472	0.028	0.239	0.033	6.449	0.360	9.480	3.704
		M3	2.267	0.127	0.474	0.032	0.251	0.039	6.507	0.393	9.498	3.622
Coniferous mixed broadleaf forests	0.0191	M1	0.135	0.010	0.028	0.002	0.019	0.002	0.362	0.023	0.544	0.238
		M2	0.136	0.013	0.029	0.003	0.019	0.002	0.361	0.023	0.545	0.232
		M3	0.136	0.013	0.029	0.003	0.019	0.002	0.361	0.023	0.545	0.236
Deciduous broadleaf forest	0.4704	M1	1.826	0.065	0.485	0.018	0.124	0.009	5.483	0.220	7.918	5.719
		M2	1.743	0.084	0.464	0.024	0.128	0.011	5.032	0.261	7.367	5.217
		M3	2.190	0.204	0.641	0.123	0.155	0.016	6.474	0.512	9.460	4.694
Temperate coniferous forests	0.0389	M1	0.150	0.008	0.041	0.003	0.022	0.003	0.386	0.019	0.599	0.382
		M2	0.150	0.008	0.041	0.003	0.022	0.003	0.386	0.019	0.599	0.382
		M3	0.144	0.014	0.045	0.008	0.021	0.005	0.347	0.023	0.558	0.322
Warm coniferous forests	0.4702	M1	2.542	0.085	0.483	0.015	0.177	0.010	5.640	0.104	8.841	4.584
		M2	2.542	0.085	0.483	0.015	0.177	0.010	5.640	0.104	8.841	4.584
		M3	2.395	0.164	0.431	0.030	0.175	0.018	5.755	0.308	8.756	4.289
Evergreen broadleaf forests	0.2154	M1	1.471	0.057	0.325	0.014	0.073	0.004	2.902	0.073	4.770	2.757
		M2	1.446	0.104	0.348	0.027	0.078	0.009	3.219	0.149	5.091	2.757
		M3	1.528	0.127	0.352	0.033	0.087	0.011	3.098	0.177	5.065	2.243
Total	1.5155	M1	7.925	0.286	1.757	0.069	0.678	0.047	20.628	0.593	30.987	17.429
		M2	8.335	0.411	1.839	0.101	0.663	0.067	21.086	0.916	31.923	16.366
		M3	8.660	0.649	1.971	0.229	0.708	0.090	22.542	1.437	33.882	15.405

a M1, C storage was directly estimated by six forest type groups. M2, C storage was estimated by 16 forest types and scaled up to six forest type groups by area‐weighted method. M3, C storage was estimated by 38 forest subtypes and scaled up to six forest type groups by area‐weighted method.

b AGC, aboveground vegetation biomass carbon density; BGC, belowground vegetation biomass carbon density; DMC, dead mass carbon density; SOC, soil organic carbon density in the 0–100 cm soil layer.

c M1, directly estimated C storage by six forest type group. M2, C storage was estimated by 16 forest types and scaled up to six forest type groups by area‐weighted method. M3, C storage was estimated by 38 forest subtypes and scaled up to six forest type groups by area‐weighted method.

Carbon storage differed in the different forest type groups. The greatest C density of the forest ecosystem was in CTCF (275.83–315.08 Mg C·ha^−1^), while the lowest was in TCF (143.46–153.97 Mg C·ha^−1^). Carbon storage was the highest in CTCF (8.32–9.50 Pg C) and lowest in CMBF (0.54–0.55 Pg C) (Table 2). The C density of all the components estimated from 38 forest subtypes was greater than that of 16 forest types and six forest type groups.

At the national scale, the ranges of estimated C density for AGC, BGC, DMC, and SOC were 52.29–57.15, 11.60–13.01, 4.38–4.67, and 136.11–148.75 Mg C·ha^−1^, respectively. The CV values resulting from three forests type classifications were greatest in BGC, followed by SOC and AGC (Fig. 2). Overall, the total C storage in China's forest ecosystems (including AGC, BGC, DMC, and SOC in the 0–100 cm soil layer) was 30.99–33.88 Pg C with a mean CV value of 4.58% estimated by the three forest type classification methods.

### Carbon density and storage estimates using spatial interpolation methods

**Figure 3:**
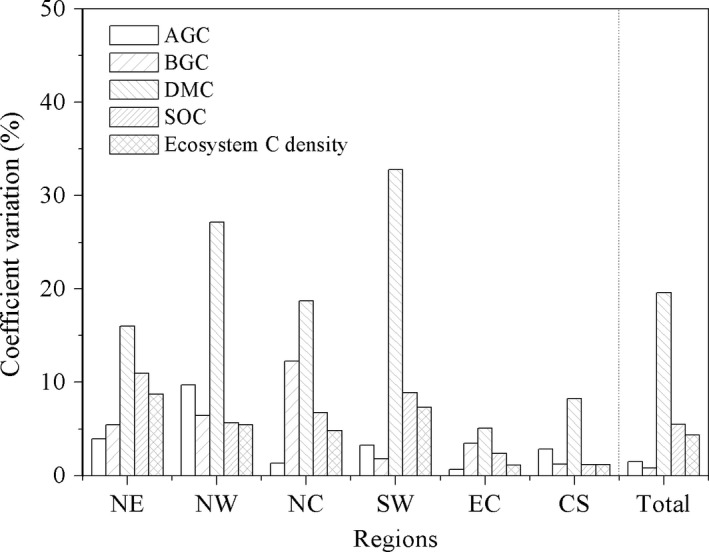
Coefficient of variation (CV %) of carbon density in the different components of China's forest ecosystems. Data were calculated from three spatial interpolation methods. AGC, C density in aboveground biomass; BGC, C density in belowground biomass; DMC, C density in dead mass; SOC, soil organic C density in the 0–100 cm soil layer. NE: northeast China, NW: northwest China, NC: northern China, SW: southwest China, EC: eastern China, and CS: central southern China.

**Figure 4:**
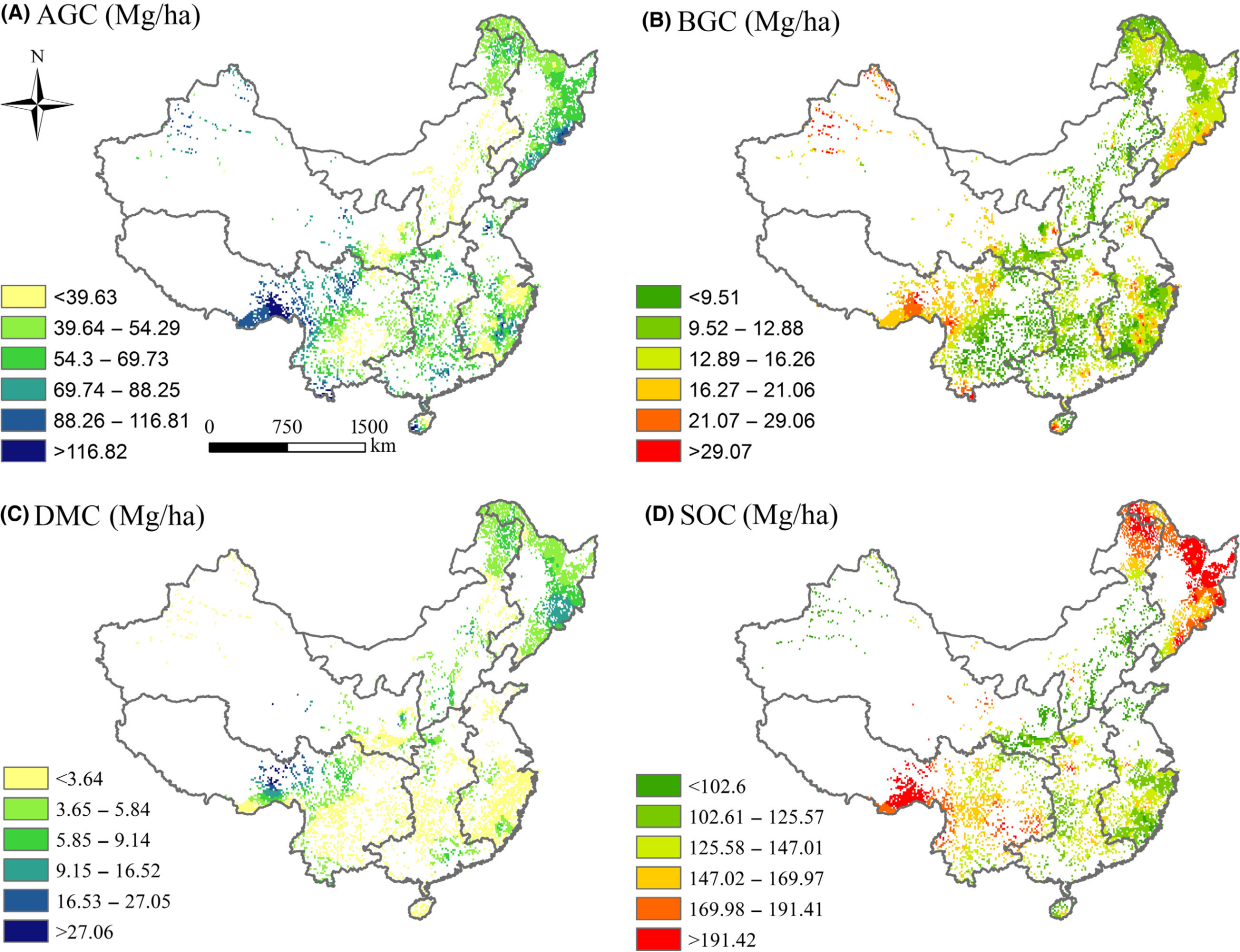
Spatial distribution of carbon density (Mg C·ha^−1^) in AGC (A), BGC (B), DMC (C), and SOC (D) in China's forest ecosystems. The data were averaged from three spatial interpolation methods. AGC, C density in aboveground biomass; BGC, C density in belowground biomass; DMC, C density in dead mass; SOC, soil organic C density in the 0–100 cm soil layer.

The C density estimated by three spatial interpolation methods differed among different components in the six regions (Table 3 and Fig. 3). AGC ranged from 42.05 to 75.44 Mg C·ha^−1^, with the highest and lowest CV values for AGC resulting from the three spatial interpolations occurring in NW (9.73%) and NC (0.66%), respectively. BGC ranged from 10.5 to 16.64 Mg C·ha^−1^, with the highest and lowest CV values for BGC being in NC (12.28%) and CS (1.24%), respectively. DMC ranged from 2.68 to 7.23 Mg C·ha^−1^, with the highest mean CV value of 19.61% resulting from estimates by three interpolations, while the highest and lowest CV values for DMC were in NW (32.77%) and EC (5.08%), respectively. SOC ranged from 101.74 to 199.92 Mg C·ha^−1^, with the highest and lowest CV values for SOC occurring in NE (10.98%) and CS (1.18%), respectively. The C density of AGC and BGC increased with decreasing latitude (from the north to the south) and was the lowest in NC and highest in SW. In contrast, The C density of DMC and SOC increased with increasing latitude (from the south to the north), with the greatest values occurring in NE and SW, while lowest values occurring in NW (Fig. 4).

**Table 3:** Regional and national carbon density and storage was estimated by three spatial interpolation methods in China's forest ecosystems

Region	Area (10^8 ^ha)	Methods	Carbon density (Mg C·ha^−1^)					Carbon storage (Pg C)				
			AGC^a^	BGC	DMC	SOC	Ecosystem	AGC	BGC	DMC	SOC	Ecosystem
NE^b^	0.3246	M4^c^	55.51 ± 0.25	14.18 ± 0.06	5.04 ± 0.05	160.40 ± 0.42	231.22 ± 0.49	1.672 ± 0.008	0.460 ± 0.002	0.164 ± 0.002	5.207 ± 0.013	7.502 ± 0.016
		M5	54.01 ± 0.39	12.87 ± 0.09	6.67 ± 0.07	194.70 ± 0.85	268.25 ± 0.94	1.753 ± 0.013	0.418 ± 0.003	0.217 ± 0.002	6.320 ± 0.027	8.707 ± 0.030
		M6	55.69 ± 0.39	12.98 ± 0.08	6.83 ± 0.06	195.92 ± 0.73	271.42 ± 0.84	1.808 ± 0.013	0.421 ± 0.003	0.222 ± 0.002	6.359 ± 0.024	8.810 ± 0.027
NW	0.1188	M4	68.44 ± 0.82	14.82 ± 0.21	2.70 ± 0.04	113.04 ± 0.66	199.00 ± 1.08	0.813 ± 0.010	0.176 ± 0.002	0.032 ± 0.001	1.343 ± 0.008	2.364 ± 0.013
		M5	58.10 ± 1.22	16.64 ± 0.40	3.36 ± 0.15	103.88 ± 1.59	181.98 ± 2.05	0.690 ± 0.014	0.198 ± 0.005	0.040 ± 0.002	1.234 ± 0.019	2.162 ± 0.024
		M6	58.05 ± 1.21	16.58 ± 0.41	4.59 ± 0.41	101.74 ± 1.51	180.96 ± 2.02	0.689 ± 0.014	0.197 ± 0.005	0.055 ± 0.005	1.209 ± 0.018	2.150 ± 0.024
NC	0.1913	M4	42.05 ± 0.31	12.91 ± 0.06	4.08 ± 0.04	136.32 ± 0.85	195.36 ± 0.91	0.804 ± 0.006	0.247 ± 0.001	0.078 ± 0.001	2.606 ± 0.016	3.736 ± 0.017
		M5	43.18 ± 0.53	10.50 ± 0.12	5.74 ± 0.08	154.50 ± 1.69	213.92 ± 1.78	0.826 ± 0.010	0.201 ± 0.002	0.110 ± 0.001	2.956 ± 0.032	4.092 ± 0.034
		M6	42.45 ± 0.44	10.50 ± 0.10	5.80 ± 0.07	152.07 ± 1.58	210.82 ± 1.64	0.812 ± 0.008	0.201 ± 0.002	0.111 ± 0.001	2.909 ± 0.030	4.033 ± 0.031
SW	0.3768	M4	70.97 ± 0.70	14.57 ± 0.13	3.61 ± 0.05	146.01 ± 0.32	235.16 ± 0.78	2.674 ± 0.026	0.549 ± 0.005	0.136 ± 0.002	5.502 ± 0.012	8.861 ± 0.029
		M5	75.44 ± 0.95	15.10 ± 0.19	6.14 ± 0.18	169.09 ± 1.02	265.77 ± 1.41	2.843 ± 0.036	0.569 ± 0.007	0.231 ± 0.007	6.371 ± 0.038	10.014 ± 0.053
		M6	74.65 ± 0.93	14.81 ± 0.17	7.23 ± 0.34	172.55 ± 1.01	269.24 ± 1.43	2.813 ± 0.035	0.558 ± 0.007	0.272 ± 0.013	6.502 ± 0.038	10.145 ± 0.054
EC	0.2887	M4	50.39 ± 0.42	12.93 ± 0.10	2.68 ± 0.02	125.12 ± 0.37	191.12 ± 0.57	1.455 ± 0.012	0.373 ± 0.003	0.077 ± 0.001	3.612 ± 0.011	5.518 ± 0.017
		M5	51.01 ± 0.86	13.52 ± 0.18	2.89 ± 0.04	120.65 ± 0.75	188.07 ± 1.16	1.473 ± 0.025	0.390 ± 0.005	0.083 ± 0.001	3.483 ± 0.022	5.430 ± 0.033
		M6	50.48 ± 0.67	13.85 ± 0.13	2.95 ± 0.04	119.73 ± 0.54	187.01 ± 0.87	1.457 ± 0.019	0.400 ± 0.004	0.085 ± 0.001	3.457 ± 0.016	5.399 ± 0.025
CS	0.2153	M4	57.51 ± 0.39	12.06 ± 0.10	3.11 ± 0.04	130.14 ± 0.40	202.82 ± 0.57	1.238 ± 0.008	0.260 ± 0.002	0.067 ± 0.001	2.802 ± 0.009	4.367 ± 0.012
		M5	60.08 ± 0.87	11.95 ± 0.19	3.65 ± 0.06	132.00 ± 0.93	207.68 ± 1.28	1.294 ± 0.019	0.257 ± 0.004	0.079 ± 0.001	2.842 ± 0.020	4.471 ± 0.028
		M6	60.65 ± 0.55	12.25 ± 0.13	3.51 ± 0.06	128.96 ± 0.66	205.37 ± 0.87	1.306 ± 0.012	0.264 ± 0.003	0.076 ± 0.001	2.766 ± 0.014	4.422 ± 0.019
Total	1.5155	M4	57.12 ± 0.47	13.63 ± 0.10	3.66 ± 0.04	139.04 ± 0.46	213.44 ± 0.67	8.657 ± 0.07	2.065 ± 0.015	0.554 ± 0.006	21.071 ± 0.070	32.347 ± 0.100
		M5	58.58 ± 0.77	13.42 ± 0.17	5.01 ± 0.10	153.12 ± 1.05	230.13 ± 1.32	8.878 ± 0.117	2.033 ± 0.026	0.760 ± 0.015	23.206 ± 0.159	34.876 ± 0.200
		M6	58.63 ± 0.67	13.46 ± 0.15	5.41 ± 0.15	153.16 ± 0.92	230.67 ± 1.16	8.885 ± 0.102	2.041 ± 0.023	0.820 ± 0.023	23.212 ± 0.139	34.958 ± 0.175

a AGC, aboveground vegetation biomass carbon density; BGC, belowground vegetation biomass carbon density; DMC, dead mass carbon density; SOC, soil organic carbon density in the 0–100 cm soil layer.

b NE, northeast China, NW, northwest China, NC, northern China, SW, southwest China, EC, eastern China, and CS, central southern China (See Fig. 1).

c M4, Kriging interpolation, M5, Inverse distance weighted interpolation, M6, Empirical Bayesian kriging interpolation (See Table 1). Data in the brackets represent Mean ± SE.

In general, AGC, BGC, DMC, and SOC at the national scales ranged from 57.12 to 58.63, 13.41 to 13.63, 3.66 to 5.41, and 139.04 to 153.17 Mg C·ha^−1^, respectively. Total C storage ranged from 32.35 to 34.96 Pg C with a mean CV value of 4.36% based on three spatial interpolation methods.

### Carbon storage in China's forest ecosystems and its uncertainty

**Figure 5:**
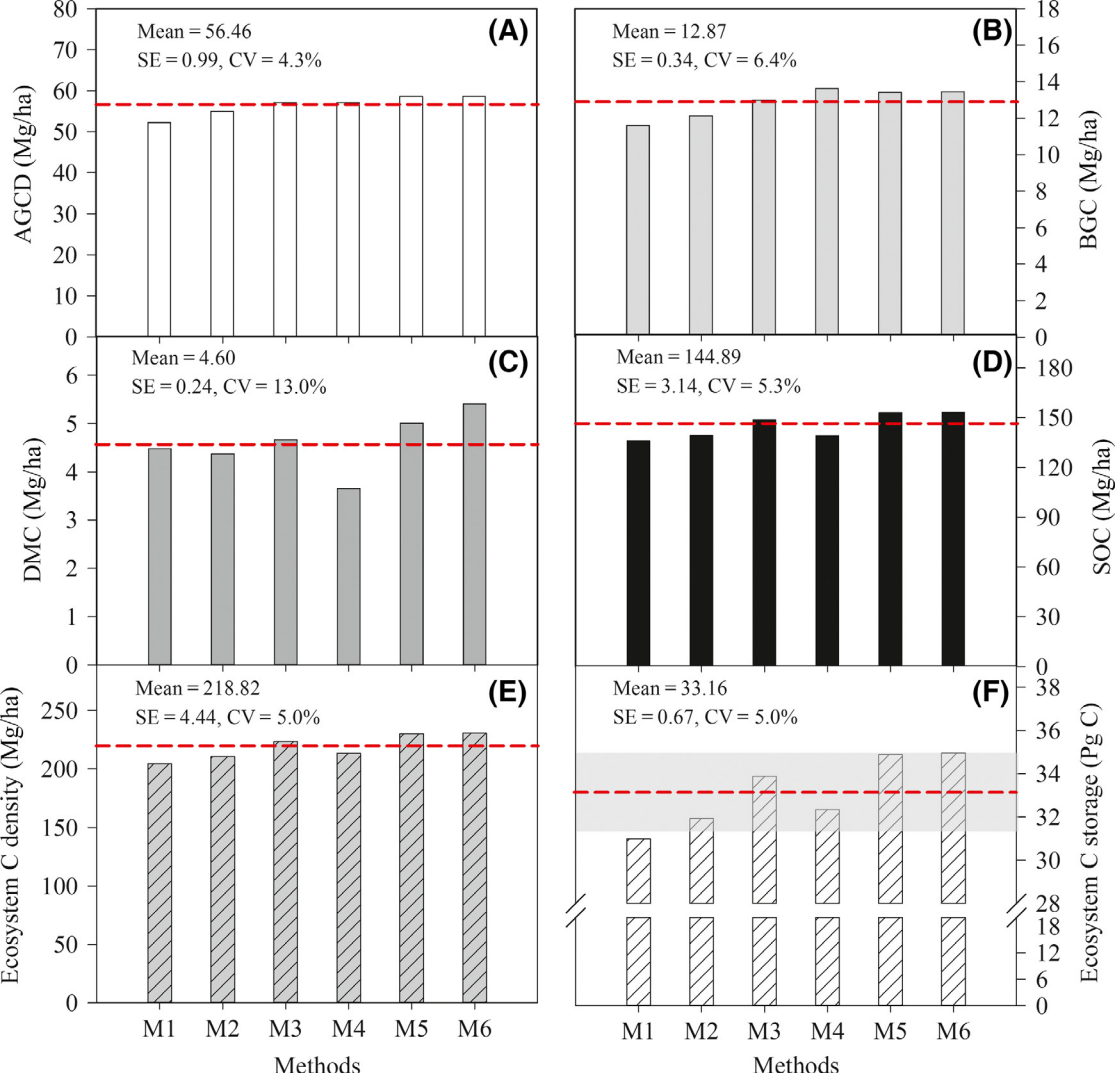
Estimation of carbon density and storage based on the six integrative methods. See Table 1 for a description of the methods. (A) aboveground biomass C density (AGC), (B) belowground biomass C density (BGC), (C) dead mass C density (DMC), (D) soil organic C density in the 0–100 cm soil layer (SOC), (E) ecosystem C density, and (F) ecosystem C storage. In panel (F), the red line indicates mean C storage, and the rectangular area is the variation range at the 95% confidence level estimated by *t‐*test.

The C density estimates for different components in China's forest ecosystems based on six integrative methods were shown in Figure 5. AGC, BGC, DMC, SOC, and total C density ranged from 52.29 to 58.63, 11.59 to 13.47, 3.66 to 5.41, 136.11 to 153.16, and 204.47 to 230.67 Mg C·ha^−1^, respectively (Fig. 5). Soil organic C density showed the greatest variation (17.06 Mg C·ha^−1^) among all C components based on six integrative methods.

When combining all of the forest areas, C storage in China's forest ecosystems (including AGC, BGC, DMC, and SOC in the 0–100 cm soil layer) ranged from 30.99 to 34.96 Pg C, with a mean value of 33.16 Pg C (95% confidence interval of 31.43–34.89 Pg C), and CV value of 5.0% estimated by six integrative methods. In general, C storage estimated by three spatial interpolation methods was greater than that estimated by three forest type classification methods. The estimate of M3, which classified forests into 38 types (33.88 Pg C), was closest to the average of the C storage estimated by six integrative methods (33.16 Pg C).

Total C storage in China's forest ecosystems was composed of 25.8% AGC (8.557 Pg C), 5.9% BGC (1.950 Pg C), 2.1% DMC (0.697 Pg C), and 66.2% SOC (21.958 Pg C). The R:S ratio was 0.23, while C storage of SOC was 2.1 times higher than that of biomass C (AGC + BGC).

## Discussion

### Influence of integrative methods on forest C storage estimates

This study provided the first assessment of the C density and C storage in all of the main components of China's forest ecosystems at the national scale using six integrative methods. We detected 5.0% variation in of C storage estimation of China's forest ecosystems when using the six integrative methods. However, the level of uncertainty appeared to vary among the different components of the forest ecosystem, with the highest and lowest variation being obtained for DMC and AGC, respectively. One reason for the high variation of DMC was caused by three spatial interpolation methods. One possible explanation is that large variation in the DMC data (ranging from 0.01 to 58.70 Mg C·ha^−1^) may have a greater influence on the spatial interpolation methods (Yu et al. 2011). However, this highest variation had little effect on the C storage estimates of forest ecosystems because DMC only accounted for 2.1% of total C storage. In contrast, although the variation in SOC estimates was 5.3% based on the six integrative methods, SOC estimates played an important role in estimating forest ecosystem C accurately because it accounted for 66.2% of C storage in China's forest ecosystems.

Based on six integrative methods, the estimates of C density of SOC in the 0–100 cm soil layer were 136.11–153.17 Mg C·ha^−1^, which was similar to the estimated data reported by previous studies (136.0–157.51 Mg C·ha^−1^) in China's forest ecosystems (Dixon et al. 1994; Ni 2001; Yang et al. 2007; Yu et al. 2007). The C density of forest vegetation was estimated to be 63.89–72.09 Mg C·ha^−1^, which is similar to that obtained by previous studies (67.64–71.73 Mg C·ha^−1^; Fang et al. 1998; Ni 2001) based on the mean C density method. The aboveground biomass C density ranged from 52.29 to 58.63 Mg C·ha^−1^ with an average of 56.46 Mg C·ha^−1^, which compared well with previous estimates (56.75 Mg C·ha^−1^) based on GLAS and MODIS remote sensing method reported by Chi (2011). However, it remains impossible to estimate soil C storage in China's forest ecosystems accurately by remote sensing methods. To maintain the consistency of methods used to estimate the C density of AGC, BGC, DMC, and SOC, this study used the mean C density method and spatial interpolation method to estimate forest ecosystem C storage in China, although the linkage between field investigation and remote sensing should improve estimate of AGC to some extent.

Our results demonstrated that the spatial interpolation methods generally produced higher estimates (224.75 Mg C·ha^−1^) of forest ecosystem C density than that of the forest type classification methods (212.90 Mg C·ha^−1^). One explanation for this difference is that the spatial interpolation methods may be affected more by the number of sampled sites and nearby sites (Yu et al. 2011), particularly those with higher C density (Malhi et al. 2006; Sales et al. 2007). However, SOC estimates based on inverse distance weighted interpolation (M5) and empirical Bayesian kriging interpolation (M6) methods produced similar results estimated by 38 forest subtype classification method (M3). Rossi et al. (2009) indicated that that SOC estimates based on spatial interpolation methods produced similar results to the vegetation type method. For all methods, estimates of China's forest ecosystems C storage using 38 forest subtypes produced the closest value to the mean of the six methods. Theoretically, C storage estimates should be more accurate with the more refined the vegetation classification (Luyssaert et al. 2010; Ni 2013). These findings demonstrate that multiple approaches, especially the multiple‐scale integrative method, should be used to estimate the C storage in large‐scale forest ecosystems.

### Carbon storage estimated in Chinese forest ecosystems

Carbon storage in China's forest ecosystems was estimated to be 30.99–34.96 Pg C based on the six integrative methods, with the AGC, BGC, DMC, and SOC components, representing 7.93–8.89, 1.76–2.07, 0.68–0.82, and 20.63–23.21 Pg C, respectively. This study is the first to simultaneously evaluate C storage in the different components of forest ecosystems in China.

Difference and limitation existed in previous studies estimated C storage at the national scale in China's forest ecosystems (Table 4). One reason for these difference and limitation was the use of the different data sources and the different methods (Baccini et al. 2012; Ni 2013; Wang et al. 2014). Previous studies mainly used NFI data (Fang et al. 2001; Xu et al. 2007; Guo et al. 2010; Li et al. 2011; Zhang et al. 2013), global mean vegetation C (Dixon et al. 1994; Li et al. 2004; Ni 2001), field‐measured data (Fang et al. 1998; Zhou et al. 2000; Ni 2001), and remote sensing data (Piao et al. 2005; Chi 2011) to estimate the biomass C density in China's forests, respectively. Consequently, these estimates varied from 40.14 to 114.0 Mg C·ha^−1^. The use of different data sources may lead to different estimates of C storage. Piao et al. (2005) estimated China's forest biomass C to be 45.31 Mg C·ha^−1^ based on NFI data and satellite data from 1981 to 1999. Chi (2011) estimated AGC of China's forests to be 56.75 Mg C·ha^−1^ using GLAS and MODIS data and field‐measured data from 2006 to 2010 based on remote sensing method, with this value being similar to our result of AGC (56.46 Mg C·ha^−1^). Even when using NFI data at the same time, BEF method with different model parameters and different forest type classification generate different biomass C estimates, ranging from 6.24 to 7.81 Pg C (Guo et al. 2010; Li et al. 2011; Zhang et al. 2013). At present, there are few studies about BGC in China's forest. Previous studies used R:S ratio as a good parameter to infer BGB at the regional scale. However, this ratio also generates uncertainty, due to differences in forest types and regions (Luo et al. 2012). Our study indicated that R:S ratio in China's forests at national scales was 0.23, which is similar to the ratio (0.233) reported by Luo et al. (2012).

**Table 4:** Carbon storage of forest ecosystems in China estimated by different studies

No.	Data source	Methods^2^	Year (a)	Forest area (10^8 ^ha)	Vegetation		Dead mass		1 m Soil organic carbon		Total C storage (Pg C)	Reference source
					C density (Mg C·ha^−1^)	C storage (Pg C)	C density (Mg C·ha^−1^)	C storage (Pg C)	C density (Mg C·ha^−1^)	C storage (Pg C)		
1	NFI^1^	CBM	1984–1988	1.020	39.70	4.06						Liu et al. (2000)
2	NFI	CBM	1984–1988	1.020	43.53	4.45						Fang et al. (2001)
3	NFI	MRM	1984–1988	1.020	37.25	3.80						Fang et al. (1998)
4	NFI	MRM	1984–1988	1.020	32.00	3.26						Wang et al. (2001)
5	NFI	CBM	1984–1988	1.020	36.08	3.69						Pan et al. (2004)
6	NFI	CBM	1989–1993	1.086	38.70	4.20						Liu et al. (2000)
7	NFI	CBM	1989–1993	1.086	42.58	4.63						Fang et al. (2001)
8	NFI	CBM	1989–1993	1.086	37.00	4.02						Pan et al. (2004)
9	NFI	CBM	1989–1993	1.086	41.32	3.78						Zhao and Zhou (2006)
10	NFI	CBM	1989–1993	1.086	37.87	4.11						Xu et al. (2007)
11	NFI	CBM	1994–1998	1.058	44.91	4.75						Fang et al. (2001)
12	NFI	CBM	1994–1998	1.292	36.04	4.66						Xu et al. (2007)
13	NFI	MRM	1981–2000	1.428	52.30	7.46						Fang et al. (2007)
14	NFI	MRM	1999–2003	1.428	38.94	5.56						Wu et al. (2008)
15	NFI	CBM	1999–2003	1.428	41.00	5.85						Fang et al. (2007)
16	NFI	CBM	2004–2008	1.824	42.82	7.81						Li et al. (2011)
17	NFI	CBM	2004–2008	1.555	40.14	6.24						Zhang et al. (2013)
18	NFI	CBM	2004–2008	1.810	37.94	6.87						Guo et al. (2010)
19	NFI	HASM model	2004–2008	1.824	50.71	9.25						Zhao et al. (2013)
20	NFI and satellite data	Remote sense model	1981–1999	1.280	45.31	5.79						Piao et al. (2005)
21	GlAS and MODIS data, and field‐measured data of aboveground biomass	Remote sense model	2006–2010		56.75^a^							Chi (2011)
22	Eight‐km global vegetation map	CEVSA model	1981–1999	1.216	54.72	8.72			190.87	23.21	31.93	Li et al. (2004)
23	758 sites field‐measured data	MCM	1984–1989	1.020	67.64	6.90						Fang et al. (1998)
24	Global dataset from references	Model and MCM	1987–1990	1.180	114.00	17.00			136.00	16.00	33.00	Dixon et al. (1994)
25	1248 sites field‐measured data (including shrubs and herbs)	MCM	1989–1993	1.270	71.73	9.11						Ni (2001)
26	Second national soil survey	MCM	1979–1985	1.493					115.90	17.30		Xie et al. (2004)
27	720 sites field‐measured data	MCM	1989–1993	1.086	57.07	6.20	8.21	0.89	193.55	21.02	28.12	Zhou et al. (2000)
28	Global vegetation and soil database	MCM	1980s	1.428	52.30	7.47			156.20	22.31	29.77	Ni (2001)
29	Second national soil survey	MCM	1979–1985	1.314					157.51	20.70		Yang et al. (2007)
30	1:1,000,000 soil map database of China	MCM	1980s	1.500					143.30	21.50		Yu et al. (2007)
31	3869 sites field‐measured data (including shrubs and herbs)	MCM and Spatial interpolation	2004–2014	1.5155	69.33	10.507	4.60	0.697	144.89	21.958	33.162	This study

NFI, National forest inventory data in China, which provided the volume data of trees with five aged groups at the province level.

CBM, BEF–volume relationship as the continuous BEF method; MRM–estimating forest biomass with a mean BEF value as the mean ratio method; MCM, mean carbon density method.

a 56.75 Mg·ha^−1^ is aboveground biomass C density (AGC) transferred from 126.12 Mg·ha^−1^ of aboveground biomass density by C content of 45% in China's forests.

Compared with biomass C estimated in China's forests, the studies about the DMC and SOC were much fewer. Carbon density of dead mass in China's forest (8.21 Mg C·ha^−1^) was only reported by Zhou et al. (2000), which was higher than the value (4.60 Mg C·ha^−1^) estimated in our study. The reason may be that Zhou et al. (2000) only used 720 field sampling sites, lower than 1100 sampling sites in our study. Soil C is the key C components in forest ecosystem and has had greater uncertainty in China's forests (Fang et al. 2007; Yu et al. 2010; Yang et al. 2014). Previous studies often used the secondary National Soil Survey data in 1980s and Global Dataset to estimate SOC at the national scales (Dixon et al. 1994; Ni 2001; Li et al. 2004; Yang et al. 2007; Yu et al. 2007). Different data sources and different vegetation classifications resulted in different estimated SOC, ranging from 115.90 to 193.55 Mg C·ha^−1^ (Table 4). Furthermore, different soil depths were used in the different estimations, which made these estimates incomparable with other studies. Thus, it was important to overcome these limitations to estimate soil C and its distribution with accuracy (Yang et al. 2007). In our study, SOC down to 1 m soil depth accounted for 66.2% C storage in China's forest ecosystems. Soil organic C density in the 0–100 cm soil layer was estimated to be 136.11–153.17 Mg C·ha^−1^ in China's forest ecosystems based on six integrative methods, which was similar to the results (136.00–157.51 Mg C·ha^−1^) reported in previous studies (Dixon et al. 1994; Ni 2001; Yang et al. 2007; Yu et al. 2007).

Another reason for difference in C density estimates was that field observations for BGC, DMC and SOC were insufficient in forest ecosystems (Pan et al. 2011; Ni 2013). Because of the lack of field investigation on soil C, the proportion of soil C storage in total forest ecosystem has been roughly estimated as 10% in global forests (Pan et al. 2011) and 30% in European forests (Luyssaert et al. 2010). In China, Forest biomass C storage was furtherly estimated using NFI data during different periods (Fang et al. 2001; Xu et al. 2007; Guo et al. 2010; Li et al. 2011; Zhang et al. 2013). However, DMC and SOC were rarely studied in China's forest ecosystems. Compared with NFI data across various periods, periodic surveys of soil in China's forests had not been conducted, which limits the estimates of soil C and results in great uncertainty on the C storage in China's forest ecosystems (Xie et al. 2004; Fang et al. 2007; Yang et al. 2007, 2014). Thus, more soil surveys and sampling sites measuring SOC are required in forest ecosystems (Yu et al. 2007; Rossi et al. 2009; Yang et al. 2014). Our study also supported that simultaneous field observations of C storage in AGC, BGC, DMC, and SOC in China's forest ecosystems should be strengthened in the future.

In addition, different forest type classifications may produce different estimates of C storage because of changes in C density and forest type areas (Luyssaert et al. 2010; Ni 2013). In our study, three forest classifications (six forest type groups, 16 forest types, and 38 forest subtypes) at coarse, median, and fine scales caused estimates of AGC to range from 52.29 to 57.15 Mg C·ha^−1^, while that of BGC ranged from 11.59 to 13.00 Mg C·ha^−1^, and that of SOC ranged from 136.11 to 148.75 Mg C·ha^−1^. Ni (2013) and Zhang et al. (2013) indicated that C storage changes with changes of vegetation classification schemes. Theoretically, C storage estimates should be more accurate with the more refined the vegetation classification (Luyssaert et al. 2010; Ni 2013). In our study, the estimate of C storage based on 38 forest subtypes, which covered most of the dominant tree species in China's forests, was the closest to the mean value at the national scale.

Our findings provide the first evidence demonstrating that integrative methods have an important influence on estimates of forest C storage at the national scale. Yet, previous studies only used one method for the estimates of C storage in forest ecosystems. Thus, we suggest that multiple approaches should be used to improve the accuracy of C storage in forest ecosystems.

## Conclusions

Variation between different integrative methods has noticeable impacts on the accuracy of C storage estimates in forest ecosystems at national scales. Variation in total C storage estimates of China's forest ecosystems was approximately 5.0% when using six integrative methods in this study. The level of uncertainty differed among different forest C components, with the highest values being obtained for DMC and SOC. Carbon storage in China's forest ecosystems combined was estimated to be 30.99–34.96 Pg C, based on the six integrative methods, with the AGC, BGC, DMC, and SOC components representing 7.93–8.89, 1.76–2.07, 0.68–0.82, and 20.63–23.21 Pg C, respectively. Among the three forest classification methods, the C storage estimate based on the 38 forest subtype classification (M3) was closer to the mean value at the national scale. Similarly, the accuracy of C storage estimates by Kling interpolation (M4) was closer to mean value than inverse distance weighted interpolation (M5) and empirical Bayesian kriging interpolation (M6) methods. In general, C storage estimates obtained from the three spatial interpolation methods tended to be higher than those obtained from the forest type classification methods. Overall, the 38 forest subtype classification scheme at the national scale (M3) generated the closest data to mean estimated C storage value for China's forest ecosystems. The findings of this study demonstrate that the underlying influences of integrative methods should be emphasized in future studies. In conclusion, to our knowledge, this work presents the first assessment of C storage in relation to the various components of China's forest ecosystem at the national scale, which may help toward understanding the potential roles of Chinese forests in responding to global climate warming.

## Conflict of Interest

None declared.

## Supporting information


**Appendix S1.** China's forest classification at the different scales was based on the Vegetation Map of China at 1:1000000 scales^†^.
**Appendix S2.** Data used in this study collected from the published literature during the period from 2004 to 2014.

**Appendix S3.** Site information and carbon density of above ground biomass (AGC) from 259 studies published between 2004 and 2014 (See dataset).
**Appendix S4.** Site information and carbon density of below ground biomass (BGC) from 259 studies published between 2004 and 2014 (See dataset).
**Appendix S5.** Site information and the related carbon density of dead mass (DMC) from 180 studies published between 2004 and 2014 (See dataset).
**Appendix S6.** Site information and the related soil organic carbon density in the 0‐100 cm soil layer (SOC) from 187 studies published between 2004 and 2014 (See dataset).
**Appendix S7.** The relationships were between the measured and predicted values of SOC density (SOCD, Mg/ha) in the 0–100 cm soil layer.
**Appendix S8.** Carbon density and storage were estimated by 6 forest type groups in China's forest ecosystems.
**Appendix S9.** Carbon density and storage were estimated by 16 forest types in China's forest ecosystems.
**Appendix S10.** Carbon density and storage were estimated by 38 forest subtypes in China's forest ecosystems.
