# Supplementary material for: Carbon storage in China's forest ecosystems: estimation by different integrative methods
Source: Ecol Evol. 2016 Apr 3;6(10):3129–45. doi: 10.1002/ece3.2114 (PMC4823146; doi:10.1002/ece3.2114)
Supplement: Supplementary file 1 — Appendix S1. China's forest classification at the different scales was based on the Vegetation Map of China at 1:1000000 scales†. Appendix S2. Data used in this study collected from the published literature during the period from 2004 to 2014. [file ECE3-6-3129-s001.doc]

**Carbon storage in China’s forest ecosystems: estimation by different integrative methods**

Shunlei Peng^1,2^, Ding Wen^1^, Nianpeng He^1*^, Guirui Yu^1*^, Anna Ma^1^, Qiufeng Wang^1^

1. Key Laboratory of Ecosystem Network Observation and Modeling, Institute of Geographic Sciences and Natural Resources Research, CAS, Beijing 100101, PR China
2. Key laboratory of Ecological Restoration in the Hilly Area, Pingdingshan University, Pingdingshan, He’nan Province, 467000, PR China

**Short title:** Carbon storage in China’s forest ecosystems

* Corresponding author. N.P. He and G.R. Yu

Institute of Geographic Sciences and Natural Resources Research, CAS

Beijing 100101

PR China

Tel. 010-64889263

E-mail: [henp@igsnrr.ac.cn](mailto:henp@igsnrr.ac.cn), yugr@igsnrr.ac.cn

**Supplementary Material**

**Appendix S1: China’s forest classification at the different scales was based on the Vegetation Map of China at 1:1000000 scales^†^**

| **6 forest type groups** | **16 forest types** | **38 forest subtypes**^‡^ |
| --- | --- | --- |
| Cold and temperate coniferous forests | Coniferous forests in cold-temperate zone and on mountains in temperate zone | ***Larix*** (*Larix gmelinii*, *Larix olgensis* var. *changpaiensis*, *Larix sibirica*, *Larix principis-rupprechti*, *Larix kaempferi*),  ***Pinus sylvestris* var. *mongolica***  ***Pieca* and *Abies* forests in North China** (*Picea crassifolia*, *Picea jezoensi*, *Picea wilsonii*, *Picea meyeri*, *Picea schrenkiana*). |
|  | Coniferous forests on mountains in subtropical and tropical zones | ***Picea* and *Abies* forests in South China** (*Picea likiangensis* var. *balfouriana*, *Picea koraiensis*, *Nakai*,*Picea likiangensis* var. *linzhiensis, Picea purpurea, Picea purpurea*), *(Abies chensiensi, Abies fargesii*, *Abies georgei*, *Abies fabri*, *Abies georgei* var. *smithii*, *Pinus densata*). |
| Temperate Coniferous Forests | Coniferous forests in temperate zone | ***Pinus tabulaeformis***  ***Pinus thunbergii***  ***Platycladus orientalis***  **Other coniferous forests in temperate zone** (*Pinus densifora*, *Pinus bungeana*) |
| Warm Coniferous Forests | Coniferous forests in subtropical an tropical zones | ***Pinus massoniana***  ***Pinus armandii***  ***Pinus yunnanensis*, *Pinus kesiya***  ***Cupressus funebris***  ***Cryptomeria fortune***  ***Cunninghamia landceolata***  ***Metasequoia glyptostroboides***  **Other coniferous forests in subtropical and tropical zones** (*pinus elliottii*, *Pinus fenzeliana*, *Pinus henryi*, *Pinus taiwanensis*). |
| Coniferous and broadleaf mixed forests | Coniferous mixed deciduous broadleaf forests in temperate zone | ***Pinus koraiensis*** (*Pinus koraiensis+Betula*, *Pinus koraiensis*+*Tilia amurensis*, *Pinus koraiensis*+*Quecrus liaotungensi*). |
|  | Coniferous evergreen and deciduous broadleaf mixed forests on mountains in subtropical zone | **Coniferous evergreen and deciduous broadleaf mixed forests on mountains in subtropical zone** (*Tsuga chinensis*+*Acer*+*Betula*, *Tsuga dumosa*+*Schima mornhae*, *Tsuga dumosa*+*Tetracenton sinense*, *Tsuga dumosa*+*Quercus semicarpifolia*). |
| Deciduous broadleaf forest | Deciduous broadleaf forests in temperate zone | ***Populus*** (*Populus davidiana, Populus nigra, Populus simonii, Populus nigra* var. *italic*),  ***Betula*** (*Betula albo-sinensis, Betula platyphylla, Betula ermanii elfin* )*,*  ***Quercus*** (*Quercus mongolica, Quecrus liaotu, Quercus aliena, Quercus aliena* var. *acuteserrataMaxim, Quercus variabilis*),  ***Tilia tuan***  ***Fraxinus mandschurica*, *Juglans mandshurica*, and *Phellodendron amurense* forests**  ***Robinia pseudoacacia***. |
|  | Deciduous microphyllous woodland in temperate zone | **Deciduous microphyllous woodland in temperate zone** (*Ulmus, Populus pruinosa*) |
|  | Deciduous broadleaf forests in subtropical zone | **Deciduous broadleaf forests in subtropical zone（***Liquidambar formosana*, *Sassafras tzumu*, *Quercus variabilis, Quercus glandulifera var.brevipetiolata, Quercus acutissima,* *Populus davidiana, Populus Adenopoda, Betula utilis Betula luminifera, Alnus cremastogyne*, *Paulownia fortune*). |
| Evergreen broadleaf forests | Evergreen broadleaf and deciduous mixed forests in subtropical zone | **Evergreen broadleaf and deciduous mixed forests in subtropical zone** (*Quercus* and evergreen broadleaf mixed forests, *Quercus acutissimaCyclobalanopsis*, *Cyclobalanopsis glauca*+*Platycarya strobilacea*, *Cyclobalanopsis glauca*+*Celtis sinensis*) |
|  | Evergreen broadleaf forests in subtropical zone | ***Schima superb*, *Castanopsis*, and *Cyclobalanopsis glauca* forests**  ***Phoebe*, *Machilus***  ***Cinnamomum longepaniculatum*** |
|  | Sclerophyllus broadleaf evergreen forests in subtropical zone | **Sclerophyllus broadleaf evergreen forests in subtropical zone** (*Quercus aquifolioides*, *Quercus rehderiana*, *Quercus senescens*, *Quercus cocciferoides*, etc) |
|  | Other evergreen broadleaf forests | **Other evergreen broadleaf forests** (*Lithocarpus glaber*, *Rhodoleia championii*, *Manglietia fordiana*) |
|  | Fast grown evergreen broadleaf plantation forest in subtropical zones | ***Eucalyptus***  ***Acacia confuse***  ***Casuarina equisetifolia*** |
|  | Bamboo forests and scrubs in subtropical and tropical zones | **Bamboo forests and scrubs in subtropical and tropical zones** (*Phyllostachys pubescens*, *Phyllostachys bambusoides*, *Phyllostachys congesta* scrub, *Pleioblastus amarus*, *Sinocalamus affinis*, *Bambusa textilis*, *Dendrocalamus strictus*, etc) |
|  | Tropical monsoon rain forests | **Tropical monsoon rain forests** (*Schima Reinw*, *Ficus altissima*, *Chukrasia tabularis*,etc) |
|  | Tropical rain forests | **Tropical rain forests** (*Terminalia myriocarpa*, *Pometia pinnata*, *Parashorea chinensi*, *Excentrodendron hsienmu*, *Madhuca pasquieri*, etc) |

†Forest classifications were based on the principles of Chinese vegetation regionalization (Chinese Academy of Sciences 2001; Hou et al.1982; Li et al. 2011). China’s forests are classified into 6 forest type groups, 16 forest types, and 38 forest subtypes.

‡ Forest subtypes marked with the bold letters are on behalf of 38 kinds of forest subtypes.

**Appendix S2:** Data used in this study collected from the published literature during the period from 2004 to 2014.

1. Ai, Z.M., Chen, Y.M., Cao, Y., 2014. Storage and allocation of carbon and nitrogen in *Robinia pseudoacacia* plantation at different ages in the loess hilly region, China. *Chinese Journal of Applied Ecology* 25, 333-341. (in Chinese)
2. Aminem, E.L.I., Chang, S.L., Zhang, Y.T. *et al.* 2014. Altitudinal distribution rule of *Picea schrenkiana* forest's soil organic carbon and its influencing factors. *Acta Ecologica Sinica* 34, 1626-1634. (in Chinese)
3. Bai, F., 2007. Forest structure, vascular plant species biodiversity and carbon storage change for 43 years on the north slope of Changbai Mountains Natural Reserve, China. *Master Dissertation of the Chinese Academy of Sciences*. (in Chinese)
4. Bao, C.S., 2010. Studies on productivity and carbon flux of *Larix Gmelinii* forest ecosystem. *Master Dissertation of the Chinese Academy of Forestry*. (in Chinese)
5. Bao, Y.L., Niu, S.K., Zhang, G.L., 2009. Study on carbon storage in the forest of *Picea schrenkiana*. *Journal of Arid Land Resources and Environment* 23, 113-117.
6. Bi, J., Wang, C., Fixation, C.B., 2011. Ability of forest ecosystems in Mulanweichang and its Characteristics. *Journal of Northeast Forestry University* 39, 45-48. (in Chinese)
7. Bu, W.S., 2013. The relationships between biodiversity and ecosystem function in natural tropical forests of Hainan Island, China. *Doctoral Dissertation of the Chinese Academy of Forestry*. (in Chinese)
8. Bu, X.L., Ruan, H.H., Wang, L.M., *et al.* 2012. Soil organic matter in density fractions as related to vegetation changes along an altitude gradient in the Wuyi Mountains, southeastern China. *Applied Soil Science* 52: 42-47.
9. Cai, S., 2012. Study on biomass of three forest types in Jingouling forest region. *Doctoral Dissertation of Beijing Forestry University.* (in Chinese)
10. Cai, Z.K., 2010. Impacts of nitrogen deposition on soil carbon content and activity of soil *Cellulose Enzymes* in the Chinese fir plantation. *Master Dissertation of Fujian Agriculture and Forestry University*. (in Chinese)
11. Cao, J., 2012. Study on biomass and carbon storage of vegetation of under forest in Guiyang in Krast area. *Master Dissertation of Central South University of Forestry and Technology*. (in Chinese)
12. Cao, J.X., 2011. Above-and belowground carbon pools in different ages of Chinese pine and oriental arborvitae plantation forests in Northern Mountain areas of Beijing. *Master Dissertation of Beijing Forestry University*. (in Chinese)
13. Cao, X.Y., Li, J.P., 2014. Storage and distribution of soil organic carbon in different age-group Chinese fir plantations. *Journal of Central South University of Forestry & Technology* 34, 104-107. (in Chinese)
14. Cao, X.Y., Wang, B.T., Chi, L., *et al.* 2013. The relationship between soil nutrient and carbon density of three forest stands on Lüliang Mountains, Shanxi Province. *Journal of Arid Land Resources and Environment* 27, 86-90. (in Chinese)
15. Cao, Y.S., Li, F.S., Lu, S.W., *et al.* 2012. Studies on biomass and productivity of main forest in the eastern Inner Mongolia Mountains. *Journal of Inner Mongolia Agricultural University* (*Natural Science Edition*) 33, 52-57. (in Chinese)
16. Chan, O.C., Casper, P., Sha, L.Q., *et al.* 2008. Vegetation cover of forest, shrub and pasture strongly influences soil bacterial community structure as revealed by 16S rRNA gene T-RFLP analysis. *FEMS Microbiology Ecology* 64, 449-458.
17. Chang, R.Y., Fu, B.J., Liu, G.H., *et al.* 2012. Effects of afforestation on soil organic and inorganic carbon: A case study of the Loess Plateau of China*. Catena* 95, 145-152.
18. Chang, R.Y., Jin, T.T., Lu, Y.H., *et al.* 2014. Soil carbon and nitrogen changes following afforestation of marginal cropland across a precipitation gradient in Loess Plateau of China. *PLoS ONE* 9, e0085426.
19. Chen, L.Z., 2007. Studies on soil organic carbon of main forest vegetation types in the Three-Gorges Reservior area. *Doctoral Dissertation of Beijing Forestry University*. (in Chinese)
20. Chen, D., Zhang, Y., Lin, Y.B., *et al.* 2010. Changes in belowground carbon in *Acacia crassicarpa* and *Eucalyptus urophylla* plantations after tree girdling. *Plant and Soil* 326, 123-135.
21. Chen, F.S., Zhang, Y.M., Hu, X.F., *et al.* 2012. The pattern of ecosystem carbon stock in steep slope wild shrubs and neighboring forest plantations in hilly red soil area. *Journal of Soil and Water Conservation,* 26, 151-155. (in Chinese)
22. Chen, G.S., 2009. Soil respiration and total belowground carbon allocation along an age chronosequence of Chinese fir plantation. *Doctoral Dissertation of Fujian normal university*. (in Chinese)
23. Chen, G.S., Yang, Y.S., Xie, J.S., *et al.* 2004. Soil biological changes for a natural forest and two plantations in subtropical China, *Pedosphere* 14, 297-304.
24. Chen, G.S., Yang, Z.J., Gao, R., *et al.* 2013. Carbon storage in a chronosequence of Chinese fir plantations in southern China. *Forest Ecology and Management* 300,68-76.
25. Chen, J.Y., 2008. Soil organic carbon dynamics and general model in several different forest ecosystems in temperate zones. *Master Dissertation of Nanjing agricultural university*. (in Chinese)
26. Chen, L., 2013. Carbon stocks and soil carbon cycle in Ginkgo agro-forestry systems. *Doctoral Dissertation of Nanjing Forestry University.* (in Chinese)
27. Chen, L.Z., Zeng, X.Q., Nora, F.Y., *et al.* 2012. Comparing carbon sequestration and stand structure of monoculture and mixed mangrove plantations of *Sonneratia caseolaris* and *S. apetalain* in Southern China. *Forest Ecology and Management* 284, 222-229.
28. Chen, Q., Zheng, Z., Feng, Z.L., *et al.* 2014. Biomass and carbon storage of *Pinus kesiya* var. *langbianensis* in Pu-Er district, Yunnan Province. *Journal of Yunnan University* 36, 439 -445. (in Chinese)
29. Chen, X., 2007. Analyzing features of leaf area index, net primary productivity and tree-ring for typical forest communities in warm temperate zone. *Master Dissertation of Institute of Botany, The Chinese Academy of Sciences* (in Chinese)
30. Chen, X.B., Zheng, H., Zhang, W., *et al.* 2014. Effect of land cover on soil organic carbon stock in a Karst landscape with discontinuous soil distribution. *Journal of Mountain Science* 11, 774-781.
31. Chen, X.G., Zhang, X.Q., Zhang, Y.P., *et al.* 2009. Changes of carbon stocks in bamboo stands in China during 100 years. *Forest Ecology and Management* 258, 1489-1496.
32. Chen, Y.F., 2014. Dynamic analysis of vegetation biomass and carbon storage of public welfare forests in Liandu District. *Journal of Sichuan Forestry Science and Technology* 35: 66-69. (in Chinese)
33. Chen, Z.H., Zhang, H.D., Wang, B.S., *et al.* 1993. Studies on Biomass and its allocation of the Evergreen broadleaved forest in Heishiding, Guangdong. *Journal of Plant Ecology* 17, 289-298. (in Chinese)
34. Cheng, D.X., Li, Y.D., Liu, H.P., *et al.* 2010. Biomass and carbon dynamics of a tropical mountain rain forest in China. *Science China* 53, 798-810.
35. Cheng, H., 2013. Carbon dynamic of *Betula platyphylla* natural secondary forest in north region of Yanshan Mountain. (in Chinese)
36. Cheng, J.M., Zhou, H.Y., 1990. Study on forest biomass of Liupanshan Mountain and its eco-hydrology. *Journal of Beijing Forestry University* 12, 55-63. (in Chinese)
37. Cheng, T.G., 2007. Research on the forest biomass and carbon Storage in Xiaolong Mountains, Gansu Province. *Master Dissertation of Beijing Forestry University*. (in Chinese)
38. Cheng, X.L., Yang, Y.H., Li, M., *et al.* 2013. The impact of agricultural land use changes on soil organic carbon dynamics in the Danjiangkou Reservoir area of China. *Plant and Soil* 366, 415-424.
39. Cheng, X.Q., Han, H.R., Kang, H.F., 2012. Biomass, carbon accumulation and its partitioning of a *Pinus tabulaeformis* plantation ecosystem in Shanxi Province, China. *Chinese Journal of Ecology* 31, 2455-2460. (in Chinese)
40. Cheng, X.R., Yu, M.K., Wu, T.G., *et al.* 2012. Effect of site condition on carbon storage of *Quercus acutissima* plantations. *Ecology and Environmental Sciences* 21, 1674-1677. (in Chinese)
41. Chi, L., Wang, B.T., Cao, X.Y., *et al.* 2014. Carbon storage of Chinese pine forest ecosystem in the central Shanxi province. *Journal of Arid Land Resources and Environment* 28, 81-85. (in Chinese)
42. Chi, L., Wang, B.T., Cao, X.Y., 2014. Carbon storage of Chinese pine forest ecosystem in the central Shanxi province. *Journal of Arid Land Resources and Environment* 28, 81-85. (in Chinese)
43. Chi, L., Wang, B.T., Cao, X.Y., *et al.* 2013. Carbon storage and density of *Pinus tabulaeformis* and *Quercus liaotungensis* forests in Taiyue Mountain of Shanxi Province, China. *Journal of Northeast Forestry University* 41, 32-35. (in Chinese)
44. Chi, X.W., 2013. Research of carbon storage function and nitrogen storage function of *Metasequoia glyptostroboides* and *Japanese cedar* in Huaxi rainy area. *Master Dissertation of Sichuan Agricultural University*. (in Chinese)
45. Cui, H.X., Xiao, W.F., Pan, L., *et al.* 2012. Characteristics of soil carbon storage of *Abies fargesii* forest in Shennongjia. *Scientia Silvae Sinicae* 48, 107-111. (in Chinese)
46. Cui, J., 2013. Soil Carbon sequestration characteristics of oak forests and influencing factors in Shanxi Province. *Master Dissertation of Northwest Agriculture and Forestry University*. (in Chinese)
47. De Blécourt, M., Brumme, M., Xu, J.C., *et al.* 2013. Soil carbon stocks decrease following conversion of secondary forests to rubber (*Hevea brasiliensis*) plantations. *PLoS ONE* 8, e0069357.
48. De Blécourt. M., Hänsel, V.M., Brumme, R., *et al.* 2014. Soil redistribution by terracing alleviates soil organic carbon losses caused by forest conversion to rubber plantation. *Forest Ecology and Management* 313, 26-33.
49. Deng, H., Zhang, B., Yin, R., *et al.* 2010. Long-term effect of re-vegetation on the microbial community of a severely eroded soil in sub-tropical China. *Plant and Soil* 328, 447-458.
50. Deng, J., Shangguan, Z.P., 2009. Nutrient and carbon pools in both natural and artificial Pinus tabulae form is in Ziwuling Region. *Acta Ecologica Sinica* 29, 3231-3240. (in Chinese)
51. Deng, L., Wang, K.B., Chen, M.L., *et al.* 2013. Soil organic carbon storage capacity positively related to forest succession on the Loess Plateau, China. *Catena* 110, 1-7.
52. Deng, X.W., Han, S.J., Hu, Y.L., *et al.* 2009. Carbon and nitrogen transformations in surface soils under *Ermans Birch* and dark coniferous Forests. *Pedosphere* 19, 230-237.
53. Dong, D., Lin, T.N., Tang, J.G., *et al.* 2014. Biomass allocation patterns and allometric models of *Tilia amurensis*. *Journal of Beijing Forestry University* 36, 54-63. (in Chinese)
54. Dong, D.R., 2012. Aboveground biomass estimation of *Populus Euphratica* and *Tamarix* community with Remote Sensing in the Lower Reaches of Tarim River. *Master Dissertation of Xinjiang agricultural university*. (in Chinese)
55. Dong, J.X., 2012. Study on carbon storage and influence factors of *Pinus Taiwanensis* in Daiyun Mountain. *Master Dissertation of Fujian Agriculture and Forestry University*. (in Chinese)
56. Dong, Y.Z., Wang, Y.L., Zhang, J.J., *et al.* 2014. Soil carbon and nitrogen storage of different land use types in northwestern Shanxi Loess Plateau Chinese *Journal of Applied Ecology* 25, 955-960. (in Chinese)
57. Dou, X.L., Deng, Q., Li, M., *et al.* 2013. Reforestation of *Pinus massoniana* alters soil organic carbon and nitrogen dynamics in eroded soil in south China. *Ecological Engineering* 52, 154-160.
58. Du, H., Song, T.Q., Zeng, F.P., *et al.* 2013. Biomass and its allocation in *Pinus massoniana* plantation at different stand ages in East Guangxi. *Acta Botanica Boreali-Occidentalia Sinica* 33, 0394-0400. (in Chinese)
59. Du, M.Y., 2010. The carbon pool in different types of *Phyllostachys edulis* stands. *Master Dissertation of Chinese Academy of Forestry.* (in Chinese)
60. Du, Y.X., Song, Z.X., He, C.L., *et al.* 2013. Organic carbon content and influencing factors of different forest soils in Jiujiang, Jiangxi Province. *Chinese Journal of Soil Science* 44, 576-581. (in Chinese)
61. Du, Y.X., Wu, C.J., Zhou, S.X., *et al.* 2011. Forest soil organic carbon density and its distribution characteristics along an altitudinal gradient in Lushan Mountains of China. *Chinese Journal of Applied Ecology* 22, 1675-1681. (in Chinese)
62. Fan, X., Tian, D.L., Fan, W., *et al.* 2014. Carbon content of main agro-forestry tree species in the North China Plain. *Journal of Central South University of Forestry & Technology* 34: 85-87. (in Chinese)
63. Fan, Y.X., 2011. The Soil Carbon Pool and Soil Respiration during Natural Succession of Mid-subtropical Evergreen Broadleaved Forest. *Fujian Normal University*. (in Chinese)
64. Fang, H.Y., 2013. Impacts of nitrogen deposition on carbon pool and carbon sequestration benefits in the Chinese fir plantation. *Master Dissertation of Jiangxi Agriculture University*. (in Chinese)
65. Fang, J.P., 2012. Study on biomass and productivity of *Picea likiangensis* var. linzhiensis forest in Nanyigou of Tibet. *Forest Research* 25, 582-589.(in Chinese)
66. Fang, J.P., Xiang, W.H., 2008. Biomass and its distribution of a primeval *Abies georgei* var. smithii forest in Sejila Mountain in Tibet Plateau. *Scientia Silvae Sinicae* 44, 17-23. (in Chinese)
67. Fang, J.Y., Liu, G.H., Zhu, B., *et al.* 2007. Carbon budgets of three temperate forest ecosystems in Dongling Mt. Beijing, China*. Science China Serires D-Earth* *Science* 50, 92-101.
68. Fang, J.Y., Liu, G.H., Zhu, B., *et al.* 2006. Three temperate forest ecosystem carbon cycle in Dong lingshan, Beijing. *Science China Serires D-Earth* *Science* 36, 533-543. (in Chinese)
69. Fang, S.Z., Tian, D., Yang, L.L., *et al.* 2010. Carbon density, Carbon stock and carbon sequestration in *Alnus cremastogyne* plantation. *Scientia Silvae Sinicae* 46, 15-21. (in Chinese)
70. Fang, X.M., Wang, Q.L., Zhou, W.M., *et al.* 2014. Land use effects on soil organic carbon, microbial biomass and microbial activity in Changbai Mountains of Northeast China. *Chinese Geographical Science* 24, 297-306.
71. Fu, W.J., Jiang, P.K., Zhao, K.L., *et al.* 2014. The carbon storage in moso bamboo plantation and its spatial variation in Anji County of southeastern China. *Soil Sediments* 14, 320-329.
72. Gao, H.L., 2013. Distribution of organic carbon and nitrogen in soil aggregates of woodlands in the west of the Loess Plateau. *Master Dissertation of northwest agriculture and Forestry University of science and technology*. (in Chinese)
73. Gao, X., Ding, G.J., Zhai, S.S., *et al.* 2014. Spatial distribution of root biomass of *Pinus massoniana* plantations under different planting densities. *Journal Of Central South University of Forestry & Technology* 34, 71-75 (in Chinese)
74. Gao, Y., Cheng, J.M., Ma, Z.R., *et al.* Carbon storage in biomass, litter, and soil of different plantations in a semiarid temperate region of northwest China. *Annals of Forest Science* 71, 427-435.
75. Ge, X.G., Huang, Z.L., Cheng, R.M., *et al.* 2012. Effects of litter fall and root input on soil physical and chemical properties in *Pinus massoniana* plantations in Three Gorges Reservoir Area, China. *Chinese Journal of Applied Ecology* 23, 3301-3308. (in Chinese)
76. Gong, C., Wang, S.L., Zeng, Z.Q., *et al.* 2011. Carbon storage and its distribution pattern of evergreen broad-leaved forests at different succession stages in mid-subtropical China. *Chinese Journal of Ecology* 30, 1935-1941.
77. Gong, W., Hu, T.X., Wang, J.Y., *et al.* 2008. Soil carbon pool and fertility under natural evergreen broad-leaved forest and its artificial regeneration forests in Southern Sichuan Province 28, 2537-2547. (in Chinese)
78. Gong, W., Hu, T.X., Wang, J.Y., *et al.* 2008. Soil carbon pool and fertility under natural evergreen broadleaved forest and its artificial regeneration forests in Southern Sichuan Province, China. *Acta Ecological Sinica* 28, 2536-2545. (in Chinese)
79. Gu, W., 2007. Carbon sequestration in soils of rehabilitated plantations on severely eroded lands in tropical China. *Master Dissertation of South China Botanical Garden, Chinese academy of sciences*. (in Chinese)
80. Guan, J.R., Yi, L.T., Qian, Y.F., *et al.* 2012. The Biomass and carbon storage in public welfare forest in Shengzhou City. *Hubei Agricultural Sciences* 51, 4556-4573. (in Chinese)
81. Guo, B.H., Fan, S.H., Du, M.Y., *et al.* 2014. Effect of land-use type on soil labile carbon pool and carbon management index. *Chinese Journal of Ecology* 33, 723-728.
82. Guo, J.F., Yang, Y.S., Chen, G.S., *et al.* 2005. Carbon and nitrogen pools in Chinese fir and evergreen broadleaved forests and changes associated with felling and burning in mid-subtropical China. *Forest Ecology and Management* 216, 216-226.
83. Guo, J.R., Zhuang, J.J., Zhu, X.L., *et al.* 2012. Influence of different age on soil physical properties and water conservation of *Quercus aliena* var. Acuteserrata forest in Baotianman Nature Reserve. *Journal of Henan Agricultural University* 46, 549-576. (in Chinese)
84. Guo, L.D., Zhou, Y., Zhong, X.L., *et al.* 2009. Evaluation of Carbon Sequestration Function and Its Economic Value of the Eucalyptus urophylla Plantation in Western Pearl River Basin. *Guangdong Forestry* *Science and Technology* 25, 8-13. (in Chinese)
85. Guo, Q., Wang, X.J., Yi, X.D., 2014. Correlation of understory biomass and soil under *Cunninghamia lanceolata* pure forest of different age. *Journal of Northeast Forestry University* 42, 85-88. (in Chinese)
86. Guo, X.Y., Cai, T., Duan, X.W., *et al.* 2013. Carbon storage and distribution pattern in main economic fruit forest ecosystems in Shanghai, East China. *Chinese Journal of Ecology* 32, 2881-2885. (in Chinese)
87. Guo, Y.F., Yao, Y.F., Qin, F.C., *et al.* 2013. Carbon stocks and carbon sequestration potentials in ecosystems of two afforestation species in typical watershed of Yanshan Mountains. *Ecology and Environment Sciences* 22, 1665-1670. (in Chinese)
88. Guo, Y.Q., 2010. Soil organic carbon characteristics of *Jatropha curcas* plantation ecosystem in dry-hot valley region in Yunnan Province, China. *Doctoral Dissertation of Nanjing Forestry University*. (in Chinese)
89. Hai, L., 2009. Studies on carbon sequestration of *Larix Gmelinii* virgin forest and restoration forest after cutting. *Master Dissertation of Inner Mongolia Agricultural University*. (in Chinese)
90. Han, B.B., Xiao, Z.D., Fu, S.L., *et al.* 2014. Research on carbon storage of poplar-crop agroforestry ecosystem with different planting densities. *Journal of Anhui Agricultural University* 41, 130-135. (in Chinese)
91. Han, J.J., Cheng, J.M., Wan, H.I., *et al.* 2010. Carbon density of *Quercus liaotungensis* community in Ziwuling. *Journal of Northwest Forestry University* 25, 18-23. (in Chinese)
92. Han, X.H., Tong, X.G., Yang, G.H., *et al.* 2012. Difference analysis of soil organic carbon pool in returning farmland to forest in loess hilly area. *Transactions of the Chinese Society of Agricultural Engineering* 28, 223-229. (in Chinese)
93. Hao, Y.P., Miao, T.T., Liu, S.Q., 2012. Research on the forest carbon storage in artificial poplar plantations in Yixiu district of Anhui Province. *Journal of Sichuan Forestry science and technology* 33, 65-69. (in Chinese)
94. He, B., Huang, S.X., Zhao, L.J., *et al.* 2009. Dynamic characteristics of carbon accumulation in *Taiwania flousiana* plantation ecosystem. *Scientia Silvae Sinicae* 45, 151-157. (in Chinese)
95. He, B., Liu, Y.H., Yu, H.G., *et al.* 2009. Carbon density and storage of *Acacia mangium* plantation ecosystem in Nanning, Guangxi. *Scientia Silvae Sinicae* 45, 6-11. (in Chinese)
96. He, J.N., Xie, J.T., Xiao, Y.F., *et al.* 2014. Studies of soil organic carbon and its spatial distribution in mountain Mangshan. *Journal of Central South University of Forestry & Technology* 34, 72-76. (in Chinese)
97. He, N., 2012. Karst peak cluster depression forest ecosystem carbon distribution and its formation mechanism. *Master Dissertation of institute of subtropical ecological agriculture, the Chinese Academy of Sciences.* (in Chinese)
98. He, Y.J., Qin, L., Li, Z.Y., *et al.* 2012. Carbon storage capacity of a *Betula alnoides* stand and a mixed *Betula alnoides*×*Castanopsis hystrix* stand in Southern Subtropical China: a comparison study. *Acta Ecologica Sinica* 32, 7586-7594. (in Chinese)
99. He, Y.J., Zheng, Y., Wang, W., *et al.* 2013. Response of Soil Physicochemical Properties to Different Management Modes in Natural Secondary Coniferous Forests. *Journal of Northeast Forestry University* 41, 63-68. (in Chinese)
100. Hong, T., 2009. Study on carbon storage of *Aleurites montana* plantation ecosystem. *Master Dissertation of Fujian Agriculture and Forestry University*. (in Chinese)
101. Hong, X.Q., 2012. Study on soil organic carbon density and the influenced factors in the main forest community types in Xinganling. *Master Dissertation of The northeast forestry university*. (in Chinese)
102. Hu, F.H., Liu, G.H., 2013. Dynamics of soil physical-chemical properties and organic carbon content along a restoration chronosequence in *Pinus tabulaeformis* plantations. *Acta Ecologica Sinica* 33, 1212-1218. (in Chinese)
103. Hu, H.F., 2006. Dynamics of soil properties and organic carbon along the chronosequence of vegetation rehabilitation: A case study of pine plantation in the upper Minjiang River region, China. *Master Dissertation of graduate university of Chinese academy of sciences*. (in Chinese)
104. Hu, Q., 2012. Carbon storage and carbon sequestration potential of forests in the central of three provinces. *Doctoral Dissertation of Chinese academy of sciences*. (in Chinese)
105. Hu, S.S., 2012. Research on the Biomass of typical of main forest types in Xinjiang. *Master Dissertation of Xinjiang Agricultural University*. (in Chinese)
106. Hu, Z.D., Liu, S.R., Shi, Z.M., Liu, X.L., He, F.,2012. Soil Particle Composition and Its Relationship with Nutrient Contents in a Quercus aquifolioides Forest at Different Altitudinal Gradient. S*cienta Silvae Sinicae* 48, 1-6.
107. Hua, Y.L., Zeng, D.H., Fan, Z.P., *et al.* 2008. Changes in ecosystem carbon stocks following grassland afforestation of semiarid sandy soil in the southeastern Keerqin Sandy Lands, China. *Journal of Arid Environments* 72, 2193-2200.
108. Huang, D.Q., 2011. Biomass and Carbon storage estimation of *Larix Gmelinii* plantation in different forest age. *Master Dissertation of Northeast Forestry University*. (in Chinese）
109. Huang, G.S., Ma, W., Wang, X.J., *et al.* 2014. Carbon storage measurement of larch forest in Northeastern China. *Scientia Silvae Sinicae* 50, 167-174. (in Chinese)
110. Huang, L., 2007. Preliminary Study on the Carbon Storage under the Different Kinds of Plantations and the Influence of Environment Monitoring of Different Vegetation in the Loess Plateau and Qin Ling Area. *Northwest agriculture and Forestry University*. (in Chinese)
111. Huang, Q.F., 2011. Structure and biomass of dominant tree of natural secondary broad-leaved forest in the low mountain of Southern Anhui. *Master Dissertation of Nanjing Forestry University*. (in Chinese)
112. Huang, R., Wang, C., Yang, Z.J., *et al.* 2011. Allocation of carbon storage in the arbor layer of young and old-growth evergreen broad-leaved forests in Wanmulin. *Journal of Subtropical Resources and Environment* 6, 29-35. (in Chinese)
113. Huang, S.D., Wu, Q.B., Liao, K.B., *et al.* 2011. Carbon storage and its allocation in an artificial *Tsoongiodendron odorum* ecosystem in southern subtropical region of China. *Chinese Journal of Ecology* 30, 2400-2404. (in Chinese)
114. Huang, X.H., Feng, D.L., Zhu, H.X., *et al.* 2014. Composition and content of soil organic carbon in *Pinus massoniana* pure forest in the Three Gorges Reservoir area of central China. *Journal of Beijing Forestry University* 36, 38-43. (in Chinese)
115. Huang, X.L., Ye, L.H., Lu, G.C., *et al.* 2013. Dynamics of soil carbon density in three plantation types. *Hunan Forestry Science & Technology* 40, 18-21. (in Chinese)
116. Huang, X.S., 2011. Study on the carbon gain model in fir and pine. *Master Dissertation of Fujian Agriculture and Forestry University*. (in Chinese)
117. Huang, Y., Wang, S.L., Feng, Z.W., *et al.* 2004. Changes in soil quality due to introduction of broad-leaf trees into clear-felled Chinese fir forest in the mid-subtropics of China. *Soil Use and Management* 20, 418-425.
118. Huang, Y.Z., 2009. Impacts of simulated nitrogen deposition on carbon pool and its chemical mechanism in the Chinese fir plantation. *Doctoral Dissertation of Fujian Agricultural University*. (in Chinese)
119. Huo, C.G., You, W.Z., Zhang, H.D., *et al.* 2011. Biomass and net primary productivity of *Quecus mongolica* plantation in Binglashan Mountains in Liaoning Province. *Liaoning Forestry Science and Technology* 241, 4-11. (in Chinese)
120. Ji, H.B., Zhuang, S.X., Zhang, H.X., *et al.* 2013. Zonality variation of carbon storage in *Phyllostachy edulis* plantation ecosystems in China. *Ecology and Environment Sciences* 22, 1-5. (in Chinese)
121. Jiang, L., Lin, N., Mo, D.X., *et al.* 2012. Study on Carbon Storage and Allocation of *Cryptomeria fortunei* Plantation in the Low Mountain of Southeast Guangxi.*Journal of Anhui Agricultural Sciences* 40, 9728-9730. (in Chinese)
122. Jiang, P., Ren, J.J., Ren, X., 2014. Biomass and carbon storage of *Populus davidiana* in typical Theropencedrymion. *Journal of Northwest Forestry University* 29, 15-20. (in Chinese)
123. Cheng, J.M., Cheng, J., Gao, Y., *et al.* 2014. Structural characteristics of community biomass in *Robinia pseudoacacia* plantations under different site conditions at Weibei loess region, northwestern China. *Journal of Beijing Forestry University* 36, 15-21. (in Chinese)
124. Jin, A.L., 2012. Study on forest biomass in BayingZhuang forest farm, Hebei Province. *Master Dissertation of Beijing Forestry University*. (in Chinese)
125. Jin, L., Lu, C.Y., Ye, Y., *et al.* 2013. Carbon Storage and Fixation by *Kandelia candel* Mangrove in Jiulongjiang Estuary. *Journal of Fujian Forestry science and technology* 40, 7-11. (in Chinese)
126. Ju, W.Z., 2010. Effects of forest ages on stand biomass and carbon storage of *Larix olgensis* plantation. *Master Dissertation of Beijing Forestry University*. (in Chinese)
127. Kang, L., 2012. Research on arborous layer aboveground biomass and gross productivity of the typical forest types on the southern slope of Qinling Mountains. *Master Dissertation of Northwest Agriculture and Forestry University*. (in Chinese)
128. Lang, F., Ye, G.F., Huang, Y.X., *et al.* 2012. Carbon storage and fraction of natural *Castanopsis eyrei* forest in the Wuyi Mountains. *Journal of Subtropical Resources and Environment* 7, 71-77. (in Chinese)
129. Li, H.L., Chen, L.B., Fang, S.Z., *et al.* 2009. Comparison of Carbon Storage and Distribution in Different Poplar-Crop Intercropping Patterns. *Scientia Silvae Sinicae* 45, 9-14. (in Chinese)
130. Li, C.H., Li, Y., Tang, L.S., 2010. Soil organic carbon stock and carbon efflux in deep soils of desert and oasis. *Environment Earth Science* 60, 549-557.
131. Li, C.Y., 2013. Biomass partitioning of laccbark pine and its tree-ring growth in Relation to Climatic factors. *Master Dissertation of Beijing Forestry University*. (in Chinese)
132. Li, D., 2006. Study on carbon storage and allocation of the monsoonal evergreen broad-leaved forests in Xishuangbanna. *Master Dissertation of Xishuangbanna tropical botanical garden, Chinese academy of sciences*. (in Chinese)
133. Li, G., Zhou, G.Y., Wu, Z.M., 2012. Aboveground biomass of a naturaly-regenerated *Schima superba* community at Xiaokeng of the Nanling Mountain. *Scientia Silvae Sinicae* 48, 143-147. (in Chinese)
134. Li, H.L., 2010. Carbon storage and carbon budget of Poplar-Crop intercropping ecosystem in the northern Jiangsu plain agricultural areas. *Doctoral Dissertation of Nanjing Forestry University*. (in Chinese)
135. Li, J., Wei, X.H., Chai, H., *et al.* 2014. Impacts of land-use types on soil C mineralization and temperature sensitivity of forests in Qianyanzhou, Jiangxi Province, China. *Chinese Journal of Applied Ecology* 25, 1919-1926. (in Chinese)
136. Li, J.Q., 2010. Study on single tree Biomass model of *Betula platypkylla* and its carbon storage in Daqing Mountain, Inner Mongolia. *Master Dissertation of Inner Mongolia Agricultural University*. (in Chinese)
137. Li, K., 2013. The distribution pattern of carbon and nitrogen in Eucalyptus plantations under different ages. *Master Dissertation of Guangxi University Agricultural Extension*. (in Chinese)
138. Li, K.Z., 2012. Effects of different improvement treatments on carbon storage of *Pinus massoniana* low-benefit forest. *Master Dissertation of Sichuan agricultural university*. (in Chinese)
139. Li, L.L., 2014. Above-ground Carbon Storage and Distributions of Main Tree Species in Natural Secondary Forest of Beijing Songshan Nature Reserve. *Journal of Anhui Agricultural Sciences* 42, 1417-1420. (in Chinese)
140. Li, L.Q., Wang, D., Liu, X.Y., *et al.* 2014. Soil organic carbon fractions and microbial community and functions under changes in vegetation: a case of vegetation succession in Karst forest. *Environmental Earth Sciences* 71, 3727-3735.
141. Li, M., Yang, G.B., Mou, Z.H., *et al.* 2013. Research on carbon storage estimate and dynamic change of Dashahe Nature Reserve Ecosystem. *Forest Resources Management* 4, 65-71. (in Chinese)
142. Li, M.R., Ding, G.J., 2013. Study on carbon storage of main forest types in southeast part of Guizhou province. *Journal of Central South University of Forestry & Technology* 33, 119-124. (in Chinese)
143. Li, N., 2008. Application of remote sensing model in biomass estimation and carbon storage of the subalpine coniferous forest in Western Sichuan Province: A case study in Daofu. *Master Dissertation of Sichuan Agricultural University*. (in Chinese)
144. Li, P., Xiao, Y., Yang, Y., *et al.* 2014. Ecosystem carbon storage in poplar plantations of different stand ages in Tianjin plain. *Chinese Journal of Ecology* 33, 567-575. (in Chinese)
145. Li, Q., Ma, M.D., Liu, Y.G., *et al.* 2007. Study on soil carbon and nutrients pools of several evergreen broad-leaved forest types in Northwest Sichuan. *Journal of Soil and Water Conservation* 21, 115-120. (in Chinese)
146. Li, Q.Y., 2008. The Research on carbon storage of *Populus*-crop intercropping system in the Huanghuaihai plain. *Master Dissertation of Henan Agricultural University*. (in Chinese)
147. Li, Q.Y., Fan, W., Yu, X.X., *et al.* 2010. Carbon storage of poplar-crop ecosystem in Eastern Henan Plain. *Chinese Journal of Applied Ecology* 21, 613- 618. (in Chinese)
148. Li, R.X., Hao， J.P., Min, J.G., *et al.* 2012. Carbon storage changes and its mechanism of different densities in *Platycladus orientalis* plantation. *Ecology and Environmental Sciences* 21, 1392-1397. (in Chinese)
149. Tu L.H., Hu, H.L., Hu, T.X., *et al.* 2011. Decomposition of different litter fractions in a subtropical bamboo ecosystem as affected by experimental nitrogen deposition. *Pedosphere* 21, 685–695.
150. Li, T.J., Liu, G.B., 2014. Age-related changes of carbon accumulation and allocation in plants and soil of black Locust forest on Loess Plateau in Ansai County, Shanxi Province of China. *Chinese Geographical Science* 24, 414-422.
151. Li, W., Zheng, Z.C., Li, Y.X., *et al.* 2014. Effects of returning farmland to tea on soil organic carbon pool in hilly region in the Western Sichuan. *Scientia Agricultura Sinica* 47, 1642-1651. (in Chinese)
152. Li, W.N., 2014. Temporal and spatial distribution patterns of organic carbon of forest soil in the northern slope of Xiaoxinganling. *Protection Forest* *science and technology* 5, 17-22. (in Chinese)
153. Li, X.F., Han, S.J., Li, Y.W., *et al.* 2005. Litter fall in main forest ecosystems of Northeast China. *Chinese Journal Of Applied Ecology* 16, 783-788. (in Chinese）
154. Li, X.F., 2006. Carbon cycle and fine root and litter production and decomposition in broad-leaved Korean pine. *Doctoral Dissertation of the Chinese Academy of Sciences.* (in Chinese)
155. Li, Y., 2010. Carbon Storage of *Cunninghamia Lanceolata* mature plantation in Shaowu, Fujian Province. *Doctoral Dissertation of Chinese Academy of Forestry*. (in Chinese)
156. Li, Y., Hou, L., Chen, J.J., *et al.* 2014. Spatial disrtribution of carbon density in pine-oak mixed forest of Qinling Mountain. *Journal of Northeast Forestry University* 42, 47-50. (in Chinese)
157. Li, Y.F., Zhang, J.J., Jiang, P.K., *et al.* 2013. Long-term intensive management effects on soil organic carbon pools and chemical composition in Moso bamboo (*Phyllostachys pubescens*) forests in subtropical China. *Forest Ecology and Management* 303, 121-130.
158. Li, Y.F., Zhou, G.M., Jiang, P.K., *et al.* 2011. Carbon accumulation and carbon forms in tissues during the growth of Young bamboo (*Phyllostachys pubescens*). *Botanical Review* 77, 278-286.
159. Li, Y.L. 2011. Effect of the undergrowth on forest carbon stock, a case study of main forest types in Huitong, Hunan Province. *Doctoral dissertation of Shenyang institute of applied ecology, Chinese academy of sciences*. (in Chinese)
160. Li, Y.Q., Wu, Q.B., Qin, D.W., 2013. The carbon storage and distribution patterns in *Castanopsis kawakamii Hayata* plantation ecosystem. *Guangdong Agricultural Sciences* 10, 181-186. (in Chinese)
161. Li, Y.Q., Brandle, J., Awada, T., *et al.* 2013. Accumulation of carbon and nitrogen in the plant-soil system after afforestation of active sand dunes in China’s Horqin sandy land. *Agriculture, Ecosystems and Environment* 177, 75-84.
162. Li, Y.W., Deng, X.B., Cao, M., *et al.* 2013. Soil restoration potential with corridor replanting engineering in the monoculture rubber plantations of Southwest China. *Ecological Engineering* 51, 169-177.
163. Li, Z.C., 2006. The Effects of land-use change on the soil organic carbon storage. *Doctoral Dissertation of the Chinese academy of forestry science*. (in Chinese)
164. Liang, F., 2013. Effects of thinning treatment on carbon dynamic of Chinese arborvitae plantation in mountain area of Beijing. *Doctoral Dissertation of Beijing Forestry University*. (in Chinese)
165. Liang, X.F., 2008. Soil physical characteristics of secondary forest responsed to the vegetation restoration on meridian hill. *Master Dissertation of Chinese Academy of Sciences*. (in Chinese)
166. Liao, X.L., 2013. Potential carbon sequestration in PR107 rubber plantation ecosystem in Qiongzhong, Hainan Province. *Master Dissertation of Hainan University.* (in Chinese)
167. Lin, B.Z., 2013. Soil organic carbon and its stability in northern main plantation forests. *Master Dissertation of Liaoning University*. (in Chinese)
168. Lin, H., Ma, X., He, Z.H., *et al.* 2014. Research on Forest Ecosystem Carbon Sinks in Jiangsu Province 1, 89-97. (in Chinese)
169. Lin, W., Li, J.Y., Zhou, P., *et al.* 2014. Spatial distribution of carbon storage of three forests ecosystem in Guangzhou,China. *Guangdong Forestry science and technology* 30, 1-7. (in Chinese)
170. Lin, X.F., 2014. Research of vertical distribution characteristics of biomass of *Castanopsis fissa* Plantations. *Jiangxi Forestry science and technology* 42, 10-17. (in Chinese)
171. Lin, W., 2010. Study on organic carbon density of forest ecosystem in Jinggangshan. *Master Dissertation of Nanchang University*. (in Chinese)
172. Liu, B.R., 2010. Changes in soil microbial biomass carbon and nitrogen under typical plant communities along an altitudinal gradient in east side of Helan Mountain. *Ecology and Environmental Sciences* 19, 883-888. (in Chinese)
173. Liu, C.F., Li, X.M., 2012. Carbon storage and sequestration by urban forests in Shenyang, China. *Urban Forestry and Urban Greening* 11, 121-128.
174. Liu, F.P., Zeng, S.G., Mo, L.J., *et al.* 2013. Effect of stand transformation on the water-holding capacities of soil and litter in *Eucalyptus urophylla* plantations. *Chinese Journal of Ecology* 32, 1111-1117. (in Chinese)
175. Liu, G., Zhu, J.Y., Ye, Y.C., *et al.* 2010. Organic carbon storage and its distribution in forest litters among forest communities in Dongguan, South China. *Journal of Mountain Science* 28, 69-75. (in Chinese)
176. Liu, G.H., 2012. *Dunn eucalyptus* plantation carbon storages and their relationships with vegetation. *Master Dissertation of Fujian Agriculture University*. (in Chinese)
177. Liu, J., 2011. Research on biological productivity and characteristics of soil nutrients of main tree species for shelterbelt in Haitan Island. *Master Dissertation of Fujian Normal University*. (in Chinese)
178. Liu, L., Wang, H.Y., Yang, X.J., *et al.* 2013. Soil organic carbon and nutrients in natural *Larix olgensis* at different stand densities. *Journal of Northeast Forestry University* 41, 51-55. (in Chinese)
179. Liu, L.Y., Ma, Y., Zhang, S.X., *et al.* 2014. Productivity and carbon fixation capacity of *Populus simonii*×*Populus nigra* under different afforestation patterns. *Journal of Northwest Forestry University* 29, 37-40. (in Chinese)
180. Liu, M.Y., Chang, Q.R., Yang, X.Y., 2010. Soil carbon fractions under different land use types in the tablelands of the Loess Plateau. *Plant Nutrition and Fertilizer Science* 16, 1418- 1425. (in Chinese)
181. Liu, Q., 2013. Effect of selective cutting on the carbon density and net primary productivity of a mixed broad leaved-Korean pine forest in Northeast China. *Chinese Journal of Applied Ecology* 24, 2709-2716. (in Chinese)
182. Liu, S.Q., 2013. Carbon Storage and Characteristics of carbon pool of plantations in Heze Shandong*. Master Dissertation of Beijing Forestry University* (in Chinese)
183. Liu, S.Q., Xin, J.H., Jia, L.M., *et al.* 2013. Carbon storage and characteristics of carbon pool of poplar plantations in Heze, Shandong Province, China. *Journal of Northeast Forestry University* 41, 1-4. (in Chinese)
184. Liu, S.S., Zhang, X.H., Gong, Y.B., *et al.* 2014. Effects of grazing disturbance on soil active organic carbon in mountain forest-arid valley ecotone in the upper reaches of Minjiang River. *Chinese Journal of Applied Ecology* 25, 359-366. (in Chinese)
185. Liu, T., 2013. Study on ecosystem carbon density and its distribution characteristics under different 'grain for green' models in hilly area of south Ningxia Province. *Master Dissertation of North West Agriculture and Forestry University*. (in Chinese)
186. Liu, T.T., 2009. Calculating biomass and carbon storage of *Poplar* plantation based on tree Structure. *Master Dissertation of Beijing Forestry University*. (in Chinese)
187. Liu, W.F., Wu, J.P., Fan, H.B., *et al.* 2013. Carbon pools in an age sequence of *Eucalyptus* plantation forests. *Ecology and Environment Sciences* 22, 12-17. (in Chinese)
188. Liu, X.M., 2012. Modeling potential distribution and spatial distribution biomass C stock of Qinghai spruce (*Picea crassifolia*) in Qilian Mountains. *Doctoral Dissertation of Gansu Agriculture University*. (in Chinese)
189. Liu, X.N., Peng, P.H., Wang, L., *et al.* 2013. Study on biomass and carbon storage of *Cryptomeria Fortune* in different stand density. *Forestry Science & Technology* 38, 31-34. (in Chinese)
190. Liu, X.P., Zhang, W.J., Cao, J.S., *et al.* 2013. Carbon storages in plantation ecosystems in sand source areas of North Beijing, China, *PLoS ONE* 8, e82208.
191. Liu, Y., Han, S.J., Lin, L., 2009. Dynamic changes in soil Nutrients of four types of forests in Changbai Mountains during Litter decomposition. *Journal of Northeast Forestry University* 37, 28-30. (in Chinese)
192. Liu, Y., Su, Y.Q., Zhang, L.G., *et al.* 2013. Study on dynamic change of organic carbon in young *Robinia pseudoacacia* plantation in Loess Plateau. *Journal of Nanjing Forestry University (Natural Science Edition)* 37, 28-32. (in Chinese)
193. Liu, Y.C., 2010. Research on the changes of aboveground biomass and NPP in recovery process for main forest types in subalpine region of Western Sichuan. *Master Dissertation of Chinese academy of forestry*. (in Chinese)
194. Liu, Y.C., Zhang, Y.D., Liu, S.R., 2012. Above-ground carbon stock evaluation with different restoration approaches using tree ring chronosequences in Southwest China. *Forest Ecology and Management* 263, 39-46.
195. Liu, Y.G., Liu, C.C., Wang, S.J., *et al.* 2013. Organic carbon storage in four ecosystem types in the Karst region of Southwestern China. *PLoS ONE* 9, e106876.
196. Liu, Y.H., 2011. The characteristics of growth and carbon sequestration and water consumption in the small watershed of Xiangshuihe, Liupan Mountains. *Doctoral Dissertation of Chinese Academy of Forestry*. (in Chinese)
197. Liu, Y.T., 2012. Carbon storage in *Manchurian Ash* and *Dahurian Larch* plantations with different stand structures. *Master Dissertation of Northeast Forestry University*. (in Chinese)
198. Liu, Z.P., 2013. Spatial distribution of soil nutrients and the impact factors across the Loess Pleteau of China. *Master Dissertation of University of Chinese Academy of Sciences*. (in Chinese)
199. Liu, Z.Q., Lang, N.J., Peng, M.J., *et al.* 2013. Hydrological effects of forest litters layer and soil layer in Jinsha river watershed of Yunnan Plateau. *Journal of Soil and Water Conservation* 27, 165-173. (in Chinese)
200. Lu, G.C., Xue, Y., Xue, L., *et al.* 2014. Distribution characteristics of soil organic carbon in cutover land of a *Cunninghamia lanceolata* stand. *Journal of Anhui Agricultural University* 41, 126-129. (in Chinese)
201. Lu, H.J., Liu, W.J., Luo, Q.P., 2011. Eco-hydrological effects of litter layer in a mountainous rubber plantation in Xishuangbanna, Southwest China. *Chinese Journal of Ecology* 30, 2129-2136. (in Chinese)
202. Lu, X., Xiang, W.H., Ren, H., *et al.* 2012. Litter biomass and its carbon and nitrogen storage in four subtropical forests in central Southern China. *Chinese Journal of Ecology* 31, 2234-2240. (in Chinese)
203. Lu, Z.L., 2010. Study on Biomass and Carbon Storage of *Jatropha Curcas* in the Lincang City of Yunnan Province. *Master Dissertation of Southwest Forestry University*. (in Chinese)
204. Luan, J.W., Liu, S.R., Zhu, X.L., *et al.* 2011. Soil carbon stocks and fluxes in a warm-temperate oak chronosequence in China, *Plant and Soil* 347, 243-253.
205. Luan, J.W., Xiang, C.H., Liu, S.R., *et al.* 2009. Assessments of the impacts of Chinese fir plantation and natural regenerated forest on soil organic matter quality at Longmen Mountain, Sichuan, China. *Geoderma* 156, 228-236.
206. Luo, J, Deng, Y.G., Tian, Y.X., et al. 2014. Effect of different forest management on the biomass and distribution pattern of undergrowth vegetation of the natural secondary forest of Pinus massoniana. Hunan Forestry Science ＆ Technology 41, 65-68. (in Chinese)
207. Luo, Y.J., Zhang, X.Q., Wang, X.K., *et al.* 2009. Biornass and its distribution patterns of *Larix principis*-*rupprechtii* plantations in northern China. *Journal of Beijing Forestry University* 31, 13-19. (in Chinese)
208. Lü, X.T., Tang, J.W., He, Y.C., *et al.* 2007. Biomass and its allocation in tropical seasonal rain forest In Xishuangbanna, Southwest China. *Journal of Plant Ecology* (Chinese Version) 31, 11-22. (in Chinese)
209. Ma, F.F., Tang, C., Zhang, C.M., *et al.* 2014. Carbon contents of *Larix kaempferi* plantation ecosystem in subtropics. *Hunan Forestry Science & Technology* 41, 23-29. (in Chinese)
210. Ma, H.L., 2013. Carbon density and its distribution in the ecosystems of the main forest types in Sanbei area. *Master Dissertation of Northwest Agriculture and Forestry University.* (in Chinese)
211. Ma, H.P., Guo, Q.Q., Liu, H.M., *et al.* 2013. Soil organic carbon pool at the western side of the sygera Mountains, Southeast Tibet, China. *Acta Ecologica Sinica* 33, 3122-3128. (in Chinese)
212. Ma, K., 2011. Influence of *Poplar* plantation on soil organic carbon in arid area. *Master Dissertation of Xinjiang University*. (in Chinese)
213. Ma, S.G., Yang, Y.S., Xie, J.S., *et al.* 2010. The Water retention characteristic of litter in six old growth natural forests compared with a *Cunninghamia lanceolata* plantation in subtropical zone. *Journal of Subtropical Resources and Environment* 5, 31-37. (in Chinese)
214. Ma, W., 2011. The Analysis of Organic Carbon Storage and Influencing Factors in Different Forest Type in Liaoheyuan. *Master Dissertation of Hebei Agricultural University*. (in Chinese)
215. Ma, W., 2013. Measurement and estimation of ecosystem carbon density for *Larix Olgensis* plantation based on FIM＆FFE-FVS. *Doctoral Dissertation of Chinese Academy of Forestry*. (in Chinese)
216. Ma, W., Sun, Y.J., Guo, X.Y., *et al.* 2010. Carbon storage of Larix olgensis plantation at different stand ages. *Acta Ecologica Sinica* 30, 4659-4667. (in Chinese)
217. Ma, Z.Q., Hartmann, H.R., Wang, H.M., *et al.* 2014. Carbon dynamics and stability between native Massson pine and exotic slash pine plantation in subtropical China, *European Journal of Forest Research* 133, 307-321.
218. Ma, Z.R., 2013. Carbon storage and carbon sequestration rate of the main vegetation types in Liupan Mountain. *Master Dissertation of* the *Chinese Academy of Sciences* *University*. (in Chinese)
219. Mei, L., 2010. Carbon Storage and Density of Artificial *Pinus Tabulaeformis* Forest in Ziwuling Area. *Master Dissertation of Northwest Agriculture and Forestry University*. (in Chinese)
220. Meng, H.J., Liu, X.D., Zhang, H.B., *et al.* 2013. Study on litters and soil moisture characteristics of different plantations in Qilian Mountains. *Journal of Central South University of Forestry & Technology* 33, 11-15. (in Chinese)
221. Meng, Y.Y., Bao, Y., Guo, Y., *et al.* Soil carbon and nitrogen content in wind throw area on Changbai Mountain after 26 years natural recovery. *Chinese Journal of Ecology* 33, 1757-1761. (in Chinese)
222. Miao, J., Zhou, C.Y., Li, S.J., *et al.* 2014. Accumulation of soil organic carbon and total nitrogen in *Pinus yunnanensis* forests at different age stages. *Chinese Journal of Applied Ecology* 25, 625 -631. (in Chinese)
223. Ming, A.G., Jia, H.Y., Tian, Z.W., *et al.* 2014. Characteristics of carbon storage and its allocation in *Erythrophleum fordii* plantations with different ages. *Chinese Journal of Applied Ecology* 25, 940-946. (in Chinese)
224. Ming, A.G., Jia, H.Y., Tao, Y., *et al.* 2012. Characteristics of carbon accumulation and allocation pattern in *Mytilaria laosensis* plantation. *Chinese Journal of Ecology* 33, 2730-2735. (in Chinese)
225. Mo, D.X., Liao, K.B., Wu, Q.B., *et al.* 2011. The carbon storage amount and spatial distribution characteristics of *Paramichelia bailonii* plantations. *Journal of Anhui Agricultural Sciences* 39, 14072-14075. (in Chinese)
226. Mo, D.X., Wu, Q.B., Ling, N., *et al.* 2012. Carbon and nitrogen storage and their allocation pattern in *Cryptomeria fortunei* plantations in southeastern Guangxi of South China*. Chinese Journal of Ecology* 31, 794-799. (in Chinese)
227. Mu, C.C., Lu, H.C., Wang, B., *et al.* 2013. Short-term effects of harvesting on carbon storage of *borealLarix gmelinii*-*Carexs chmidtiiforested* wetlands in Daxing’anling, northeast China. *Forest Ecology and Management* 293, 140-148.
228. Niu, D., Wang, S.L., Ouyang, Z.Y., 2009. Comparisons of carbon storages in *Cunninghamia lanceolata* and *Micheliam acclurei* plantations during a 22-year period in southern China. *Journal of Environmental Sciences* 21, 801-805.
229. Niu, Y., Liu, X.D., Zhao, W.J., *et al.* 2014. Characteristics and interrelation of shallow soil orange and total nitrogen of *Picea crassifolia* forest in the Qilian Mountain. *Journal of Desert Research* 34, 371-377. (in Chinese)
230. Ouyang, L.M. Zeng, D.P. Min, Q.W., *et al.* 2014. Ecological stoichiometry characteristics of soil carbon, nitrogen and phosphorus in the tea garden of drum Mountain. *Journal of Soil and Water Conservation* 28, 297-312. (in Chinese)
231. Pan, G., Ren, Y.H., Bianba, D.J., *et al.* 2008. The water holding capacity of moss and litter layers of *Abies georgei* forest of Sejila Mountain in Tibet. *Research of Soil and Water Conservation* 15, 81-83. (in Chinese)
232. Pan, P., Lü, D., OuYang, X.Z., *et al.* 2014. Study on biomass and carbon storage of natural *Pinus massoniana* forest at different stand growing stages in Central Jiangxi Province. *Journal of Jiangxi Agriculturae University* (Natural Sciences Edition) 36, 131-136. (in Chinese)
233. Pan, N., Yang, G.X., Wang, T., 2014. Carbon distribution of *Pinus armandii* in Baotianman nature reserve. *Farm staff observations of seed industry* 3, 50-51.
234. Pan, Y.J., Wang, B., Chen, B.F., *et al.* 2013. Study on carbon sink of Chinese fir plantation ecosystem in Dagangshan Mountain, Jiangxi province. *Journal of Central South Forestry University* 33, 120-125. (in Chinese）
235. Pang, J.P., 2009. Carbon storage and its allocation of rubber plantation in Xishuangbanna, Southwest China. *Master Dissertation of Chinese academy of sciences.* (in Chinese)
236. Peng, S.L., Chen, A.Q., Fang, H.D., *et al.* 2013. Effect of vegetation restoration types on soil quality in Yuanmou dry-hot valley, China. *Soil Science and Plant Nutrition* 59, 347-360.
237. Peng, X.W., 2012. Study On carbon storage of immature Grass-larch forest in Daxing’Anling District. *Forestry Science and Technology* *Information* 44, 22-23. (in Chinese)
238. Wang, Q., Wang, S., Yu, X., 2010. Decline of soil fertility during forest conversion of secondary forest to Chinese fie plantations in subtropical China. *Land degradation & development* 22, 444-452.
239. Qi, G., 2011. Northeast characteristic of *larix gmelinii* carbon library and carbon sequestration potential. *Doctoral Dissertation of Chinese academy of sciences*. (in Chinese)
240. Qi, G., Wang, Q.L., Wang, X.C., *et al.* 2013. Soil organic carbon storage in different aged *Larix gmelinii* plantations in Daxinganling Mountains of Northeast China. *Chinese Journal of Applied Ecology* 24, 10-16. (in Chinese)
241. Qi, J.F., Tang, J.W., 2008. Biomass and its allocation pattern of monsoon rain forest over limestone in Xishuangbanna of Southwest China. *Chinese Journal of Ecology* 27, 167-177. (in Chinese)
242. Qi, L., Yu, D.P., Zhou, W.M., *et al.* 2013. Impact of logging on carbon density of broadleaved-Korean pine mixed forests on Changbai Mountains. *Acta Ecologica Sinica* 33, 3065-3073. (in Chinese)
243. Qi, L.H., Du, M.Y., Fan, S.H., *et al.* 2012. Dynamics of soil organic carbon pool in *Phyllostachy edulis* forest and *P.edulis-Cunning-hamia lanceolata* mixed forest in hilly regions of central Hunan, Southern China. *Chinese Journal of Ecology* 31, 3038-3043.
244. Qin, Z.F., 2011. Impact of land use change on carbon storage in southeast of North Shaanxi Loess Plateau. *Master Dissertation of Northwest Agriculture and Forestry University*. (in Chinese)
245. Ren, J.R., 2011. Study on carbon storage and carbon density of nature *Pinus Tabuliformis* forest of Mt. Helan in Ningxia Province. *Master Dissertation of Northwest Agriculture and Forestry University*. (in Chinese)
246. Ren, Y.H., Cai, J., Yuan, J., *et al.* 2012. Carbon storage and density of tree layer of three types of forest at Huoditang forest region in the Qinling Mountains. *Journal of Henan Agricultural Sciences* 41, 73-77. (in Chinese)
247. Sha, L.Q., 2008. Carbon storage and soil CO_2_ flux of tropical seasonal rain forest, rubber tree Plantation and paddy soil in Xishuangbanna, Southwest China. *Master Dissertation of Xishuangbanna Tropical Botanical Garden, Chinese academy of sciences*. (in Chinese)
248. Shang, S.Y., Jiang, P.K., Song, Z.L. *et al.* 2013. Composition and stability of organic carbon in the top soil under different forest types in subtropical China. *Acta Ecologica Sinica* 33, 0416-0424. (in Chinese)
249. Shao, M.X., Wen, S.Z., He, G.G., *et al.* 2014. The biomass structure characteristics of *P. bournei* (Hemsl.) Yang plantation in different ages. *Journal of Central South University of Forestry & Technology* 34, 44-48. (in Chinese)
250. Shao, Y.H., 2005. Soil organic carbon dynamics and validation of InTEC Model in China’s Subtropical and Temperate Zones. *Master Dissertation of Nanjing agricultural university*. (in Chinese)
251. Shao, Y.H., Pan, J.J., Xu, X.W., *et al.* 2006. Determination of forest soil organic Carbon Pool Sizes and Turnover Rates in Changbaishan. *Journal of Soil and Water Conservation* 20, 99-102. (in Chinese)
252. Shen, B., 2013. Carbon density of *Pinus tabulaeformis* and *Quercus alinea* Var. acuteserrata forest ecosystems on the Southern slope of the middle Qinling Mountains. *Master Dissertation of Northwest Agriculture and Forestry University*. (in Chinese)
253. Shen, H.T., Zhang, W.J., Yang, X., *et al.* 2014. Carbon storage capacity of different plantation types under sandstorm source control program in Hebei Province, China. *Chinese Geographical Science* 24, 454-460.
254. Shen, H.T., Zhang, W.J., Yang, X., et al. 2014. Carbon storage capacity of different plantation types under sandstorm source control program in Hebei Province, China. *Chinese Geography Science* 24, 454-460.
255. Shen, J.P., Zhang, W.H., Li, Y.H., *et al.* 2013. Characteristics of carbon storage and sequestration of *Pinus tabulaeformis* forest Land Converted by Farmland in Loess Hilly Area. *Acta Botanica Boreali-Occidentalia Sinica* 33, 2309-2316. (in Chinese)
256. Shen, J.P., Zhang, W.H., 2014. Characteristics of carbon storage and sequestration of *Robinia pseudoacacia* forest land converted by farmland in the Hilly Loess Plateau region. *Acta Ecologica Sinica* 34, 2746-2754. (in Chinese)
257. Sheng, H., 2007. Carbon pools and belowground carbon balance of mid-subtropical evergreen broad-leaved forest. *Master Dissertation of Fujian Normal University*. (in Chinese)
258. Shi, F.C., Li, J.J., Wang, S.Q., 2008. Soil organic carbon, nitrogen and microbial properties in contrasting forest ecosystems of north-east China under different regeneration scenarios. *Acta Agriculturae Scandinavica, Section B - Soil & Plant Science* 58, 1-10.
259. Shi, Z.J., Xu, D.P., Gao, G.X., *et al.* 2011. Carbon storage and its distribution of *Eucalyptus urophylla* × *E.tereticornis* plantations in Hainan Island, Southern China. *Scientia Silvae Sinicae* 47, 21-28. (in Chinese)
260. Shirato Y., Taniyama I., Zhang, T.H., 2004. Changes in soil properties after afforestation in Horqin Sandy Land, North China. *Soil Science and Plant Nutrition* 50, 537-543.
261. Si, J., 2012. Technology of improving carbon sinks of *Populous* plantation in North China. *Master Dissertation of Beijing Forestry University*. (in Chinese)
262. Song, Q.H., Zhang, Y.P., 2010. Biomass, carbon sequestration and its potential of rubber plantations in Xishuangbanna, Southwest China. *Chinese Journal of Ecology* 29, 1887-1891. (in Chinese)
263. Song, X.C., Tang, J., Qin, Q.Y. *et al.* 2014. Mechanism of biomass accumulation and nutrient distribution in *Camellia oleifera* mature forest. *Journal of Southern Agriculture* 45, 255-258. (in Chinese)
264. Song, Y., Zhao, X.Z., Mao, Z.J., *et al.* 2013. SOC decomposition of four typical broad-leaved korean pine communities in Xiaoxinganling Mountain*.* *Acta Ecologica Sinica* 33, 443-453. (in Chinese)
265. Su, A., 2011. Analysis on the spatial and temporal variation characteristics of carbon sequestration in *Phyllostachys Edulis* plantation ecosystem of Fujian. *Master* *Dissertation of Fujian Agriculture and Forestry University*. (in Chinese)
266. Su, S.C., He, D.J., Xie, Y.L., *et al.* 2012. Comparative study on the carbon storage of different forest management models in Northern Fujian. *Chinese Agricultural Science Bulletin* 28, 45-52. (in Chinese)
267. Su, Z.Y., Ke, X.D., Zhang, S.J., 2012. Vascular plants as indicators of organic carbon gradient in subtropical forested soils*.* *Polish Journal of Environmental Studies* 21, 1393-1398.
268. Sun, H., Li, F.R., Jia, W.W., *et al.* 2013. The study on carbon content of planted *Pinus koraiensis* middle-aged forest. *Forest Engineering* 29, 19-24. (in Chinese)
269. Sun, J.C., 2011. Study on biomass and carbon stock in *Pinus tabulaeformis* plantation of Taiyue Mountain. *Master* *Dissertation of Beijing Forestry University.* (in Chinese)
270. Sun, J.L., 2009. Study on *Larix* biomass model and biomass estimation in Dongzhelenghe. *Master Dissertation of Beijing Forestry University*. (in Chinese)
271. Sun, M.O., Jia, W.W., Li, F.J., *et al.* 2014. Study on biomass of *Pinus koraiensis* plantations in Eastern Heilongjiang Province. *Bulletin of Botanical Research* 34, 232-237. (in Chinese)
272. Sun, T., 2011. Soil carbon flux and carbon storage of *Betula Platyphylla* along an age series in Xiaoxinganling Mountain, China. *Master Dissertation of Northeast Forestry University*. (in Chinese)
273. Sun, Y.J., Zhang, J., Han, A.H., *et al.* 2007. Biomass and carbon pool of *Larix gmelini* young and middle age forest in Xingan ling Mountains Inner Mongolia. *Acta Ecologica Sinica* 27, 1756-1762. (in Chinese)
274. Tao, Y.H., Long, W.G., Ma, L.Y., *et al*. 2011. Carbon storage and distribution in *Pinus masson*, fir, *eucalyptus* plantation in Liuzhou. *Guangdong agricultural science* 22, 42-45.
275. Tan, B., Wu, F.Z., Yang, W.Q., *et al.* 2011. The dynamics pattern of soil carbon and nutrients as soil thawing proceeded in the alpine/subalpine forest. *Acta Agriculturae Scandinavica*, *Section B-Soil&Plant Science* 61, 670-679.
276. Tan, G.X., Liu, Y.Q., Li, L.L., *et al.* 2014. Effects of stand structure regulation on soil labile organic carbon in *Pinus elliottii* plantation. *Chinese Journal of Applied Ecology* 25, 1307-1312.
277. Tan, Q.J., Song, T.Q., Peng, W.X., *et al.* 2014. Stability and organic carbon characteristics of soil aggregates under different ecosystems in karst canyon region. *Chinese Journal of Applied Ecology* 25, 671-678.
278. Tang, F.K., Zhou, J.X., Cui, M., *et al.* 2014. Effects of different returning farmland to forestlands on accumulation of soil organic carbon and nitrogen in typical karst area of southwestern China. *Journal of Beijing Forestry University* 36, 44-50. (in Chinese)
279. Tang, G.Y., Li, K., 2013. Tree species controls on soil carbon sequestration and carbon stability following 20 years of afforestation in a valley-type savanna. *Forest Ecology and Management* 291, 13-19.
280. Tang, J.W., Yin, J.X., Qi, J.F., *et al.* 2012. Ecosystem carbon storage of tropical forests over limestone in Xishuangbanna, Southern-West China. *Journal of Tropical Forest Science* 24, 399-407.
281. Tan, W.N., 2009. Carbon sequestration ability and its mechanism in soils of rehabilitated plantations in South China. *Doctoral Dissertation of the Chinese Academy of Sciences*. (in Chinese)
282. Tang, X.L., Fan, S.H., Qi, L.H., *et al.* 2012. Effect of different managements on carbon storage and carbon allocation in moso bamboo forest (*Phyllostachys pubescen*). *Acta Agriculturae Universitatis Jiangxiensis (Natural Sciences Edition)* 34, 736-742. (in Chinese)
283. Tao, Y.H., 2012. Forest carbon storage changes with different land use patterns in Luocheng, Guangxi Province. *Doctoral Dissertation of the Central University for Nationalities*. (in Chinese)
284. Tao, Y.H., Feng, J.C., Ma, L.Y., *et al.* 2012. Study on carbon storage of *Eucalyptus* plantations in short period in Liuzhou, Guangxi Province. *Chinese Agricultural Science Bulletin* 28, 80-84. (in Chinese)
285. Tao, Y.H., Feng, J.C., Cao, S.G., *et al.* (2012.）Study on carbon storage of *Pinus massoniana* and *Cunninghamia lanceolata* plantations at Shatang, Guangxi Province. *Journal of northwest A&F University* (Natural Science Edition) 40, 38-44. (in Chinese)
286. Tian, D.L., Wang, X.K., Fang, X., *et al.* 2011. Carbon storage and spatial distribution in different vegetation restoration patterns in Karsts area, Guizhou Province. *Scientia Silvae Sinicae* 47, 7-14. (in Chinese)
287. Tian, J., Yu, D.P., Zhou, L., *et al.* 2012. Carbon density of forest ecosystems in mountainous region of east Liaoning Province, Northeast China. *Chinese Journal of Ecology* 31, 2723-2729. (in Chinese)
288. Tian, X., 2010. Carbon storage and NPP in *Phoebe bournei* plantations based on FORECAST Model. *Master Dissertation of Jiangxi Agricultural University.* (in Chinese)
289. Tian, X., Liu, Y.Q., Wei, X.H., *et al.* 2014. Simulation of the effects of various mixing proportions on NPP and carbon storage in the mixedwood plantations of *Phoebe bournei* with Chinese fir. *Acta Agriculturae Universitatis Jiangxiensis* (Natural Sciences Edition) 36, 122-130. (in Chinese)
290. Tian, Y.W., Huang, Z.L., Xiao, W.F., 2012. Effects of plant species and litter on soil organic carbon sequestration in converted croplands in a typical watershed in Three Gorges Reservoir area of China. *Chinese Journal of Ecology,* 31, 2742-2747. (in Chinese)
291. Tong, X.G., Han, X.H., Wu, F.Q., *et al.* 2012. Variance analysis of soil carbon sequestration under three typical forest lands converted from farmland in a Loess Hilly Area. *Acta Ecologica Sinica* 32, 6396-6403.
292. Wan, X.H., Huang, Z.Q., He, Z.M., *et al.* 2013. Effects of broadleaf plantation and Chinese fir (*Cunninghamia lanceolata*) plantation on soil carbon and nitrogen pools. *Chinese Journal of Applied Ecology* 24, 345-350. (in Chinese)
293. Wang, B., Wei, W.J., Xing, Z.K., *et al.* 2012. Biomass carbon pools of *Cunninghamia lanceolata* (Lamb.) Hook forest in subtropical China: characteristics and potential. *Scandinavian* *Journal of Forest Research,* 27, 545-560.
294. Wang, C., 2011. Carbon density of *Populus tomentosa* plantations at different ages on Loess Plateau. *Master Dissertation of Northwest agriculture and Forestry University*. (in Chinese)
295. Wang, C.B., Liu, L.T., Mo, X.Y., *et al.* 2013. Analysis of carbon storage of 30 clones of *Eucalyptus* plantations. *Forest Research* 6, 661-667. (in Chinese)
296. Wang, C.M., Shao, B., Wang, R.N., 2010. Carbon sequestration potential of ecosystem of two main tree species in Northeast China. *Acta Ecologica Sinica* 30, 1764-1772. (in Chinese)
297. Wang, C.M., Ouyang, H., Shao, B., *et al.* 2006. Soil carbon changes following afforestation with *Larix olgensis* in Northeastern China. *Journal of Integrative Plant Biology* 48, 503−512.
298. Wang, D., Lü, Y.L., Xu, L., *et al.* 2013. Impact of changes in vegetation types on soil C mineralization and associated temperature sensitivity in the Changbai Mountain forests of China. *Acta Ecologica Sinica* 33, 6373-6381. (in Chinese)
299. Wang, F., 2013. Research on the carbon density and carbon balance of *Larix gmelinii* forest. *Doctoral Dissertation of Inner Mongolia Agriculture University*. (in Chinese)
300. Wang, F., Zhang, Q.L., Wang, B., *et al.* 2012. Storage and characteristics of coarse woody debris in different aged natural *Ledum palustre-Larix gmelinii* forests in Daxinganling Mountains of Northeast China. *Chinese Journal of Ecology* 31, 2981-2989. (in Chinese)
301. Wang, G., Guan, D.S., Peart, M.R., *et al.* 2013. Ecosystem carbon stocks of mangrove forest in Yingluo Bay, Guangdong Province of South China. *Forest Ecology and Management* 310, 539-546.
302. Wang, G.L., Wang, X.H., Yue, C.R., *et al.* 2012. Carbon content rate in dominant species of four forest types in Shangrila, northwest Yunnan province. *Ecology and Environment Sciences* 21, 613-619. (in Chinese)
303. Wang, G.X., Ran, F., Chang, R.Y., *et al.* 2014. Variations in the live biomass and carbon pools of *Abies georgei* along an elevation gradient on the Tibetan Plateau, China. *Forest Ecology and Management* 329, 255-263.
304. Wang, G.Z., 2013. Aboveground biomass and carbon storage of *Populus* × *Xiaohei* of afforestation of Different asexual reproduction. *Master Dissertation of Hebei agricultural university*. (in Chinese)
305. Wang, H., Liu, S.R., Wang, J.X., *et al.* 2013. Effects of tree species mixture on soil organic carbon stocks and greenhouse gas fluxes in subtropical plantations in China. *Forest Ecology and Management* 300, 4-13.
306. Wang, H., 2010. Soil Carbon Sequestration and the related processes in four subtropical plantations in Southern China. *Doctoral Dissertation of the Chinese academy of forestry science*. (in Chinese)
307. Wang, J., Chen, Y.M., Cao, Y., *et al.* 2012. Carbon concentration and carbon storage in different components of natural *Quercus wutaishanica* forest in Ziwuling of Loess Plateau, Northwest China. *Chinese Journal of Ecology* 31, 3058-3063. (in Chinese)
308. Wang, L., 2010. Environmental monitoring in the different artificial ecological Forests of Miaoling River watershed in Loess Plateau. *Master Dissertation of Northwest Agriculture and Forestry University*. (in Chinese)
309. Wang, N., Wang, B.T., Wang, R.J., *et al.* 2014. Density and distribution patterns of carbon of *Pinus tabulaeformis* forest ecosystem in Shanxi Province, China. *Journal of Basic Science and Engineering* 22, 58-68. (in Chinese)
310. Wang, N, Wang, B.T., Wang, R.J. Cao, X.Y., *et al.* 2013. Biomass allocation patterns and allometric models of *Populus Davidiana* and *Pinus Tabulaeformis* Carr in West of Shanxi Province. *Bulletin of Soil and Water Conservation* 33, 151-159. (in Chinese)
311. Wang, Q.K., Wang, S.L., Feng, Z.W., 2006. Comparison of active soil organic carbon pool between Chinese fir plantations and evergreen broadleaved forests. *Journal of Beijing Forestry University* 28, 1-6. (in Chinese)
312. Wang, Q.K., Wang, S.L., Zhang, J.W., 2009. Assessing the effects of vegetation types on carbon storage fifteen years after reforestation on a Chinese fir site. *Forest Ecology and Management* 258, 1437-1441.
313. Wang, Q.Y., Feng, L.F., Cui, D., *et al.* 2014. Carbon storage of four kinds of new varieties of poplar plantations. *Protection Forest science and technology* 3, 19-21. (in Chinese)
314. Wang, S.Q., Liu, J.Y., Yu, G.R., *et al.* 2004. Effects of land use change on the storage of soil organic carbon: a case study of the Qianyanzhou forest experimental station in China. *Climatic Change* 67, 247-255.
315. Wang, S.Z., 2013. Study on carbon storage dynamic characteristics of the second generation of Chinese fir plantation at different ages. *Master Dissertation of Central South Forestry University*. (in Chinese)
316. Wang, W.D., Zhang, Y.T., Lu, J.J., *et al.* 2012. Soil physical property and nutrient elements content in different forest types of Tianshan spruce forests. *Anhui Agriculture Science Bulletin* 18, 95-96.
317. Wang, W, Zeng, W.J., Chen, W.L., *et al.* 2013. Effects of forest age on soil autotrophic and heterotrophic respiration differ between evergreen and deciduous Forests. *PLoS ONE* 8, e80937.
318. Wang, W.J., Wang, B.T., Lü, Z., *et al.* 2013. Soil organic carbon reserve of different forests in Taiyue Mountain. *Journal of Arid Land Resources and Environment* 27, 81-86. (in Chinese)
319. Wang, W.X., Shi, Z.M., Luo, D., *et al.* 2013. Carbon and nitrogen storage under different plantations in subtropical south China. *Acta Ecologica Sinica,* 33, 925-933. (in Chinese)
320. Wang, X, Yao, Y.F., Qin, F.C., *et al.* 2014. Vertical distribution of soil organic carbon about plants in Aohan County. *The north garden* 4, 149-152. (in Chinese)
321. Wang, X.F., 2010. Study on the carbon sinks in the different kinds of plantations in Loess Plateau. *Master Dissertation of Northwest Agriculture and Forestry University*. (in Chinese)
322. Wang, X.L., Chang, Y., Chen, H.W., *et al.* 2014. Biomass allocation characteristics of the main forest ecosystems in the Daxng’anling Mountains，Heilongjiang Province. *Chinese Journal of Ecology* 33, 1437-1444.
323. Wang, X.L., Wang, Y., Shi, H.H., *et al.* 2013. Carbon storage of *Pinus thunbergii* and *Robinia pseudoacacia* plantations on Nanchangshan Island, Changdao County of Shandong Province, China. *Chinese Journal of Applied Ecology* 24, 1263-1268. (in Chinese)
324. Wang, X.L., Zhang, F.R., Zhu, T.F., *et al.* 2013. The distribution and impact factors of soil organic carbon in mountainous areas of Beijing. *Resources Science* 34, 1152-1158. (in Chinese)
325. Wang, X.Y., 2011. Distribution of carbon storage for *Larix Olgensis* plantation of different stand ages. *Doctoral Dissertation of Beijing Forestry University*. (in Chinese)
326. Wang, Y., Song, X.S., Wang, J., 2014. Effect of drying-rewetting alternation on soil carbon pool and mineralization of soil organic carbon. *Acta pedologica sinica* 51, 342-350 (in Chinese)
327. Wang, Y.F., Fu, B.J., Lü Y.H., *et al.* 2006. Effects of vegetation restoration on soil organic carbon sequestration at multiple scales in semi-arid Loess Plateau, China. *Catena* 85, 58-66.
328. Wang, Y.L., Geng, Z.C., She, Y.D., *et al.* 2014. Vertical distribution of soil active carbon and soil organic carbon storage under different forest types in the Qinling Mountains. *Chinese Journal of Applied Ecology* 25, 1569-1577.
329. Wang, Y.X., 2010. Carbon storage in main plantation ecosystems, Fujian Province. *Doctoral Dissertation of Fujian agriculture and Forestry University.* (in Chinese)
330. Wang, Z., Du, B.M., Han, Y.J., *et al.* 2014. Carbon storage of *Ligustrum lucidum* plantations in Shanghai Out-Loop Forest Belt. *Chinese Journal of Ecology* 33, 910-914.
331. Wang, Z., Han, Y.J., Kang, H.Z., *et al.* 2012. Carbon storage of main tree species plantations for water resources conservation in upper reaches of Huangpu River, Shanghai. *Chinese Journal of Ecology* 31, 1930-1935. (in Chinese)
332. Wei, H.D., Dong, B., 2013. Spatio-temporal dynamics of soil microbial biomass carbon in *Populus tremula* plantations in the southeast of Shandong Province. *Ecology and Environment Sciences* 22, 233-238. (in Chinese)
333. Wei, W., You, W.Z., Zhang, H.D., *et al.* 2014. Soil organic carbon and its impact factors in original *Pinus koraiensis* mixed forest in eastern Liaoning mountainous area. *Liaoning Forestry* *science and technology* 2, 4-8. (in Chinese)
334. Wei, W.J., 2007. Carbon density and storage of forests in Dagangshan Mountains, Jiangxi Province. *Master Dissertation of Inner Mongolia Agricultural University.* (in Chinese)
335. Wei, X., Qiu, L., Shao, M., *et al.* 2012. The Accumulation of organic carbon in mineral soils by afforestation of abandoned farmland. *PLoS ONE* 7, e32054.
336. Wei, Y.C., Ouyang, Z.Y., Miao, H., *et al.* 2007. Spatial heterogeneity of soil properties in Jianfengling Nature Reserve. *Chinese Journal of Ecology*, 26, 197-203. (in Chinese)
337. Wei, Y.M., 2010. Biomass and carbon stock for Large-scale implementation of poplar plantation in desert environment. *Master Dissertation of Xinjiang University*. (in Chinese)
338. Wei, Y.W., Li, M.H., Chen, H., *et al.* 2013. Variation in carbon storage and its distribution by stand age and forest type in boreal and temperate forests in Northeastern China. *PLoS ONE* 8, e72201.
339. Wen, L., Lei, P.F., Dai, L., 2014. Storages and distribution characteristics of soil organic carbon and nitrogen in pure *Cinnamomum camphora* forests at different stand ages. *Journal of Central South University of Forestry & Technology* 34, 106-111. (in Chinese)
340. Wu, G., Jiang, P., Shao, H.B., *et al.* 2007. Nutrients and biomass spatial patterns in alpine tundra ecosystem on Changbai Mountains, Northeast China. *Colloids and surfaces B* 60, 250-256.
341. Wu, J.J., Yang, Z.J., Weng, F.J., *et al.* 2014. Comparison of soil respiration in natural *Castanopsis carlesii* forest and plantation forest. *Environmental Science* 35, 2426-2431.
342. Wu, M., 2009. Characteristics of carbon stocks and stability of soil organic carbon under three artificial forest types in mid-subtropics. *Master Dissertation of Nanjing agricultural university*. (in Chinese)
343. Wu, P., Chen, J., Cui, Y.C., *et al.* 2012. Study of soil organic carbon of major successional communities in Maolan Nature Reserve of Karst. *Journal of Central South University of Forestry & Technology* 32, 181-186. (in Chinese)
344. Wu, P.F., Zhu, B., Liu, S.R., *et al.* 2008. Carbon storage and its allocation in mixed alder-cypress plantations at different age stages. *Chinese Journal of Applied Ecology* 19, 1419-1424. (in Chinese)
345. Wu, T., Peng, C.H., Tian, D.L., *et al.* 2012. Spatial distribution of carbon storage in a 13-year-old *Pinus massoniana* forest ecosystem in Changsha City, China. *Acta Ecologica Sinica* 32, 4034-4042. (in Chinese)
346. Wu, X.C., 2009. Productivity and carbon density of natural poplar and willow forest in Ergis River, Xinjiang Province. *Doctoral Dissertation of Inner Mongolia Agricultural University*. (in Chinese)
347. Wu, X.G., Guo, J.P., Tian, X.P., *et al.* 2014. Distribution characteristics of soil organic carbon and total nitrogen along elevation gradients in Luya Mountain. *Ecology and Environment Sciences* 23, 50-61.
348. Wu, X.L., Cheng, Y.Q., Luo, Y.J., *et al.* 2014. Carbon sequestration and storage of citrus orchard system in three Gorges Reservoir region of Chongqing, Southwest China*. Journal of Agricultural Sciences* 27, 693-698. (in Chinese)
349. Wu, Y.C., Li, Z.C., Cheng, C.F., *et al.* 2013. Effects of understory removal on forest carbon storage in *Cinnamomum camphora* plantation ecosystem. *Chinese Journal of Plant Ecology* 37, 142-149. (in Chinese)
350. Xian, Y., 2014. Vertical distribution patterns of soil organic matter and total nitrogen contents in different altitudes of eastern slope of Gaoligong Mountain. (in Chinese)
351. Xiang, Y.Z., 2012. Spatial-temporal structure of biomass and carbon storage in *Eucalyptus* plantation ecosystem in Hainan Province. *Doctoral Dissertation of China's forestry science research*. (in Chinese)
352. Xiao, C.B., Wang, H., Fan, K.F., *et al.* 2010. Carbon storage of *Metasequoia glyptostroboides* plantation ecosystems at different age Stages in Chongming Island, East China. *Journal of ShangHai JiaoTong University* (Agricultural Science) 28, 30-34. (in Chinese)
353. Xiao, F.M., 2007. Characteristics of carbon balance in *Phyllostachys edulis* plantation ecosystem. *Master Dissertation of the Chinese academy of forestry science*. (in Chinese)
354. Xiao, S.S., 2007. Soil respiration and carbon balance in *Casuarina Equisetifolia* plantation ecosystem on Coastal Sand. *Master Dissertation of Fujian Agriculture and Forestry University*. (in Chinese)
355. Xiao, T., 2006. Carbon storage in tropical seasonal rain forest in Xishuangbanna, Yunnan Province. *Master Dissertation of Chinese academy of sciences*. (in Chinese)
356. Xiao, Z.W., Wang, L.G., Mao, J.M., *et al.* 2012. Carbon storage of different tree-tea agroforestry systems in Xishuangbanna, Yunnan Province of Southwest China. *Chinese Journal of Ecology* 31, 1617-1625. (in Chinese)
357. Xiao, Z.W., Wang, X.H., Zheng, L., *et al.* 2014. Biomass and its allocation pattern of monoculture and mixed rubber-tree plantations in Xishuangbanna. *Journal of Central South University of Forestry & Technology* 34, 108-116. (in Chinese)
358. Xiao, Y.F., 2013. Spatial distribution of soil organic carbon and the analysis of influencing factors in Mangshan Mountain. *Master Dissertation of Central south Forestry University of Science and Technology*. (in Chinese)
359. Xie, J.S., Guo, J.F., Yang, Z.J., *et al.* 2013. Rapid accumulation of carbon on severely eroded red soils through afforestation in subtropical China. *Forest Ecology and Management* 300, 53-59.
360. Xie, R.S., Li, X.W., Chen, C.X., *et al.* 2014. Maturity of Carbon Sink Economy in *Pinus massoniana* forest. *Journal of Shanxi Agricultural University (Natural Science Edition)* 34, 370-374. (in Chinese)
361. Xie, R.S., Chen, C.G., Li, X.W., *et al.* 2014. Carbon storage maturity of *Cunninghamia lanceolata* plantation in Henan Province. *Journal of Southwest Forestry University* 34, 35-38. (in Chinese)
362. Xie, Y.G., 2014. Biomass of *Castanopsis eyrei* community domain spices in Leigongshan Mountain, Guizhou Province. *Guizhou Forestry Science and Technology* 42, 1-6. (in Chinese)
363. Xin, W.J., Su, Y.Q., Zhu, M.Q., *et al.* 2014. Distribution characteristics of soil organic carbon of different forests in loess plateau of Qianyang County. *Journal of Central South Forestry University* 34, 66-671. (in Chinese)
364. Xiong, Y.M., Xia, H.P., Li, Z.A., *et al.* 2007. Impacts of litter and understory removal on soil properties, *Plant and Soil* 304, 179-188.
365. Xu, G.L., Zhang, H.J., Lü, X.H., 2014. The Soil organic carbon storage study under five plantations in the simian mountain of Chongqing. *Ecology and Environment Sciences* 23, 211-216. (in Chinese)
366. Xu, H., Zhang, Y.R., Ji, B., *et al.* 2014. Organic carbon of soil and roots for different woodland of Helanshan Mountain. *Journal of Arid Land Resources and Environment* 28, 162-166. (in Chinese)
367. Xu, L., 2014. Spatial heterogeneity of soil organic carbon distribution in the Korean pine broad-leaved forest of northeast China. *Chinese Journal of Soil Science* 45, 100-104. (in Chinese)
368. Xu, Q.F., Xu, J.M., 2003. Changes in soil carbon pools induced by substitution of plantation for native forest. *Pedosphere* 13, 271-278.
369. Xu, Q.X., 2013. Thinning effect on carbon storage of *Larix gmelini* forest in Daxinganling Mountains. *Master Dissertation of Northeast Forestry University*. (in Chinese)
370. Xu, X.M., 2012. Carbon density of *Populus Tomentosa* plantations at different ages on Loess Plateau. *Master Dissertation of Northwest Agriculture and Forestry University*. (in Chinese)
371. Xu, X.M., Liu, Y., Zhang, L.L., *et al.* 2012. Carbon density in forest ecosystem of old *Populus tomentosa* plantation in Loess Plateau. *Shaanxi Forest science and technology* 9*,* 1-5. (in Chinese)
372. Xuan, Z.L., Zhang, Q.C., Ge, L.L., *et al.* 2013. Biomass structure and distribution of Korean Larch plantations. *Forestry resources management* 1, 53-57. (in Chinese)
373. Xue, L., Xue, Y., Lie, G.W., *et al.* 2012. Soil organic carbon storage on different slope positions in *Cunninghamia Lanceolata* stands. *Bulletin of Soil and Water Conservation* 32, 43-47. (in Chinese)
374. Cao, X.Y., 2013. Carbon sequestration ability of main afforestation tree species in Central and Southern Shanxi Province, China. *Master Dissertation of Beijing Forestry University*. (in Chinese)
375. Yan, D.F., 2012. Collaborative mechanisms of vegetation and soil succession under different vegetation restoration. *Master Dissertation of Henan Agricultural University*. (in Chinese)
376. Yan, E.R., Wang, X.H., Huang, J.J., *et al.* 2007. Long-lasting legacy of forest succession and forest management: Characteristics of coarse woody debries in an evergreen broad-leaved forest of Eastern China. *Forest Ecology and Management* 252, 98-107.
377. Yan, J., Luo, Y.J., Zheng, D.F., *et al.* 2014. Source appointment of differences in biomass estimates of *Eucalypt* Plantation. *Scientia Silvae Sinicae* 50, 92-98. (in Chinese)
378. Yan, P., Feng, X.C., 2006. Spatial distribution and carbon storage in primitive broadleaved Korean Pine Forests. *Journal of Northeast Forestry University* 34, 23-25. (in Chinese)
379. Yang, D., 2014. Spatial heterogeneity of soil organic carbon and total nitrogen concentrations in a *Lithocarpus glaber*-*Cyclobalanopsis glauca* evergreen broadleaved forest. *Acta Ecologica Sinica* 34, 3452-3468. (in Chinese)
380. Yang, F.F., Li, Y.L., Zhou, G.Y., 2010. Dynamics of coarse woody debris and decomposition rates in an old-growth forest in lower tropical China. *Forest Ecology and Management* 259, 1666–1672.
381. Yang, F.P., 2013. The relationship between tree growth and climate change in Huoditang forest region, Qinling Mountains. *Master Dissertation of northwest Agriculture and Forestry University*. (in Chinese)
382. Yang, F.P., Hu Z.Y., Zhang, S.X., 2014. Dynamic changes in biomass and volume of the tree layer of *Pinus tabulaeformis* and *Pinus armandi* forests at different altitudes. *Journal of Northwest A&F University* (Natural Science Edition) 43, 68-76. (in Chinese)
383. Yang, F.X., 2012. Carbon Stock and its allocation in broadleaf-coniferous mixed forest in Baotianman Nature Reserve. *Master Dissertation of Henan Agricultural University*. (in Chinese)
384. Yang, H.S., Yuan, Y.G., Zhang, Q., *et al.* 2011. Changes in soil organic carbon, total nitrogen, and abundance of *Arbuscular mycorrhizal* fungi along a large-scale aridity gradient. *Catena* 87, 70-77.
385. Yang, J., 2013. Effect of forest closed on biomass and carbon storage in several kinds of stands. *Master Dissertation of Central China Agricultural University*. (in Chinese)
386. Yang, J.J., 2012. Carbon storage in *Pinus tabulaeformis* plantations at different forest ages in Pingquan area, Hebei Province. *Master Dissertation of Beijing Forestry University*. (in Chinese)
387. Yang, J.Y., Wang, C.K., 2005. Soil carbon storage and flux of temperate forest ecosystems in northeastern China. *Acta Ecologica Sinica* 25, 2875-2882. (in Chinese)
388. Yang, K., Shi, W., Zhu, J.J., 2013. Impact of secondary forest conversion into larch plantations on soil chemical and microbiological properties. *Plant and Soil* 368, 535-546.
389. Yang, L., Pan, J., Shao, Y., *et al.* 2007. Soil organic carbon decomposition and carbon pools in temperate and sub-tropical forests in China. *Journal of Environmental Management* 85, 690-695.
390. Yang, L.L., Zhang, F.S., Mao, R.Z., *et al.* 2008. Conversion of natural ecosystems to cropland increases the soil net nitrogen mineralization and nitrification in Tibet. *Pedosphere* 18, 699-706.
391. Yang, L.P., liu, W.Y., Yang, G.P., *et al.* 2007. Composition and carbon storage of woody debris in moist evergreen broad-leaved forest and its secondary forests in Ailaoshan Mountains of Yunnan Province. *Chinese Journal of Applied Ecology* 18, 2153-2159. (in Chinese)
392. Yang, L.Y., Wu, S.T., Zhang, L.B., 2010. Fine root biomass dynamics and carbon storage along a successional gradient in Changbai Mountains, China. *Forestry* 83, 379-387.
393. Yang, X.F., 2011. Carbon storage and the environment response of poplar plantation in Xiping County, Henan Province. *Master Dissertation of Beijing Forestry University*. (in Chinese)
394. Yang, X.J., Wang, H.Y., Liu, L., *et al.* 2013. Soil fertility in Korean Larch plantations of different stand age. *Journal of Northeast Forestry University* 41, 51-56. (in Chinese)
395. Yang, X.M., 2010. Carbon storage and density features of natural forest of *Pinus tabulaeformis fshekannesis* in Loess Plateau. *Master Dissertation of northwest Agriculture and Forestry University*. (in Chinese)
396. Yang, X.Q., Yuan, H.Y.Z., 2014. Spatial variations of soil organic carbon and nitrogen of forestland in Guandi Mountain. *Forest Research* 24, 223-229. (in Chinese)
397. Yang, Y., Ran, F., Wang, G.X., *et al.* 2013. Biomass model and carbon storage of *Pinus yunnanensis* on Tibet Plateau of China. *Chinese Journal of Ecology* 32, 1674-1682.
398. Yang, Y., Wang, G.X., Shen, H.H., *et al.* 2014. Dynamics of carbon and nitrogen accumulation and C:N stoichiometry in a deciduous broadleaf forest of deglaciated terrain in the eastern Tibetan Plateau. *Forest Ecology and Management* 312, 10-18.
399. Yang, Y.S., Guo, J.F., Lin, P., 2004. Carbon and nutrient pools of forest floor in native forest and monoculture plantations in subtropical China. *Acta Ecologica Sinica* 24, 359-367. (in Chinese)
400. Yang, Y.S., Guo, J.F., Lin, P., *et al.* 2005. Carbon and nutrient pools of coarse woody debris in a natural forest and plantation in subtropical China. *Scientia Silvae Sinicae* 41, 7-11. (in Chinese)
401. Yang, F.F., 2011. Coarse woody debris in monsoon evergreen broad-leaved forest in Dinghu Mountain. *Master Dissertation of Garden of South China botany, Chinese academy of sciences*. (in Chinese)
402. Yang, Z.J., 2007. Carbon sequestration and balance in pure plantations of *Cunninghamia Lanceolata* and *Schima Superba*. *Master Dissertation of Fujian Agriculture and Forestry University*. (in Chinese)
403. Ye, G.F., Zhang, Q.H., Lu, C.Y., Lin, Y.M., 2007. Litter and Caloric Value of Casuarina equisetifolia Plantation in Coastal Sandy Area. *Chinese Journal Appllied Environmental Biology* 13, 23-28.
404. Ye, S.M., Long, T., Lan, J.X., *et al.* 2010. Carbon storage and spatial distributions characteristic of stratified mixed stands of *Eucalyptus urophylla* and *Acacia mangium*. *Acta Agriculturae Universitatis Jiangxiensis*(Natural Sciences Edition) 32, 735-742. (in Chinese)
405. You, W.Z., Huo, C.F., Xing, Z.K., *et al.* 2011. Biomass and Net Primary Productivity of *Larix olgensis* plantation in Bingla Mountains, Northeast China. *Journal of Shenyang Agricultural University* 42, 565-569. (in Chinese)
406. Yu, H.Q., He, G.M., Zhang, F., *et al.* 2012. Forest carbon storage in Fengjiayu town, Miyun County, Beijing. *Forest Resources Management* 1, 37-42. (in Chinese)
407. Yu, S.B., Wang, D., Dai, W., *et al.* 2014. Soil carbon budget in different-aged Chinese fir plantations in south China. *Journal of Forestry Research* 25, 621-626.
408. Yue, C.R., 2011. Forest biomass estimation on Shangri-La County based on remote sensing. *Doctoral Dissertation of Beijing Forestry University*. (in Chinese)
409. Ai, Z.M., Chen, Y.M., Cao, Y., 2014. Storage and allocation of carbon and nitrogen in *Robinia pseudoacacia* plantation at different ages in the loess hilly region, China. *Chinese Journal of Applied Ecology* 25, 333-341.
410. Zeng, X.H., Zhang, W.J., Cao, J.S., *et al.* 2014. Changes in soil organic carbon, nitrogen, phosphorus, and bulk density after afforestation of the"Beijing-Tianjin Sandstorm Source Control"program in China. *Catena* 118, 186-194.
411. Zeng, X.H., Zhang, W.J., Liu, X.P., *et al.* 2014. Change of soil organic carbon after cropland afforestation in Beijing-Tianjin sandstorm source control’s program area in China. *Chinese Geographical Science* 24, 461-470.
412. Zeng, X.H., Zhang, W.J., Liu, X.P., *et al.* 2014. Change of soil organic carbon after cropland afforestation in Beijing-Tianjin sandstorm source control's program area in China. *Chinese Geographical Science* 24, 461-470. (in Chinese)
413. Zeng, X.P., 2007. Productivities and structural functions of three plantation community in Heshan Mountain. *Master Dissertation of Garden of South China botany, Chinese academy of sciences*. (in Chinese)
414. Zeng, Z.Q., 2012. Subtropical evergreen broad-leaved forest carbon density and carbon sequestration potential in different succession stage. *Doctoral Dissertation of Chinese academy of sciences*. (in Chinese)
415. Zeng, Z.Q., Wang, S.L., Zhang, C.M., *et al.* 2013. Carbon storage in evergreen broad-leaf forests in mid-subtropical region of China at four succession stages. *Journal of Forestry Research* 24, 677-682.
416. Zha, T.G., 2007. Carbon balance of a poplar plantation ecosystem in Daxing, Beijing. *Doctoral Dissertation of Beijing Forestry University*. (in Chinese)
417. Zhan, Z.Q., 2011. Study on biomass and carbon storage of moso bamboo in Sheshan area, Shanghai. Master *Dissertation of Shanghai Jiaotong University*. (in Chinese)
418. Zhang, C.B., Huang, L.N., Wong, M.H., *et al.* 2006. Characterization of soil physico-chemical and microbial parameters after revegetation near Shaoguan Pb/Zn smelter, Guangdong, P.R. China. *Water, Air, and Soil Pollution* 177, 81-101.
419. Zhang, C.B., Huang, L.N., Shua, W.S., *et al.* 2007. Structural and functional diversity of a culturable bacterial community during the early stages of revegetation near a Pb/Zn smelter in Guangdong, PR China. *Ecological Engineering* 30, 16-26.
420. Zhang, D.M., 2012. Study on carbon budget of desert shrubs in Alashan desert region. *Master Dissertation of Inner Mongolia Agricultural University.* (in Chinese)
421. Zhang, G.Q., 2008. The Impact on carbon stocks of artificial pine forest ecosystems with different management. *Master Dissertation of Sichuan agricultural university*. (in Chinese)
422. Zhang, H., 2010. Study on the dominant tree biomass and carbon storage in Daqing Mountian. *Master Dissertation of Inner Mongolia Agricultural University.* (in Chinese)
423. Zhang, H., Song, T.Q., Ke, L., *et al.* 2013. Oak biomass and its allocation at different stand ages in West of Guangxi, China. *Research of Agricultural Modernization* 34, 758-762. (in Chinese)
424. Zhang, H., Guan, D.S., Song, M.W., 2012. Biomass and carbon storage of *Eucalyptusand Acacia* plantations in the Pearl River Delta, South China. *Forest Ecology and Management* 227, 90-97.
425. Zhang, J., 2008. Study on community structure, biomass and carbon storage of *Larix Gmelini* plantation. *Master Dissertation of Beijing Forestry University*. (in Chinese）
426. Zhang, J., Wang, S.L., Feng, Z.W., *et al.* 2009. Stability of soil organic carbon changes in successive rotations of Chinese fir (*Cunninghamia lanceolata* (Lamb.) Hook) plantations. *Journal of Environmental Sciences* 21, 352–359.
427. Zhang, J., Ge, Y., Chang, J., *et al.* 2007. Carbon storage by ecological service forests in Zhejiang Province, subtropical China. *Forest Ecology and Management* 245, 64-75.
428. Zhang, J.B., Song, C.C., Wang, S.M., 2007. Dynamics of soil organic carbon and its fractions after abandonment of cultivated wetlands in northeast China. *Soil&Tillage Research* 96, 350-360.
429. Zhang, J.J., Li, Y.F., Scott, X., *et al.* 2014. Understory vegetation management affected greenhouse gas emissions and labile organic carbon pools in an intensively managed Chinese chestnut plantation. *Plant and Soil* 376, 363-375.
430. Zhang, J.Q., Su, Y.Q., Kang, Y.X., *et al.* 2009. Carbon sequestration of young *Robinia pseudoacacia* plantation in Loess Plateau. *Chinese Journal of Applied Ecology* 20, 2911-2916. (in Chinese)
431. Zhang, K., Xu, X.N., Wang, Q., *et al.* 2010. Biomass, and carbon and nitrogen pools in a subtropical evergreen broad-leaved forest in eastern China. *Journal of Forest Research* 15, 274-282.
432. Zhang, L.L., Su, Y.Q., Liu, Y., *et al.* 2013. Study on organic carbon of *Platycladus orientalis* plantation in Qianyang loess plateau. *Journal of Central South University of Forestry* 33, 56-60. (in Chinese)
433. Zhang, M., Liang, W.J., Jiang, Y., *et al.* 2011. Distribution of soil organic carbon fractions along the altitudinal gradient in Changbai Mountain, China. *Pedosphere* 21, 615-620.
434. Zhang, N.Y., Zhang, J.Q., Yang, Y.X., *et al.* 2009. Impact of different artificial ecological forests on soil nutrients in Loess Plateau. *Journal of Northeast Forestry University* 37, 44-46. (in Chinese)
435. Zhang, P., 2011. Study on forest carbon stock in Beijing of China. *Doctoral Dissertation of Beijing Forestry University*. (in Chinese)
436. Zhang, Q.L., Wang, F., Li, X.M., *et al.* 2013. Storage and composition of coarse woody debris in natural *Sphagnum bryum*-*Larix gmelinii* forests of Daxinganling Mountains. *Ecology and Environment Sciences* 22, 437-442. (in Chinese)
437. Zhang, Q.Z., Wang, C.K., 2010. Carbon density and distribution of six Chinese temperate forests. *Science China: Life Sciences* 7, 831-840.
438. Zhang, Q.Z., Wang, C.K., Wang, X.C., *et al.* 2009. Carbon concentration variability of 10 Chinese temperate tree species. *Forest Ecology and Management* 258, 722-727.
439. Zhang, S.B., 2014. Biomass partitioning affects the growth of *Pinus* species from different elevations. *Plant Diversity and Resources* 36, 47-55. (in Chinese)
440. Zhang, S.L., Zhang, X.Y., Liu, Z.H., *et al.* 2014. Spatial heterogeneity of soil organic matter and soil total nitrogen in a Mollisol watershed of Northeast China. *Environment Earth Science* 72, 275-288.
441. Zhang, S.R., Li, T., Xu, X.X., *et al.* 2012. Soil carbon fractions of restored lands in Liusha river valley, Sichuan Province. *Ecological Engineering* 40, 27-36.
442. Zhang, T.T., 2012. Biomass and carbon storage of *Larix principis-rupprechtii* forest plantation. *Master Dissertation of Beijing Forestry University*. (in Chinese)
443. Zhang, W.G., Xie, W.D., Jiang, G.X., *et al.* 2011. Effects of age structure of *Juglans regia* young forest in rocky desertification area on its carbon stock. *Journal of Central South University of Forestry* 31, 96-101. （in Chinese）
444. Zhang, W.J., Liao, H.K., Long, J., *et al.* 2014. Effects of land use on soil organic carbon and its turnover rate in Karst mountain areas of Guizhou Province. *Chinese Journal of Ecology* 33, 1297-1303.
445. Zhang, X.H., Fan, Z.P., Sun, X.K., *et al.* 2009. Effects of land use change on ecosystem carbon stock in semi-arid region. *Chinese Journal of Ecology* 28, 2424-2430.
446. Zhang, X.L., Wang, S., Wang, X., *et al.* 2014. Soil organic carbon storage of Diaoluoshan Natural Reserve in Hainan. Chinese *Journal of Tropical Crops* 35, 362-368.
447. Zhang, X.P., Wang, M.B., Liang, X.M., 2009. Quantitative classification and carbon density of the forest vegetation in Lüliang Mountains of China. *Plant Ecology* 201, 1-9.
448. Zhang, X.S., Wang, B., Feng, W.F., 2013. Spatial patterns of soil organic carbon in oak and pine-oak mixed forests in Jigong Mountain in temperate-subtropical ecotone. *Journal of Anhui Agricultural University* 40, 18-22. (in Chinese)
449. Zhang, X.Y., Guan, D.S., Li, H.S., *et al.* 2009. Allocation Characteristics of Organic Carbon Pool in Typical Forest Soils in Guangzhou. *Journal of Sun Yatsen University* 48, 137-141. (in Chinese)
450. Zhang, X.Y., Xu, Z.C., Zeng, F.T., *et al.* 2011. Carbon density distribution and storage dynamics of forest ecosystem in Pearl River Delta of low subtropical China. *China Environmental Science* 31, 69-77. (in Chinese)
451. Zhang, Y.T., Hu, S.S., Li, J.M., *et al.* 2013. Characteristic of root biomass of three main forest types in Xinjiang. *Arid Land Geography* 36, 269-276. (in Chinese)
452. Zhang, Z., Zhong, Q.L., Cheng, D.L., *et al.* 2014.The Structure characteristics of carbon storage of ecosystem of ever-green broad-leaved mixed forest with different forest ages in the north-west of Fujian province. *Ecology and Environment Sciences* 23, 203-210. (in Chinese)
453. Zhang, Z.H., Lu, G.Q., Yuan, Z.G., *et al.* 2005. Estimation of carbon cycling of natural secondary forest in Taihang Mountain. *Hebei Journal of Forestry and Orchard Research* 2, 11-13. (in Chinese)
454. Zhang, Z.H., Wang, L.C., Zheng, D.G., *et al.* 2011. Study on artificial forest stand biomass of *Pinus yunnanensis faranch* in Northwest Yunnan Province. *Journal of Anhui Agricultural Sciences* 39, 19203-19205. (in Chinese)
455. Zhang, Z.J., Zhang, X.Q., Wang, Y.H., 2009. Carbon storage and distribution of *Pinus massoniana* forest ecosystem in Tieshanping of Chongqing. *Scientia Silvae Sinicae* 45, 49-53. (in Chinese)
456. Zhang, Z.Q., Wang, C.K., 2010. Carbon density and distribution of six Chinese temperate forests. *Science China Life Science* 40, 621-631. (in Chinese)
457. Zhang, X.Q., Kirschbaum M.U.F., Houa Z.H., *et al.* 2004. Carbon stock changes in successive rotations of Chinese fir *(Cunninghamia lanceolata* (lamb) hook) plantations. *Forest Ecology and Management* 202, 131-147.
458. Zhao, K., 2010. Study on the carbon storage in pure and mixture plantations *Fokienia Hodginsi* and *Michelia macclurei*. *Master Dissertation of Fujian agriculture and Forestry University*. (in Chinese)
459. Zhao, M.J., Chen, Y.M., Ai, Z.M., *et al.* 2013. Distribution characteristics of soil organic carbon and their affecting factors of typical plantations in Loess Hilly Region. *Bulletin of Soil and Water Conservation* 33, 270-275. (in Chinese)
460. Zhao, M.Q., Shi, Y.F., Storage, C.B., *et al.* 2014. Distribution in mango plantation ecosystems in Sanya. *Journal of Anhui Agricultural Sciences* 42, 1088-1090. (in Chinese)
461. Zhao, N., Meng, P., Zhang, J.S., *et al.* 2014. Comparison of soil respiration under various land uses in hilly area of Northern China. *Scientia Silvae Sinicaes* 50, 1-7. (in Chinese)
462. Zhao, Y., Fan, W., Wu, M.Z., *et al.* 2009. The nutrient allocation and cycling pattern in *Platycladus orientalis* plantation in hilly region of Taihang Mountains. *Journal of Soil and Water Conservation* 23, 143-152. (in Chinese)
463. Zhao, Y., Wu, M.Z., Fan, W., *et al.* 2009. Comparison of nutrient return and litter decomposition between coniferous and broad-leaved forests in hilly region of Taihang Mountains. *Journal of Natural Resources* 24, 1616-16240. (in Chinese)
464. Zhao, Z.Y., Shu, Q.T., Du, A.P., 2013. Study on water holding capacity of litter and soil in *Eucalyptus urophylla* plantations. *Journal of Central South University of Forestry* 33, 98-102. (in Chinese)
465. Zheng, J.P., Guo, Z.L., Xu, C.Y., *et al.* 2011. Seasonal dynamics of litter accumulation in major forest communities on the northern slope of Changbai Mountain, Northeast China. *Acta Ecologica Sinica* 31, 4299-4307. (in Chinese)
466. Zheng, L.Y., Wang, X.T., Zhang, J.L., 2013. Characteristics of the carbon density of main forest types in Northwestern Hubei. *Hubei Forestry science and technology* 2, 1-7. (in Chinese)
467. Zheng, Z., Liu, H.M., Feng, Z.L., 2006. Biomass of tropical montane rain forest in Xishuangbanna of Southwest China. *Chinese Journal of Ecology* 25, 347-353. (in Chinese)
468. Zhong, X.F., 2007. Effect of continuous planting of FIR plantation on soil carbon storage and dissolved carbon storage, Fujian Province. *Master Dissertation of Fujian Normal University.* (in Chinese)
469. Zhong, Y.X., Zhou, Y.C., Li, Z.J., 2014. Research on the carbon ttorage and potential carbon sequestration of vegetation in the trough valley of a Karst area, Yinjiang. *Earth and Environment* 42, (1). (in Chinese)
470. Zhou, L.L., Wu, P.F., Li, S.B., *et al.* 2014. Litter fall production and nutrient return in different-aged Chinese fir (*Cunninghamia lanceolata*) plantations in South China. *Journal of Forestry Research* 25, 1-10.
471. Zhou, P., Zhu, W.Z., Luo, J., *et al.* 2013. Aboveground biomass and carbon storage of typical forest types in Gongga Mountain. *Acta Botanica Boreali-Occidentalia Sinica* 33, 162-168. (in Chinese)
472. Zhou, Q.Q., 2012. Study on biomass and carbon storage of *Pinus masson* forest in Yongchunmulin Natural Reserve, Fujian Province. *Master Dissertation of Fujian Agriculture and Forestry University*. (in Chinese)
473. Zhou, Y., Su, J.Q., Janssens I.A., *et al*. 2014. Fine root and litter fall dynamics of three Korean pine (*Pinus koraiensis*) forests along an altitudinal gradient. *Plant and Soil* 374, 19–32.
474. Zhou, Z.Y., Wang, X.P., Zheng, J.M., *et al.* 2012. Estimation methods in predicting carbon stock of forest communities in the natural reserve of Baihua Mountain. *Journal of Northwest A&F University* (Natural Science Edition) 40, 139-146. (in Chinese)
475. Zhu, G.Q., 2007. Research on soil respiration dynamics and fine root patterns for four hybrid poplar (*Populus*×*Euramericana*) plantation in the northern areas of Jiangsu Province. *Master Dissertation of Nanjing Forestry University*. (in Chinese)
476. Zhu, H.H., He, X.Y., Wang, K.L., *et al.* 2012. Interactions of vegetation succession, soil bio-chemical properties and microbial communities in a Karst ecosystem. *European Journal of Soil Biology* 51, 1-7.
477. Zhu, J.Y., Mo, L.J., Ye, Y.C., *et al.* 2011. Study on Carbon Storage of the Forest Ecosystem in Dongguan. *Guangdong Forestry science and technology* 27, 22-29. (in Chinese)
478. Zhu, L.Y., Pan, J.J., Zhang, W., 2013. Study on soil organic carbon pools and turnover characteristics along an elevation gradient in Qilian Mountain. *Environmental Science* 34, 668-675. (in Chinese)
479. Zhu, S.Y., 2009. Study on the forest characteristics in the process of secondary succession in the northwest Karst area, West of Guizhou Province. *Master Dissertation of the Chinese Academy of Sciences*. (in Chinese)
480. Zhu, Y.F., 2013. Study on above-ground carbon storage and energy of *Quercus acutissima* forest in Northern subtropical area. *Master Dissertation of Nanjing Forestry University*. (in Chinese)
481. Zhu, Y.F., Hu, H.B., Xu, N., *et al.* The distributing characteristics of aboveground biomass and carbon storage of *Quercus acutissima* plantation with different ages. *China Forestry Science and Technology* 28, 20-24. (in Chinese)
482. Zhuang, H.L., 2012. Study on carbon dynamics of *Metasequoia glyptostroboides* plantation ecosystems in Chongming Island, Shanghai. *Master Dissertation of Shanghai Jiaotong University*. (in Chinese)
483. Zhang, J.Q., Xu, X.M., Wang, X.F., *et al.* 2011. Carbon sink in artificial forest ecosystem of *Robina* and *Pinus* in Loess Plateau. *Arid Land Geography* 34, 201-207. (in Chinese)
484. Zhang, X.Y., Meng, X.J., Gao, L.P., et al. 2010. Potential impacts of climate warming on active soil organic carbon contents along natural altitudinal forest transect of Changbai Mountain. *Acta Ecological Sinica* 30, 113-117. (in Chinese)
485. Zhang, Y., 2012. Forest biomass carbon estimated by remote sensing in eight Angle Mountain Nature Reserve. *Master Dissertation of Tianjin Normal University*. (in Chinese)

**Appendix S3:** Site information and carbon density of above ground biomass (AGC) from 259 studies published between 2004 and 2014 (See dataset).

**Appendix S4:** Site information and carbon density of below ground biomass (BGC) from 259 studies published between 2004 and 2014 (See dataset).

**Appendix S5:** Site information and the related carbon density of dead mass (DMC) from 180 studies published between 2004 and 2014 (See dataset).

**Appendix S6:** Site information and the related soil organic carbon density in the 0-100 cm soil layer (SOC) from 187 studies published between 2004 and 2014 (See dataset).

**Appendix S7:** The relationships were between the measured and predicted values of SOC density (SOCD, Mg/ha) in the 0–100 cm soil layer. The power function was used to fit the 0–100 cm SOC from soil surface data, where the power functions were derived from Chai et al (2015).


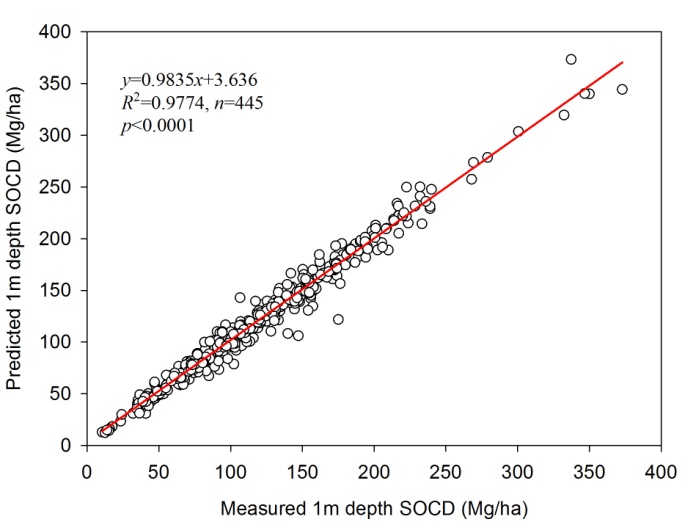


Appendix S7. The relationships were between the measured and predicted values of SOC density (SOCD, Mg/ha) in the 0–100 cm soil layer. The power function was used to fit the 0–100 cm SOC from soil surface data, where the power functions were derived from Chai et al (2015).

**Appendix S8:** Carbon density and storage were estimated by 6 forest type groups in China’s forest ecosystems

| Code | 6 forest type groups ^†^ | Carbon density (Mg C ha^–1^) | | | | | | | | | |
| --- | --- | --- | --- | --- | --- | --- | --- | --- | --- | --- | --- |
|  |  | AGC^†^ | | BGC | | DMC | | SOC | | Ecosystem | |
|  |  | Mean | SE | Mean | SE | Mean | SE | Mean | SE | Mean | SE |
| 1 | Cold and temperate coniferous forests | 59.75 | 2.01 | 13.09 | 0.52 | 8.76 | 0.64 | 194.23 | 5.13 | 275.83 | 5.57 |
| 2 | Coniferous mixed broadleaf forests | 70.78 | 5.17 | 14.89 | 1.19 | 10.05 | 0.98 | 189.98 | 12.06 | 285.70 | 13.21 |
| 3 | Deciduous broadleaf forest | 38.83 | 1.38 | 10.31 | 0.39 | 2.64 | 0.19 | 116.57 | 4.67 | 168.34 | 4.89 |
| 4 | Temperate Coniferous Forests | 38.59 | 2.13 | 10.54 | 0.86 | 5.65 | 0.75 | 99.19 | 4.78 | 153.98 | 5.36 |
| 5 | Warm Coniferous Forests | 54.05 | 1.81 | 10.29 | 0.32 | 3.76 | 0.21 | 119.93 | 2.22 | 188.02 | 2.89 |
| 6 | Evergreen broadleaf forests | 68.27 | 2.66 | 15.05 | 0.65 | 3.37 | 0.17 | 134.69 | 3.38 | 221.41 | 4.35 |
|  | Total | 52.99 | 1.89 | 11.59 | 0.45 | 4.48 | 0.31 | 136.11 | 3.92 | 204.47 | 4.45 |

**Appendix S8:** Carbon density and storage were estimated by 6 forest type groups in China’s forest ecosystems (Continued)

| Code | 6 forest type groups | Area  (10^8^ha) | Carbon storage (Pg C) | | | | | | | | | |
| --- | --- | --- | --- | --- | --- | --- | --- | --- | --- | --- | --- | --- |
|  |  |  | AGC | | BGC | | DMC | | SOC | | Ecosystem | |
|  |  |  | Mean | SE | Mean | SE | Mean | SE | Mean | SE | Mean | SE |
| 1 | Cold and temperate coniferous forests | 0.3015 | 1.801 | 0.061 | 0.395 | 0.016 | 0.264 | 0.019 | 5.855 | 0.155 | 8.316 | 0.168 |
| 2 | Coniferous mixed broadleaf forests | 0.0191 | 0.135 | 0.010 | 0.028 | 0.002 | 0.019 | 0.002 | 0.362 | 0.023 | 0.544 | 0.025 |
| 3 | Deciduous broadleaf forest | 0.4704 | 1.826 | 0.065 | 0.485 | 0.018 | 0.124 | 0.009 | 5.483 | 0.220 | 7.918 | 0.230 |
| 4 | Temperate Coniferous Forests | 0.0389 | 0.150 | 0.008 | 0.041 | 0.003 | 0.022 | 0.003 | 0.386 | 0.019 | 0.599 | 0.021 |
| 5 | Warm Coniferous Forests | 0.4702 | 2.542 | 0.085 | 0.483 | 0.015 | 0.177 | 0.010 | 5.640 | 0.104 | 8.841 | 0.136 |
| 6 | Evergreen broadleaf forests | 0.2154 | 1.471 | 0.057 | 0.324 | 0.014 | 0.073 | 0.004 | 2.902 | 0.073 | 4.769 | 0.094 |
|  | Total | 1.5155 | 7.925 | 0.286 | 1.756 | 0.069 | 0.678 | 0.047 | 20.628 | 0.593 | 30.987 | 0.674 |

† AGC, above ground vegetation biomass carbon density; BGC, below ground vegetation biomass carbon density, DMC, dead mass carbon density, SOC, soil organic carbon density in the 0-100cm soil layer.

**Appendix S9:** Carbon density and storage were estimated by 16 forest types in China’s forest ecosystems

| Code | Vegetation type ^†^ | Carbon density (Mg C ha^–1^) | | | | | | | | | |
| --- | --- | --- | --- | --- | --- | --- | --- | --- | --- | --- | --- |
|  |  | AGC^†^ | | BGC | | DMC | | SOC | | Ecosystem | |
|  |  | Mean | SE | Mean | SE | Mean | SE | Mean | SE | Mean | SE |
| 1 | Coniferous forests in cold-temperate zone and on mountains  in temperate zone | 51.86 | 1.83 | 12.01 | 0.54 | 8.86 | 0.65 | 190.26 | 5.18 | 262.99 | 5.55 |
| 2 | Deciduous broadleaf forests in temperate zone | 37.34 | 1.49 | 10.17 | 0.46 | 2.82 | 0.23 | 109.38 | 5.15 | 159.71 | 5.39 |
| 3 | Deciduous microphyllous woodland in temperate zone | 17.91 | 3.43 | 4.20 | 0.90 | 2.46 | 0.21 | 19.16 | 6.32 | 43.73 | 7.25 |
| 4 | Coniferous mixed deciduous broadleaf forests in temperate zone | 70.28 | 5.43 | 15.92 | 1.24 | 10.67 | 1.01 | 192.75 | 12.88 | 289.63 | 14.07 |
| 5 | Coniferous forests in temperate zone | 38.59 | 2.13 | 10.54 | 0.86 | 5.65 | 0.75 | 99.19 | 4.78 | 153.98 | 5.36 |
| 6 | Deciduous broadleaf forests in subtropical zone | 51.08 | 3.49 | 11.74 | 0.81 | 1.96 | 0.22 | 159.69 | 9.20 | 224.46 | 9.87 |
| 7 | Evergreen broadleaf and deciduous mixed forests in subtropical zone | 55.59 | 15.01 | 10.22 | 1.43 | 3.52 | 0.27 | 206.83 | 20.92 | 276.16 | 25.79 |
| 8 | Evergreen broadleaf forests in subtropical zone | 73.16 | 3.85 | 16.53 | 1.00 | 3.60 | 0.33 | 156.92 | 5.64 | 250.20 | 6.91 |
| 9 | Sclerophyllus broadleaf evergreen forests in subtropical zone | 74.36 | 0.52 | 22.95 | 0.65 | 3.78 | 0.47 | 107.86 | 2.27 | 208.95 | 2.47 |
| 10 | Fast grown evergreen broadleaf plantation forest in subtropical zones | 53.93 | 3.79 | 9.65 | 0.75 | 2.79 | 0.21 | 106.52 | 6.23 | 172.89 | 7.33 |
| 11 | Coniferous forests on mountains in subtropical in tropical zones | 105.25 | 6.23 | 19.80 | 1.40 | 6.90 | 1.57 | 240.72 | 19.57 | 372.68 | 20.65 |
| 12 | Coniferous, evergreen, and deciduous broadleaf mixed forests on mountains in subtropical zone | 80.58 | 18.06 | 10.24 | 3.17 | 2.27 | 0.32 | 164.12 | 6.48 | 257.21 | 19.45 |
| 13 | Coniferous forests in subtropical an tropical zones | 54.05 | 1.81 | 10.28 | 0.32 | 3.76 | 0.21 | 119.93 | 2.22 | 188.02 | 2.89 |
| 14 | Bamboo forests and scrubs in subtropical and tropical zones | 18.16 | 2.91 | 9.33 | 1.71 | 2.93 | 0.65 | 142.42 | 6.96 | 172.83 | 7.77 |
| 15 | Tropical monsoon rain forests | 125.70 | 5.37 | 28.33 | 2.70 | 6.79 | 1.10 | 95.93 | 9.50 | 256.75 | 11.29 |
| 16 | Tropical rain forests | 102.69 | 8.79 | 22.99 | 2.16 | 4.20 | 0.48 | 123.54 | 5.40 | 253.43 | 10.55 |
|  | Total | 55.00 | 2.71 | 12.13 | 0.67 | 4.38 | 0.44 | 139.14 | 6.04 | 210.65 | 6.78 |

† AGC, above ground vegetation biomass carbon density; BGC, below ground vegetation biomass carbon density, DMC, dead mass carbon density, SOC, soil organic carbon density in the 0-100cm soil layer.

**Appendix S9:** Carbon density and storage were estimated by 16 forest types in China’s forest ecosystems (Continued)

| Code | Vegetation type | Area  (10^8^ha) | Carbon storage (Pg C) | | | | | | | | | |
| --- | --- | --- | --- | --- | --- | --- | --- | --- | --- | --- | --- | --- |
|  |  |  | AGC | | BGC | | DMC | | SOC | | Ecosystem | |
|  |  |  | Mean | SE | Mean | SE | Mean | SE | Mean | SE | Mean | SE |
| 1 | Coniferous forests in cold-temperate zone and on mountains  in temperate zone | 0.1600 | 0.830 | 0.029 | 0.192 | 0.009 | 0.142 | 0.010 | 3.045 | 0.083 | 4.209 | 0.089 |
| 2 | Deciduous broadleaf forests in temperate zone | 0.4013 | 1.499 | 0.060 | 0.408 | 0.018 | 0.113 | 0.009 | 4.390 | 0.207 | 6.409 | 0.216 |
| 3 | Deciduous microphyllous woodland in temperate zone | 0.0328 | 0.059 | 0.011 | 0.014 | 0.003 | 0.008 | 0.001 | 0.063 | 0.021 | 0.143 | 0.024 |
| 4 | Coniferous mixed deciduous broadleaf forests in temperate zone | 0.0169 | 0.119 | 0.009 | 0.027 | 0.002 | 0.018 | 0.002 | 0.326 | 0.022 | 0.489 | 0.024 |
| 5 | Coniferous forests in temperate zone | 0.0389 | 0.150 | 0.008 | 0.041 | 0.003 | 0.022 | 0.003 | 0.386 | 0.019 | 0.599 | 0.021 |
| 6 | Deciduous broadleaf forests in subtropical zone | 0.0363 | 0.185 | 0.013 | 0.043 | 0.003 | 0.007 | 0.001 | 0.579 | 0.033 | 0.814 | 0.036 |
| 7 | Evergreen broadleaf and deciduous mixed forests in subtropical zone | 0.0171 | 0.095 | 0.026 | 0.017 | 0.002 | 0.006 | 0.000 | 0.353 | 0.036 | 0.472 | 0.044 |
| 8 | Evergreen broadleaf forests in subtropical zone | 0.1204 | 0.881 | 0.046 | 0.199 | 0.012 | 0.043 | 0.004 | 1.890 | 0.068 | 3.013 | 0.083 |
| 9 | Sclerophyllus broadleaf evergreen forests in subtropical zone | 0.0157 | 0.117 | 0.001 | 0.036 | 0.001 | 0.006 | 0.001 | 0.170 | 0.004 | 0.329 | 0.004 |
| 10 | Fast grown evergreen broadleaf plantation forest in subtropical zones | 0.0044 | 0.024 | 0.002 | 0.004 | 0.000 | 0.001 | 0.000 | 0.047 | 0.003 | 0.076 | 0.003 |
| 11 | Coniferous forests on mountains in subtropical in tropical zones | 0.1414 | 1.489 | 0.088 | 0.280 | 0.020 | 0.098 | 0.022 | 3.405 | 0.277 | 5.271 | 0.292 |
| 12 | Coniferous, evergreen, and deciduous broadleaf mixed forests on mountains in subtropical zone | 0.0021 | 0.017 | 0.004 | 0.002 | 0.001 | 0.000 | 0.000 | 0.035 | 0.001 | 0.055 | 0.004 |
| 13 | Coniferous forests in subtropical an tropical zones | 0.4702 | 2.542 | 0.085 | 0.483 | 0.015 | 0.177 | 0.010 | 5.640 | 0.104 | 8.841 | 0.136 |
| 14 | Bamboo forests and scrubs in subtropical and tropical zones | 0.0329 | 0.060 | 0.010 | 0.031 | 0.006 | 0.010 | 0.002 | 0.469 | 0.023 | 0.569 | 0.026 |
| 15 | Tropical monsoon rain forests | 0.0062 | 0.078 | 0.003 | 0.018 | 0.002 | 0.004 | 0.001 | 0.059 | 0.006 | 0.159 | 0.007 |
| 16 | Tropical rain forests | 0.0186 | 0.191 | 0.016 | 0.043 | 0.004 | 0.008 | 0.001 | 0.230 | 0.010 | 0.472 | 0.020 |
|  | Total | 1.5155 | 8.335 | 0.411 | 1.838 | 0.101 | 0.663 | 0.067 | 21.086 | 0.916 | 31.923 | 1.028 |

**Appendix S10:** Carbon density and storage were estimated by 38 forest subtypes in China’s forest ecosystems

| Code | Vegetation type ^†^ | Carbon density (Mg C ha^–1^) | | | | | | | | | |
| --- | --- | --- | --- | --- | --- | --- | --- | --- | --- | --- | --- |
|  |  | AGC^†^ | | BGC | | DMC | | SOC | | Ecosystem | |
|  |  | Mean | SE | Mean | SE | Mean | SE | Mean | SE | Mean | SE |
| 1 | *Larix* | 45.33 | 106.84 | 10.50 | 0.51 | 8.61 | 0.76 | 195.11 | 6.03 | 259.54 | 6.37 |
| 2 | *Picea , Abies* forests in north China | 83.35 | 123.40 | 19.79 | 1.76 | 14.13 | 2.21 | 194.53 | 11.92 | 311.80 | 13.10 |
| 3 | *Pinus sylvestris* | 33.17 | 79.59 | 7.66 | 1.04 | 7.59 | 0.61 | 144.74 | 13.31 | 193.15 | 14.22 |
| 4 | *Picea , Abies* forests in South China | 101.53 | 132.50 | 19.80 | 1.41 | 6.90 | 1.57 | 240.72 | 19.57 | 368.96 | 20.65 |
| 5 | *Pinus koraiensis* | 70.28 | 126.14 | 15.92 | 1.24 | 10.67 | 1.01 | 192.75 | 12.88 | 289.63 | 14.07 |
| 6 | Coniferous evergreen and deciduous broadleaf  mixed forests on mountains in subtropical zone | 80.58 | 107.79 | 10.24 | 3.17 | 2.27 | 0.32 | 164.12 | 6.48 | 257.21 | 19.45 |
| 7 | *Platycladus orientalis* | 22.27 | 69.06 | 6.99 | 1.29 | 6.96 | 1.73 | 88.64 | 7.85 | 124.85 | 9.06 |
| 8 | *Pinus tabulaeformis* | 41.09 | 102.64 | 10.64 | 1.02 | 5.33 | 0.92 | 105.77 | 5.66 | 162.83 | 6.35 |
| 9 | *Pinus thunbergii* | 52.06 | 40.36 | 15.36 | 2.50 | 3.48 | 1.04 | 21.19 | 0.76 | 92.09 | 8.00 |
| 10 | Other coniferous forests in temperate zone | 31.74 | 50.42 | 17.58 | 6.26 | 5.27 | 2.05 | 67.42 | 7.88 | 122.01 | 11.25 |
| 11 | *Populus* | 31.58 | 79.40 | 7.65 | 0.64 | 2.06 | 0.29 | 73.41 | 4.62 | 114.69 | 5.25 |
| 12 | *Betula* | 36.27 | 131.67 | 10.03 | 0.62 | 4.86 | 0.61 | 193.16 | 20.08 | 244.31 | 20.20 |
| 13 | *Tilia tuan* | 72.04 | 140.35 | 27.03 | 16.93 | 3.87 | 0.02 | 166.03 | 23.72 | 268.98 | 32.72 |
| 14 | *Quercus* | 52.25 | 96.54 | 15.02 | 0.93 | 3.14 | 0.31 | 137.62 | 6.41 | 208.03 | 7.31 |
| 15 | *Fraxinus mandschurica, Juglans mandshurica,*  *Phellodendron amurense* | 37.92 | 113.41 | 6.87 | 2.37 | 5.31 | 0.26 | 183.89 | 4.13 | 233.99 | 7.89 |
| 16 | *Robinia pseudoacacia* | 26.27 | 69.07 | 9.11 | 1.36 | 2.30 | 0.82 | 54.84 | 4.19 | 92.52 | 5.45 |
| 17 | Deciduous microphyllous woodland in temperate zone | 17.91 | 39.51 | 4.20 | 0.90 | 2.46 | 0.21 | 19.16 | 6.32 | 43.73 | 7.25 |
| 18 | *Cupressus funebris* | 28.87 | 64.01 | 7.07 | 1.22 | 2.00 | 0.36 | 139.44 | 11.78 | 177.38 | 12.73 |
| 19 | *Pinus armandii* | 62.66 | 91.92 | 9.22 | 0.87 | 4.79 | 2.03 | 128.09 | 18.93 | 204.76 | 19.70 |
| 20 | *Cryptomeria fortunei* | 51.73 | 69.36 | 18.23 | 3.63 | 2.41 | 0.71 | 145.88 | 10.43 | 218.26 | 14.10 |
| 21 | *Pinus massoniana* | 52.30 | 98.19 | 9.11 | 0.52 | 4.07 | 0.30 | 120.45 | 4.58 | 185.93 | 5.42 |
| 22 | *Pinus yunnanensis*, *Pinus kesiya* | 39.59 | 58.75 | 7.40 | 0.96 | 2.32 | 0.46 | 131.54 | 15.08 | 180.85 | 16.21 |
| 23 | Other coniferous forests in subtropical and tropical zones | 43.64 | 89.44 | 10.07 | 1.20 | 5.73 | 0.58 | 122.30 | 9.77 | 181.74 | 11.13 |
| 24 | *Cunninghamia lanceolata* | 57.34 | 97.32 | 11.54 | 0.51 | 3.55 | 0.40 | 117.92 | 2.58 | 190.35 | 4.00 |
| 25 | *Metasequoia glyptostroboides* | 58.90 | 139.46 | 7.51 | 2.99 | 4.48 | 2.30 | 90.83 | 14.74 | 161.72 | 32.22 |
| 26 | Deciduous broadleaf forests in subtropical zone | 51.08 | 67.83 | 11.74 | 0.81 | 1.96 | 0.22 | 159.69 | 9.29 | 224.46 | 9.96 |
| 27 | Evergreen broadleaf and deciduous mixed forests  in subtropical zone | 55.59 | 106.67 | 10.22 | 1.43 | 3.52 | 0.27 | 206.83 | 20.92 | 276.16 | 25.79 |
| 28 | *Schima superb, Castanopsis, Cyclobalanopsis glauca* | 81.69 | 122.33 | 16.39 | 1.32 | 4.42 | 0.51 | 148.39 | 7.64 | 250.89 | 9.31 |
| 29 | *Phoebe, Machilus* | 66.47 | 103.05 | 16.19 | 1.66 | 1.74 | 0.32 | 145.25 | 13.11 | 229.64 | 15.45 |
| 30 | *Cinnamomum longepaniculatum* | 35.10 | 95.59 | 8.48 | 1.33 | 2.42 | 0.81 | 183.95 | 12.35 | 229.95 | 13.68 |
| 31 | Sclerophyllus broadleaf evergreen forests in subtropical zone | 74.36 | 55.24 | 22.95 | 0.65 | 3.78 | 0.47 | 107.86 | 2.27 | 208.95 | 2.47 |
| 32 | Other evergreen broadleaf forests in subtropical zone | 65.42 | 96.27 | 21.90 | 3.40 | 3.31 | 0.49 | 131.21 | 9.15 | 221.84 | 15.61 |
| 33 | *Eucalyptus* | 55.68 | 118.20 | 9.50 | 0.91 | 2.56 | 0.21 | 109.62 | 6.69 | 177.35 | 8.09 |
| 34 | *Casuarina equisetifolia* | 55.25 | 97.36 | 11.89 | 1.93 | 0.90 | 0.24 | 91.67 | 30.24 | 159.71 | 31.28 |
| 35 | *Acacia confusa* | 46.35 | 76.12 | 9.84 | 1.28 | 5.56 | 0.49 | 96.71 | 13.00 | 158.47 | 14.54 |
| 36 | *Bamboo forests* | 18.16 | 66.76 | 9.33 | 1.71 | 2.93 | 0.65 | 142.42 | 6.96 | 172.83 | 7.74 |
| 37 | Tropical monsoon rain forests | 126.67 | 57.65 | 28.33 | 2.70 | 6.79 | 1.10 | 95.93 | 9.50 | 257.72 | 11.29 |
| 38 | Tropical rain forests | 102.69 | 122.36 | 22.99 | 2.16 | 4.20 | 0.48 | 123.54 | 5.40 | 253.43 | 10.55 |
|  | Total | 57.15 | 101.65 | 13.00 | 1.51 | 4.67 | 0.60 | 148.75 | 9.48 | 223.57 | 10.84 |

† AGC, above ground vegetation biomass carbon density; BGC, below ground vegetation biomass carbon density, DMC, dead mass carbon density, SOC, soil organic carbon density in the 0-100cm soil layer.

**Appendix S10:** Carbon density and storage were estimated by 38 forest subtypes in China’s forest ecosystems (Continued)

| Code | Vegetation type | Area  (10^8^ha) | Carbon storage (Pg C) | | | | | | | | | |
| --- | --- | --- | --- | --- | --- | --- | --- | --- | --- | --- | --- | --- |
|  |  |  | AGC^†^ | | BGC | | DMC | | SOC | | Ecosystem | |
|  |  |  | Mean | SE | Mean | SE | Mean | SE | Mean | SE | Mean | SE |
| 1 | *Larix* | 0.1274 | 0.577 | 0.024 | 0.134 | 0.007 | 0.110 | 0.010 | 2.485 | 0.077 | 3.306 | 0.081 |
| 2 | *Picea , Abies* forests in north China | 0.0289 | 0.241 | 0.013 | 0.057 | 0.005 | 0.041 | 0.006 | 0.563 | 0.034 | 0.903 | 0.038 |
| 3 | *Pinus sylvestris* | 0.0037 | 0.012 | 0.002 | 0.003 | 0.000 | 0.003 | 0.000 | 0.054 | 0.005 | 0.072 | 0.005 |
| 4 | *Picea , Abies* forests in South China | 0.1414 | 1.436 | 0.088 | 0.280 | 0.020 | 0.098 | 0.022 | 3.405 | 0.277 | 5.218 | 0.292 |
| 5 | *Pinus koraiensis* | 0.0169 | 0.119 | 0.009 | 0.027 | 0.002 | 0.018 | 0.002 | 0.326 | 0.022 | 0.489 | 0.024 |
| 6 | Coniferous evergreen and deciduous broadleaf  mixed forests on mountains in subtropical zone | 0.0021 | 0.017 | 0.004 | 0.002 | 0.001 | 0.000 | 0.000 | 0.035 | 0.001 | 0.055 | 0.004 |
| 7 | *Platycladus orientalis* | 0.0068 | 0.015 | 0.003 | 0.005 | 0.001 | 0.005 | 0.001 | 0.060 | 0.005 | 0.085 | 0.006 |
| 8 | *Pinus tabulaeformis* | 0.0223 | 0.092 | 0.006 | 0.024 | 0.002 | 0.012 | 0.002 | 0.236 | 0.013 | 0.363 | 0.014 |
| 9 | *Pinus thunbergii* | 0.0032 | 0.017 | 0.002 | 0.005 | 0.001 | 0.001 | 0.000 | 0.007 | 0.000 | 0.029 | 0.003 |
| 10 | Other coniferous forests in temperate zone | 0.0066 | 0.021 | 0.003 | 0.012 | 0.004 | 0.003 | 0.001 | 0.045 | 0.005 | 0.081 | 0.007 |
| 11 | *Populus* | 0.0374 | 0.118 | 0.009 | 0.029 | 0.002 | 0.008 | 0.001 | 0.275 | 0.017 | 0.429 | 0.020 |
| 12 | *Betula* | 0.0885 | 0.321 | 0.018 | 0.089 | 0.006 | 0.043 | 0.005 | 1.710 | 0.178 | 2.162 | 0.179 |
| 13 | *Tilia tuan* | 0.0518 | 0.373 | 0.077 | 0.140 | 0.088 | 0.020 | 0.000 | 0.859 | 0.123 | 1.392 | 0.169 |
| 14 | *Quercus* | 0.2093 | 1.094 | 0.071 | 0.315 | 0.020 | 0.066 | 0.007 | 2.881 | 0.134 | 4.355 | 0.153 |
| 15 | *Fraxinus mandschurica, Juglans mandshurica,*  *Phellodendron amurense* | 0.0022 | 0.008 | 0.001 | 0.002 | 0.001 | 0.001 | 0.000 | 0.041 | 0.001 | 0.052 | 0.002 |
| 16 | *Robinia pseudoacacia* | 0.0120 | 0.032 | 0.004 | 0.011 | 0.002 | 0.003 | 0.001 | 0.066 | 0.005 | 0.111 | 0.007 |
| 17 | Deciduous microphyllous woodland in temperate  zone | 0.0328 | 0.059 | 0.011 | 0.014 | 0.003 | 0.008 | 0.001 | 0.063 | 0.021 | 0.143 | 0.024 |
| 18 | *Cupressus funebris* | 0.0047 | 0.014 | 0.002 | 0.003 | 0.001 | 0.001 | 0.000 | 0.066 | 0.006 | 0.084 | 0.006 |
| 19 | *Pinus armandii* | 0.0133 | 0.083 | 0.007 | 0.012 | 0.001 | 0.006 | 0.003 | 0.170 | 0.025 | 0.272 | 0.026 |
| 20 | *Cryptomeria fortunei* | 0.0023 | 0.012 | 0.002 | 0.004 | 0.001 | 0.001 | 0.000 | 0.033 | 0.002 | 0.050 | 0.003 |
| 21 | *Pinus massoniana* | 0.3162 | 1.654 | 0.090 | 0.288 | 0.016 | 0.129 | 0.009 | 3.809 | 0.145 | 5.879 | 0.172 |
| 22 | *Pinus yunnanensis*, *Pinus kesiya* | 0.0748 | 0.296 | 0.044 | 0.055 | 0.007 | 0.017 | 0.003 | 0.984 | 0.113 | 1.352 | 0.121 |
| 23 | Other coniferous forests in subtropical and  tropical zones | 0.0010 | 0.004 | 0.001 | 0.001 | 0.000 | 0.001 | 0.000 | 0.012 | 0.001 | 0.018 | 0.001 |
| 24 | *Cunninghamia lanceolata* | 0.0572 | 0.328 | 0.017 | 0.066 | 0.003 | 0.020 | 0.002 | 0.674 | 0.015 | 1.088 | 0.023 |
| 25 | *Metasequoia glyptostroboides* | 0.0008 | 0.005 | 0.002 | 0.001 | 0.000 | 0.000 | 0.000 | 0.007 | 0.001 | 0.013 | 0.003 |
| 26 | Deciduous broadleaf forests in subtropical zone | 0.0363 | 0.185 | 0.013 | 0.043 | 0.003 | 0.007 | 0.001 | 0.579 | 0.034 | 0.814 | 0.036 |
| 27 | Evergreen broadleaf and deciduous mixed  Forests in subtropical zone | 0.0171 | 0.095 | 0.026 | 0.017 | 0.002 | 0.006 | 0.000 | 0.353 | 0.036 | 0.472 | 0.044 |
| 28 | *Schima superb, Castanopsis,*  *Cyclobalanopsis glauca* | 0.1088 | 0.889 | 0.056 | 0.178 | 0.014 | 0.048 | 0.006 | 1.614 | 0.083 | 2.715 | 0.101 |
| 29 | *Phoebe, Machilus* | 0.0003 | 0.002 | 0.000 | 0.000 | 0.000 | 0.000 | 0.000 | 0.004 | 0.000 | 0.007 | 0.000 |
| 30 | *Cinnamomum longepaniculatum* | 0.0006 | 0.002 | 0.000 | 0.001 | 0.000 | 0.000 | 0.000 | 0.011 | 0.001 | 0.014 | 0.001 |
| 31 | Sclerophyllus broadleaf evergreen forests  in subtropical zone | 0.0157 | 0.117 | 0.001 | 0.036 | 0.001 | 0.006 | 0.001 | 0.170 | 0.004 | 0.329 | 0.004 |
| 32 | Other evergreen broadleaf forests  in subtropical zone | 0.0107 | 0.070 | 0.013 | 0.023 | 0.004 | 0.004 | 0.001 | 0.141 | 0.010 | 0.238 | 0.017 |
| 33 | *Eucalyptus* | 0.0029 | 0.016 | 0.001 | 0.003 | 0.000 | 0.001 | 0.000 | 0.032 | 0.002 | 0.052 | 0.002 |
| 34 | *Casuarina equisetifolia* | 0.0005 | 0.003 | 0.000 | 0.001 | 0.000 | 0.000 | 0.000 | 0.005 | 0.002 | 0.009 | 0.002 |
| 35 | *Acacia confusa* | 0.0010 | 0.005 | 0.001 | 0.001 | 0.000 | 0.001 | 0.000 | 0.010 | 0.001 | 0.016 | 0.001 |
| 36 | *Bamboo forests* | 0.0329 | 0.060 | 0.009 | 0.031 | 0.006 | 0.010 | 0.002 | 0.469 | 0.023 | 0.569 | 0.026 |
| 37 | Tropical monsoon rain forests | 0.0062 | 0.079 | 0.003 | 0.018 | 0.002 | 0.004 | 0.001 | 0.059 | 0.006 | 0.160 | 0.007 |
| 38 | Tropical rain forest | 0.0186 | 0.191 | 0.016 | 0.043 | 0.004 | 0.008 | 0.001 | 0.230 | 0.010 | 0.472 | 0.020 |
|  | Total | 1.5155 | 8.661 | 0.649 | 1.971 | 0.229 | 0.708 | 0.091 | 22.542 | 1.437 | 33.882 | 1.643 |
